# Immunosuppressive Isopimarane Diterpenes From Cultures of the Endophytic Fungus *Ilyonectria robusta*

**DOI:** 10.3389/fphar.2021.766441

**Published:** 2022-01-10

**Authors:** Ke Ye, Xiao Lv, Xian Zhang, Pan-Pan Wei, Zheng-Hui Li, Hong-Lian Ai, Da-Ke Zhao, Ji-Kai Liu

**Affiliations:** ^1^ South Central University for Nationalities, Wuhan, China; ^2^ Yunnan University, Kunming, China

**Keywords:** immunosuppressive activity, endophytic fungus, natural products, isopimarane diterpenes, ECD calculation

## Abstract

Five new isopimarane diterpenes, robustaditerpene A-E (**1**–**5**), which include 19-nor-isopimarane skeleton and isopimarane skeleton, were isolated from the liquid fermentation of the endophytic fungus *Ilyonectria robusta* collected from *Bletilla striata.* The structure elucidation and relative configuration assignments of all compounds were accomplished by interpretation of NMR and HRESIMS spectrometric analyses and ^13^C NMR calculation. And the absolute configuration of **1**-**5** were identified by single-crystal X-ray diffraction and ECD calculation. Compound **3** inhibited lipopolysaccharide-induced B lymphocytes cell proliferation with an IC_50_ value at 17.42 ± 1.57 *μ*M while compound **5** inhibited concanavalin A-induced T lymphocytes cell proliferation with an IC_50_ value at 75.22 ± 6.10 *μ*M. These data suggested that compounds **3** and **5** may possess potential immunosuppressive prospect.

## Introduction

Immunosuppressive agents are a significant class of clinical drugs for organic transplantation and treatment of autoimmune diseases, such as rheumatoid arthritis, systemic lupus, multiple sclerosis, myasthenia gravis, and pemphigus (Dangroo et al., 2016). Although excellent advantages were taken, these immunosuppressive agents have some inevitable and serious side effects including the renal and liver toxicity, infection, malignancy, and others (Smith et al., 2003). Therefore, it is a significant requirement to develop new, efficient, and safe immunosuppressive agents.

Natural products play a highly important role in the drug discovery and development process. Many clinically applied drugs derived from or were natural products (Newman and Cragg, 2016). Several immunosuppressive agents, such as mycophenolic acid, cyclosporin A, tacrolimus, sirolimus, corticosteroids and so on, were also generated from natural products (Kahan, 2003; Wang et al., 2019).

Endophytic fungi are a rich source of natural products (Ye et al., 2021). The number of natural products from endophytic fungi are more than any other endophytic microorganism class, and they have various structures and a broad spectrum of bioactivities. Furthermore, natural products from endophytic fungi have various, even novel structures, which can be grouped into several types including alkaloids, steroids, terpenoids, quinones and so on (Tan and Zou, 2001; Zhang et al., 2006). One of the largest groups of bioactive natural products that have been identified is the terpenoids. Diterpenoids derived from C20 precursor (*E*, *E*, *E*)-geranylgeranyl diphosphate with more than 12,000 described compounds (Quin et al., 2014; Zi et al., 2014). Some isopimarane diterpenes with immunosuppressive activity have been reported (Chen et al., 2020).

**FIGURE 1:**
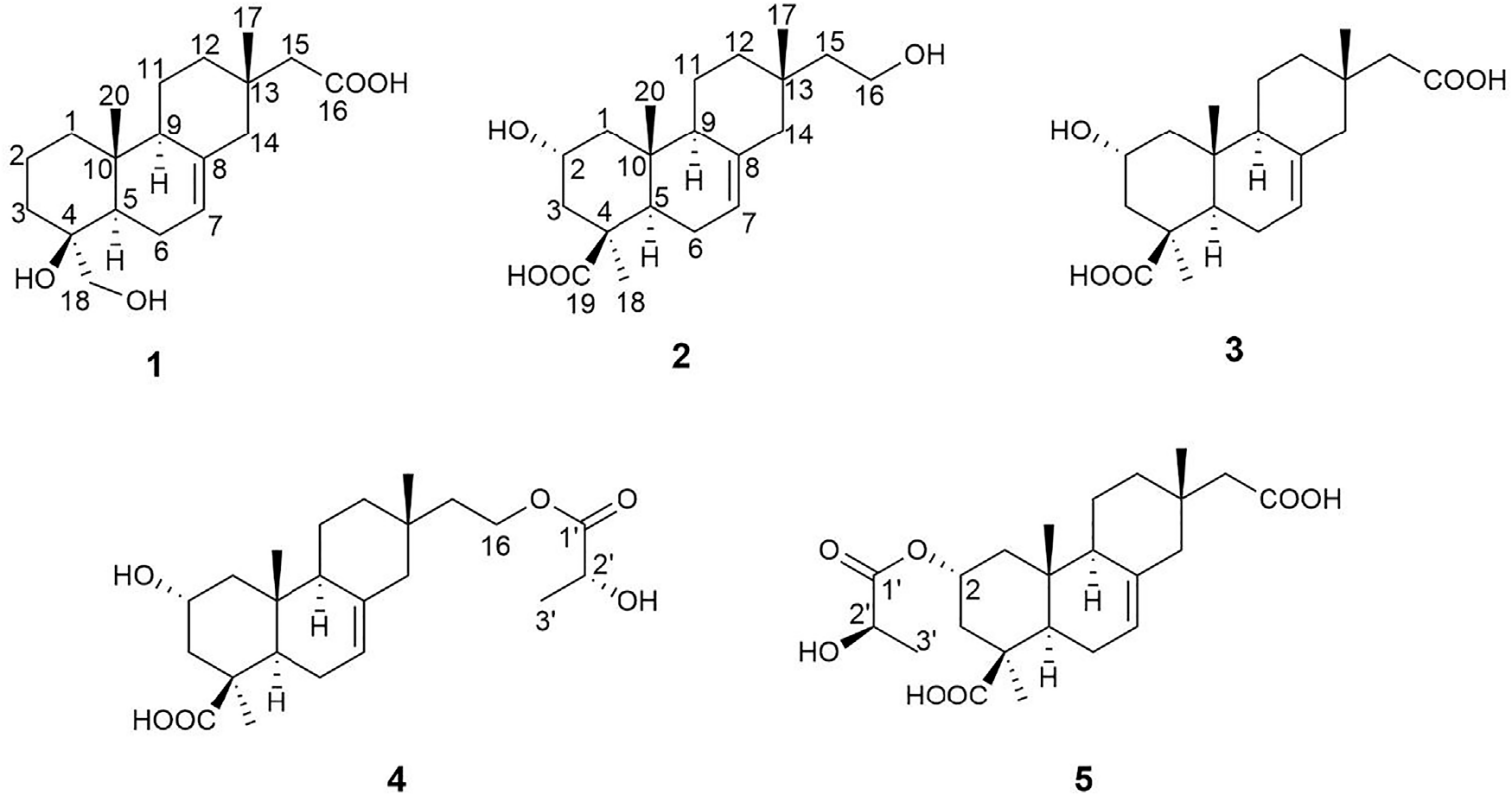
Structures of compounds **1**-**5**.

For searching the leading compounds with immunosuppressive activity, we studied an endophytic fungus *Ilyonectria robusta*, isolated from the medicinal plant *Bletilla striata*. Five new isopimarane diterpenes (**1**–**5**) (Figure 1) were obtained from the crude extract of this fungus. Biological evaluation suggested that some compounds displayed immunosuppressive activity against T and B lymphocytic cell proliferation. Herein, we describe isolation, elucidation, and biological activity of these isopimarane diterpenes.

## Materials and Methods

### Fungal Material

The strain *Ilyonectria robusta* was isolated from the rhizome of *Bletilla striata* collected from Enshi, Hubei province, and was identified as *Ilyonectria robusta* via 18S rDNA-sequence and deposited at South-Central University for Nationalities, China. The sequence data for this strain had been submitted to the DDBJ/EMBL/Genbank with accession No. JX045819.1.

### Fungal Fermentation, Extraction and Isolation

The strain *Ilyonectria robusta* was cultured on potato dextrose agar medium at 25°C for 5 days to prepare the seed culture. The agar plugs were cut into small pieces and inoculated to 500 ml Erlenmeyer flasks each containing 300 ml of liquid medium (5% glucose, 0.15% peptone from porcine meat, 0.5% yeast extract, 0.05% KH_2_PO_4_, and 0.05% MgSO_4_. Total volume was 50 L) and fermented on a rotatory shaker at 25°C and 160 rpm for a month.

The culture broth (50 L) of *Ilyonectria robusta* was centrifuged to separate the mycelium and liquid cultures. The liquid layer was concentrated under reduced pressure to afford 8 L of extract and further partitioned with ethyl acetate 24 L for three times at room temperature. The mycelia were soaked with MeOH (total 6 L) at room temperature. The extract was evaporated under reduced pressure, resolved in water (2 L), and further extracted with ethyl acetate 6 L three times.

Both ethyl acetate solutions were combined and evaporated under reduced pressure to yield 169.1 g crude extract. The crude extract was fractioned by column chromatography (CC) over silica gel using a chloroform-methanol system (0–100%) to give 5 fractions (A-E). Fraction C (10 g) was chromatographed further by MPLC with stepwise gradient of MeOH-H_2_O (0–100%) to afford 12 fractions (C1-C12). Fraction C8 (0.5 g) was subjected to silica gel CC (petroleum ether–acetone from v/v 5:1 to 0:1) and yielded eight subfractions (C8-1-C8-5). Subfraction C8-5 (0.2 g) was purified by prep-HPLC (MeCN-H_2_O: 28%–36%, 18min, 4 ml/min) to give compound **2** (*t*_R_ = 12.0 min, 5.3 mg) and **3** (*t*_R_ = 14.0 min, 19.9 mg). Fraction C9 (0.6 g) was subjected to silica gel CC (petroleum ether–acetone from v/v 6:1 to 0:1) and yield seven subfractions (C9-1-C9-7). Subfraction C9-3 (0.1 g) was isolated by prep-HPLC (MeCN-H_2_O: 24%–28%, 18 min, 4 ml/min) to give compound **1** (*t*_R_ = 11.5 min, 4.9 mg). Subfraction C9-5 (0.2 g) was purified by prep-HPLC (MeOH-H_2_O: 50%–58%, 20 min, 4 ml/min) to yield compound **4** (*t*_R_ = 12.4 min, 11.3 mg) and **5** (*t*_R_ = 13.1 min, 5.4 mg).

### General Experimental Procedures

Optical rotations were measured on a Rudolph Autopol IV polarimeter. UV spectra were obtained on a UH5300 UV-VIS Double Beam Spectrophotometer. IR spectra were obtained by using a Shimadu Fourier Transform Infrared spectrophotometer with KBr pellets. 1D and 2D NMR spectra were run on Bruker Avance III 600 MHz and Bruker Avance NEO 500 MHz spectrometer with TMS as an internal standard. X-ray crystallographic analysis was on the Bruker D8 QUEST. High Resolution Electrospray Ionization Mass Spectra (HRESIMS) were recorded on a Thermo scientific Q Exactive Orbitrap MS system. Column chromatography (CC) was performed on silica gel (200–300 mesh, Qingdao Marine Chemical Ltd., Qingdao, People’s Republic of China), RP-18 gel (20–45 *μ*m, Fuji Silysia Chemical Ltd., Japan), and Sephadex LH-20 (Pharmacia Fine Chemical Co., Ltd., Sweden). Medium Pressure Liquid Chromatography (MPLC) was performed on a Biotage SP1 equipment, and columns packed with RP-18 gel. Preparative High Performance Liquid Chromatography (prep-HPLC) was performed on an Agilent 1260 liquid chromatography system equipped with Zorbax SB-C18 columns (5 *μ*m, 9.4 × 150 mm or 21.2 × 150 mm, flow rate 4 ml/min) and a DAD detector. Fractions were monitored by TLC (GF 254, Qingdao Haiyang Chemical Co., Ltd. Qingdao), and spots were visualized by heating silica gel plates sprayed with 10% H_2_SO_4_ in EtOH. A Cell Counting Kit-8 assay kit was used to measure cell viability (Dojindo, Kumamoto, Japan).

### Spectroscopic Characterization of Compounds 1-5

Robustaditerpene A (**1**): colorless acicular crystal (acetone/MeOH). [α]^17^_D_ -12.8 (*c* 0.10, MeOH); UV (MeOH) *λ*_max_ (log *ε*) 210.0 nm (3.86); IR (KBr) *ν*_max_ 3350.35, 2945.30, 2833.43, 1454.33, 1031.92 cm^−1^; ^1^H NMR (500 MHz, Methanol-*d*_4_) and ^13^C NMR (125 MHz, Methanol-*d*_4_) data, see Table 1; positive ion HRESIMS *m/z* 345.20358 [M + Na]^+^ (calcd for C_19_H_30_O_4_Na 345.20363).

**TABLE 1:** ^1^H NMR and^13^C NMR spectroscopic data for 1–3 (Methanol-*d*_4_).

No	1		2		3	
	*δ*_C_, type (125 MHz)	*δ*_H_ (*J* in Hz) (500 MHz)	*δ*_C_, type (125 MHz)	*δ*_H_ (*J* in Hz) (500 MHz)	*δ*_C_, type (150 MHz)	*δ*_H_ (*J* in Hz) (600 MHz)
1	40.2, CH_2_	1.86, m	49.6, CH_2_	2.13, m	49.5, CH_2_	2.13, m
		1.03, m		0.96, m		0.98, m
2	18.5, CH_2_	1.84, m	65.3, CH	4.16, m	65.3, CH	4.16, m
		1.46, overlapped				
3	36.4, CH_2_	1.57, m	47.8, CH_2_	2.34, m	47.7, CH_2_	2.35, m
				0.99, m		1.01, m
4	74.4, C		46.2, C		46.1, C	
5	44.4, CH	1.45, overlapped	52.1, CH	1.30, dd (12.4, 4.1)	52.0, CH	1.32, dd (12.1, 4.3)
6	23.2, CH_2_	2.07, m	25.4, CH_2_	2.36, m	25.4, CH_2_	2.37, m
		1.92, m		2.15, m		2.17, m
7	122.5, CH	5.36, m	122.4, CH	5.37, m	122.7, CH	5.39, m
8	136.8, C		136.0, C		135.7, C	
9	52.3, CH	1.68, m	53.0, CH	1.73, m	52.7, CH	1.75, br s
10	36.0, C		38.4, C		38.4, C	
11	21.3, CH_2_	1.56, m	22.1, CH_2_	1.59, m	22.0, CH_2_	1.62, m
		1.37, overlapped		1.34, m		1.37, overlapped
12	38.0, CH_2_	1.63, m	38.3, CH_2_	1.54, m	38.0, CH_2_	1.66, m
		1.37, overlapped		1.27, m		1.37, overlapped
13	34.6, C		33.9, C		34.6, C	
14	48.1, CH_2_	2.01, m	48.4, CH_2_	1.91, m	47.8, CH_2_	2.02, m
15	50.6, CH_2_	2.13, s	48.5, CH_2_	1.47, m	49.9, CH_2_	2.15, s
16	176.8, C		59.1, CH_2_	3.64, m	176.0, C	
17	22.1, CH_3_	0.91, s	22.1, CH_3_	0.79, s	22.0, CH_3_	0.90, s
18	69.2, CH_2_	3.43, d (10.9)	29.7, CH_3_	1.25, s	29.6, CH_3_	1.26, s
		3.13, d (10.9)				
19			180.9, C		180.8, C	
20	15.6, CH_3_	1.07, s	15.5, CH_3_	0.79, s	15.5, CH_3_	0.80, s

Robustaditerpene B (**2**): white powder. [α]^17^_D_ -1.2 (*c* 0.10, MeOH); UV (MeOH) *λ*_max_ (log *ε*) 205.0 nm (3.66); IR (KBr) *ν*_max_ 3350.35, 2945.30, 2831.50, 1452.40, 1031.92 cm^−1^; ^1^H NMR (600 MHz, Methanol-*d*_4_) and ^13^C NMR (150 MHz, Methanol-*d*_4_) data, see Table 1; negative ion HRESIMS *m/z* 335.22449 [M–H]^–^ (calcd for C_20_H_31_O_4_ 335.22278).

Robustaditerpene C (**3**): white powder. [α]^17^_D_ -3.16 (c 0.16, MeOH); UV (MeOH) *λ*_max_ (log *ε*) 205.0 nm (2.76); IR (KBr) *ν*_max_ 3358.07, 2945.30, 2831.50, 1454.33, 1031.92 cm^−1^; ^1^H NMR (500 MHz, Methanol-*d*4) and ^13^C NMR (125 MHz, Methanol-*d*_4_) data, see Table 1; negative ion HRESIMS *m/z* 349.20373 [M–H]^–^ (calcd for C_20_H_29_O_5_ 349.20205).

Robustaditerpene D (**4**): yellow amorphous solid. [α]^17^_D_ +1.33 (c 0.10, MeOH); UV (MeOH) *λ*_max_ (log *ε*) 205.0 nm (3.81); IR (KBr) *ν*_max_ 3350.35, 2945.30, 2833.43, 1454.33, 1031.92 cm^−1^; ^1^H NMR (500 MHz, Methanol-*d*_4_) and ^13^C NMR (125 MHz, Methanol-*d*_4_) data, see Table 2; positive ion HRESIMS *m/z* 431.24045 [M + Na]^+^ (calcd for C_23_H_36_O_6_Na 431.24096).

**TABLE 2:** ^1^H NMR (500 MHz) and^13^C NMR (125 MHz) spectroscopic data for 4 and 5 (Methanol-*d*_4_).

No	4		5	
	*δ*_C_, type	*δ*_H_ (*J* in Hz)	*δ*_C_, type	*δ*_H_ (*J* in Hz)
1	49.5, CH_2_	2.13, m	45.6, CH_2_	2.22, m
		0.97, m		1.12, m
2	65.3, CH	4.16, m	70.8, CH	5.43, m
3	47.7, CH_2_	2.35, m	43.8, CH_2_	2.34, m
		1.00, m		1.25, m
4	46.2, C		46.2, C	
5	52.0, CH	1.30, overlapped	51.8, CH	1.40, dd (12.1, 4.0)
6	25.4, CH_2_	2.37, m	25.3, CH_2_	2.36, m
		2.17, m		2.18, m
7	122.6, CH	5.39, m	122.6, CH	5.41, m
8	135.8, C		135.7, C	
9	52.9, CH	1.75, m	52.5, CH	1.78, br s
10	38.4, C		38.6, C	
11	22.0, CH_2_	1.61, m	22.0, CH_2_	1.56, m
		1.34, m		1.36, overlapped
12	38.1, CH_2_	1.55, m	37.9, CH_2_	1.65, m
		1.30, overlapped		1.36, overlapped
13	34.1, C		34.6, C	
14	48.1, CH_2_	1.94, m	47.7, CH_2_	2.03, m
15	44.1, CH_2_	1.57, t (7.3)	49.9, CH_2_	2.15, s
16	62.8, CH_2_	4.23, m	175.9, C	
17	21.9, CH_3_	0.82, s	22.0, CH_3_	0.90, s
18	29.6, CH_3_	1.26, s	29.3, CH_3_	1.28, s
19	180.8, C		180.3, C	
20	15.5, CH_3_	0.80, s	15.1, CH_3_	0.86, s
1′	176.4, C		176.0, C	
2′	67.9, CH	4.22, m	68.0, CH	4.20, m
3′	20.5, CH_3_	1.36, d (6.9)	20.6, CH_3_	1.37, overlapped

Robustaditerpene E (**5**): yellow amorphous solid. [α]^17^_D_ -26.86 (*c* 0.11, MeOH); UV (MeOH) *λ*_max_ (log *ε*) 205.0 nm (3.65); IR (KBr) *ν*_max_ 3338.78, 2945.30, 2831.50, 1452.40, 1031.92 cm^−1^; ^1^H NMR (500 MHz, Methanol-*d*_4_) and ^13^C NMR (125 MHz, Methanol-*d*_4_) data, see Table 2; positive ion HRESIMS *m/z* 445.21960 [M + Na]^+^ (calcd for C_23_H_34_O_7_Na 445.22022).

### X-Ray Crystallographic Analysis of Robustaditerpene A (1)

A block-like specimen of C_19_H_30_O_4_, M = 322.43, approximate dimensions 0.153 × 0.214 × 0.312 mm, was used for the X-ray crystallographic analysis on the Bruker D8 QUEST. The integration of the data using an orthorhombic unit cell yielded a total of 43,720 reflections to a maximum *θ* angle of 79.36° (0.78 Å resolution), of which 3,851 were independent (average redundancy 11.353, completeness = 99.8%, *R*_int_ = 3.83%, *R*_sig_ = 1.94%) and 3,788 (98.65%) were greater than 2*σ* (*F* ^2^). The final cell constants of *a* = 5.8402 (2) Å, *b* = 12.6315 (5) Å, *c* = 24.0621 (9) Å, *α* = 90.00°, *β* = 90.00°, *γ* = 90.00°, *V* = 1775.07 (11) Å^3^, *T* = 298 (2) K. Data were corrected for absorption effects using the Multi-Scan method (SADABS). The structure was solved and refined using the Bruker SHELXTL Software Package (Sheldrick, 2008), using the space group *P* 21 21 21, *Z* = 4, *μ* (Cu Kα) = 1.54178. The final anisotropic full-matrix least-squares refinement on *F* ^2^ with 216 variables converged at *R*_1_ = 2.90%, for the observed data and *wR*_2_ = 8.00% for all data. The goodness of fit was 1.043. The absolute configuration was determined by the Flack parameter = 0.01 (3), which was determined using 1,597 quotients [(*I*+)—(*I*-)]/[(*I*+) + (*I*-)]. Crystallographic data for the structure of **1** were deposited at the Cambridge Crystallographic Data Centre (deposition number: 2105380).

### Quantum Chemical Calculation Methods

Systematic conformational analyses were performed by SYBYL-X 2.1.1 based on molecular mechanics with MMFF94s force field (Goto and Osawa, 1989). The conformers with a distribution higher than 1% will be further optimized. The optimization and frequency of conformers were calculated on B3LYP/6-31G(d) level of theory with the IEF-PCM solvent model (MeOH) in the Gaussian09 software package (Frisch et al., 2013).

#### ^13^C NMR Calculation

After analyzing and optimizing the conformer candidates of (2′R*)-**4** and (2′S*)-**4**, the conformers within 4 kcal/mol of the global minimum were selected for further ^13^C NMR calculations. Gauge-independent atomic orbital (GIAO) calculations of the shielding values of (2′R*)-**4** and (2′S*)-**4** were accomplished by the time-dependent density functional theory (TDDFT) at the *ω*B97x-D/6-31G(d) level with IEF-PCM solvent model (MeOH) in the Gaussian09 software package (Frisch et al., 2013). The calculated NMR data of these conformers were averaged according to the Boltzmann distribution theory and their relative Gibbs free energies. The linear correlation coefficient (*R* ^2^) and root-mean-square deviation (RMSD) were calculated for the evaluation of the deviations between the experimental and calculated results. The calculation results were processed by an in-house Excel-based program.

#### ECD Calculation

The optimized conformers were selected for further electronic circular dichroism (ECD) calculation. The ECD (TDDFT) calculation of all conformations was performed on B3LYP/6-311G(d) Level of theory with IEF-PCM solvent model (MeOH). All the DFT calculations were performed by Gaussian09 software package (Turney et al., 2012). The calculated and weighted ECD curve were all generated by SpecDisv 1.71 (Bruhn et al., 2013), respectively.

### T and B Cell Proliferation Inhibitory Activity Assay

All the compounds were subjected to evaluate their inhibition on the proliferation of T and B lymphocytes. Fresh spleen cells were obtained from female BALB/c mice (18–20 g). Spleen cells (1 × 10^6^ cells) were seeded in triplicate in 96-well flat plates and cultured at 37°C for 48 h in 96-well flat plates, in the presence or absence of various concentrations of compounds, in a humidified and 5% CO_2_-containing incubator. A certain amount of CCK-8 was added to each well at the final 8–10 h of culture. To the end of the culture, the OD values with a microplate reader (Bio-Rad 650) were measured at 450 nm. If the cell viability was higher than 85%, the compound was further screened for its suppressive activity against the T and B lymphocytes. Fresh spleen cells were obtained from female BALB/c mice (18–20 g). The 5×10^5^ spleen cells were cultured at the same conditions as those mentioned above. The cultures, in the presence or absence of various concentrations of compounds, were stimulated with 5 *μ*g/ml of ConA or 10 *μ*g/ml of LPS to induce T cells or B cells proliferative responses, respectively. Proliferation was assessed in terms of uptake of [^3^H]-thymidine during 8 h of pulsing with 25*μ*L/well of [^3^H]-thymidine, and then cells were harvested onto glass fiber filters. The incorporated radioactivity was counted using a Beta scintillation counter (MicroBeta Trilux, PerkinElmer Life Sciences). Cells treated without any stimuli were used as the negative control. The immunosuppressive activity of each compound was expressed as the concentration of compound that inhibited ConA-induced T cell proliferation or LPS-induced B cell proliferation to 50% (IC_50_) of the control value. The studies involving animals were reviewed and approved by Animal Ethics Committee of South-Central University for Nationalities (SYXK (Wuhan) 2016-0089, No. 2021-SCUEC-AEC-033).

## Results and Discussion

### Structure Elucidations

**FIGURE 2:**
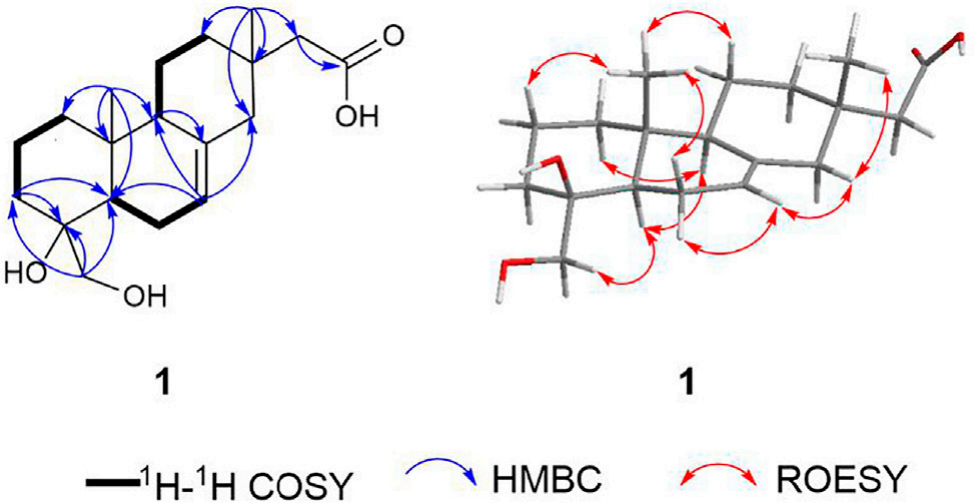
Key 2D NMR correlations of compound **1**.

**FIGURE 3:**
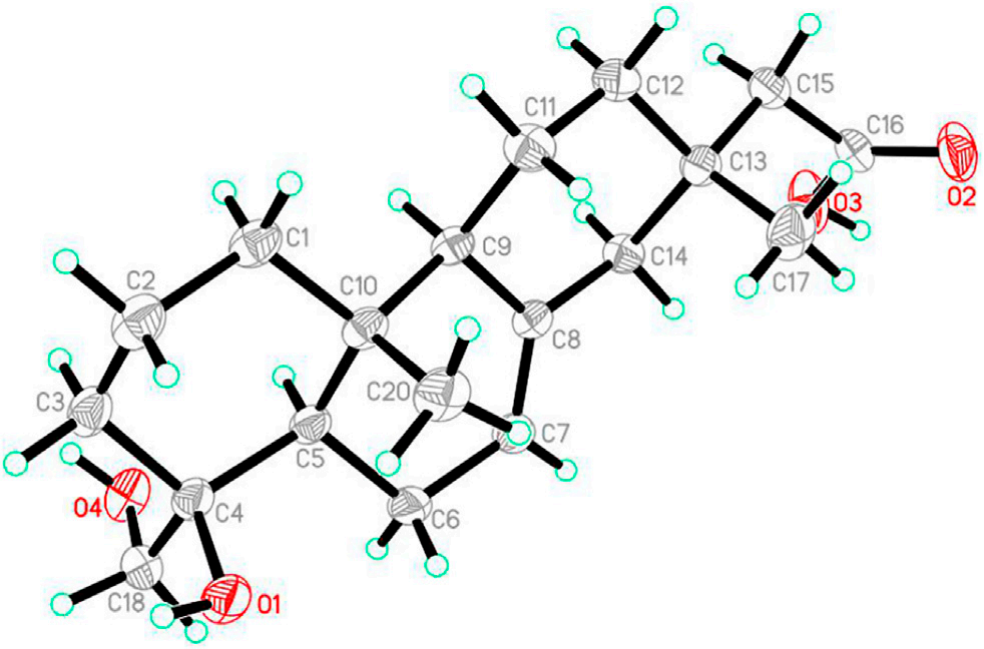
OPTER drawing of compound **1**.

Robustaditerpene A (**1**) was obtained as a colorless acicular crystal (acetone/MeOH). It has a molecular formula of C_19_H_30_O_4_ as determined by (+)-HRESIMS analysis, corresponding to five degrees of unsaturation. The 1D NMR (Table 1) showed signals ascribe to two methyl singlets at *δ*_H_ 0.91 (Me-17), 1.07 (Me-20), nine sp^3^ methylenes including one oxygenated carbon [*δ*_c_ 18.5 (C-2), 21.3 (C-11), 23.2 (C-6), 36.4 (C-3), 38.0 (C-12), 40.2 (C-1), 48.1 (C-14), 50.6 (C-15), 69.2 (C-18)], two sp^3^ methines [*δ*_c_ 44.4 (C-5), 52.3 (C-9)], two trisubstituted olefin bond carbons at *δ*_c_ 122.5 (C-7) and 136.8 (C-8), and a carbonyl carbon at *δ*_c_ 176.8 (C-16), accounting for two degree of five unsaturation, which indicated the presence of three rings in **1**. NMR analysis indicated that one was a diterpene-related compound. According to the 2D NMR spectra, both its planar structure and relative configuration were confirmed. The ^1^H–^1^H COSY showed signals of H-1/H-2/H-3, H-5/H-6/H-7, and H-9/H-11/H-12, allowing the connections as shown by bold lines in Figure 2. The HMBC correlations from Me-20 to C-1, C-5, C-9, and C-10, in combination with the HMBC correlations from H-3 to C-4 and C-5, from H-9 to C-8, revealed the presence of two six-membered rings, the ring A and ring B. Furthermore, the key HMBC correlations from Me-17 to C-12, C-13, C-14, and C-15, and from H-7 to C-14 constructed ring C. The additional hydroxymethyl was assigned to substitute at C-4 by the HMBC correlations from the protons at *δ*_H_ 3.43/3.13 to C-3, C-4 and C-5. And the HMBC correlations from H-15 to C-16 confirmed the location of carbonyl. The diagnostic ROESY correlations of Me-20/H-2*β*, Me-20/H-6*β*, Me-20/H-11*β*/Me-17/H-14/H-7/H-6*α*, and H-1/H-5/H-9/CH_2_OH helped to determine the relative configuration of **1**, indicating its skeleton to be 19-nor-isopimarane or *ent*-18-nor-isopimarane. The absolute configuration of one was determined by single-crystal X-ray diffraction analysis with a Flack parameter of 0.01 (3) (Figure 3). Thus, compound **1** was characterized as a 19-nor-isopimarane derivative.

**FIGURE 4:**
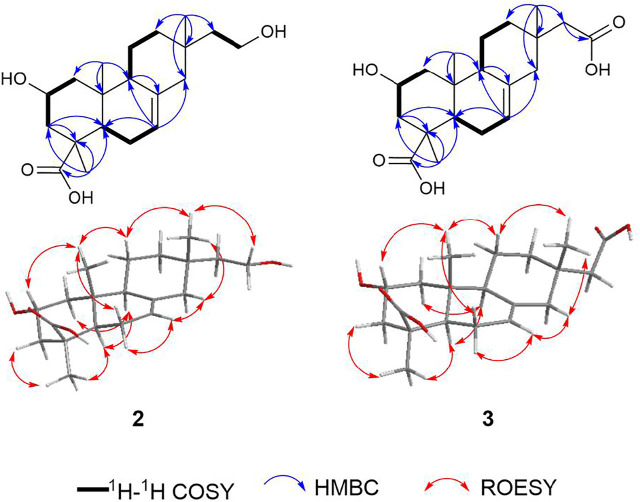
Key 2D NMR correlations of compound **2** and **3**.

**FIGURE 5:**
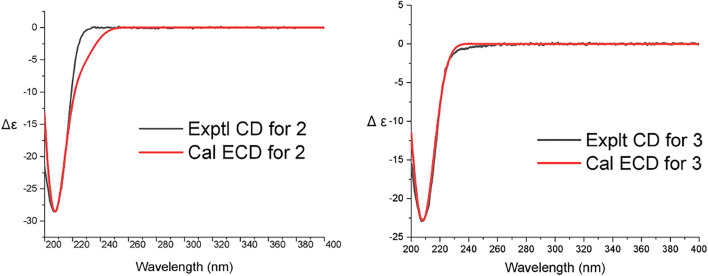
ECD calculation of compound **2** and **3.**

Robustaditerpene B (**2**) was isolated as a white powder. Its molecular formula is C_20_H_32_O_4_ as determined by (-)-HRESIMS analysis (*m*/*z* 335.22449 [M–H]^–^), corresponding to five degree of unsaturation. The 1D NMR spectra (Table 1) displayed three methyls, eight methylenes, three methines, five quaternary carbons including a carbonyl carbon, and a trisubstituted olefin double bond. The 1D NMR data of **2** were similar to those of 16-hydroxyisopimar-7-en-19-oic acid (Shiono et al., 2009), indicating skeleton of **2** to be isopimarane. However, a significant difference between **2** and 16-hydroxyisopimar-7-en-19-oic acid was that the presence of an additional oxymethine at *δ*_H_ 4.16 (1H, m) in **2** replaced the methylene at C-2 in 16-hydroxyisopimar-7-en-19-oic acid. The location of the hydroxy group at C-2 was determined by the HMBC correlations (Figure 4) from Me-20 to C-1, C-5, C-9, and C-10 and the ^1^H–^1^H COSY correlations (Figure 4) of H-1 (*δ*_H_ 0.96/2.13)/H-2 (*δ*_H_ 4.16)/H-3 (*δ*_H_ 0.99/2.34). The ROESY correlations of Me-20/H-2*β*, Me-20/H-6*β*, Me-20/H-11*β*/Me-17/H-14/H-7/H-6*α*, and Me-18/H-3/H-5/H-9 helped to determine the *α*-orientation of the hydroxy group at C-2 and the relative configuration of **2**. The absolute configuration of two was determined by comparing the calculated ECD with that of the experimental CD, which displayed a similar tendency to that shown in Figure 5. Thus, compound **2** was determined to be an isopimarane diterpene.

The molecular formula of Robustaditerpene C (**3**), a white powder, was determined as C_20_H_30_O_5_ by (-)-HRESIMS (*m/z* 349.20373 [M–H]^–^). Analysis of the 1D NMR data (Table 1) of **3** suggested that it was an isopimarane derivative bearing the structural similarities to **2**, except that the oxygenated methylene at δ_c_ 59.1(C-16) in two became a carboxyl group at δ_c_ 176.0 (C-16) in **3**. This finding was supported by the HMBC correlations (Figure 4) from H-15 (δ_H_ 2.15) to C-16 (δ_c_ 176.0), and from Me-17 (δ_H_ 0.90) to C-12 (δ_c_ 38.0), C-13 (δ_c_ 34.6), C-14 (δ_c_ 47.8), and C-15 (δ_c_ 49.9). The ROESY analysis (Figure 4) indicated relative configuration of **3** was the same as that of **2**. Biosynthetically, compounds **2** and **3** shared the same isopimarane skeleton, and the absolute configuration was determined based on the comparison of calculated ECD and experimental CD spectra (Figure 5). The structure of **3** was assigned as shown.

**FIGURE 6:**
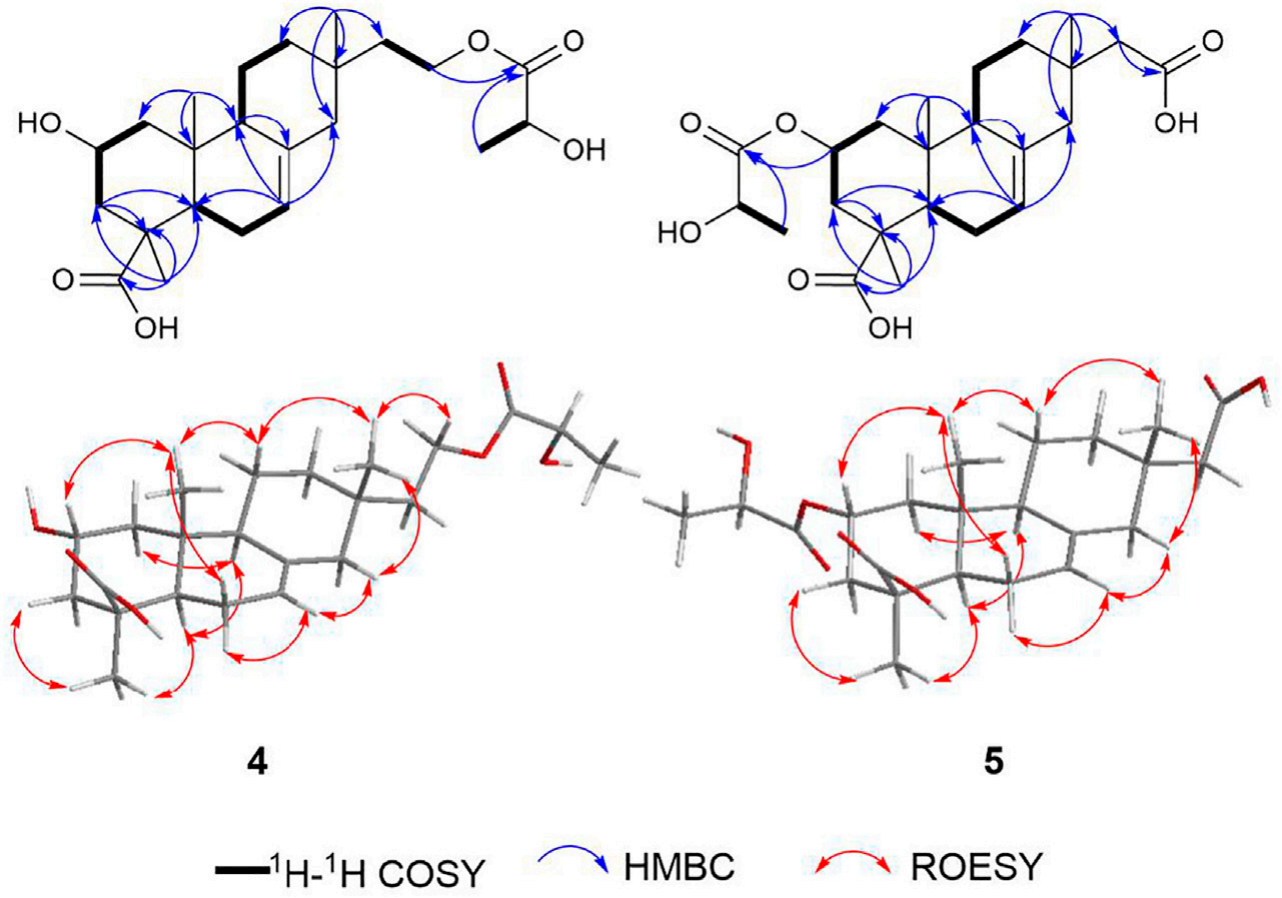
Key 2D NMR correlations of compound **4** and **5**.

**FIGURE 7:**
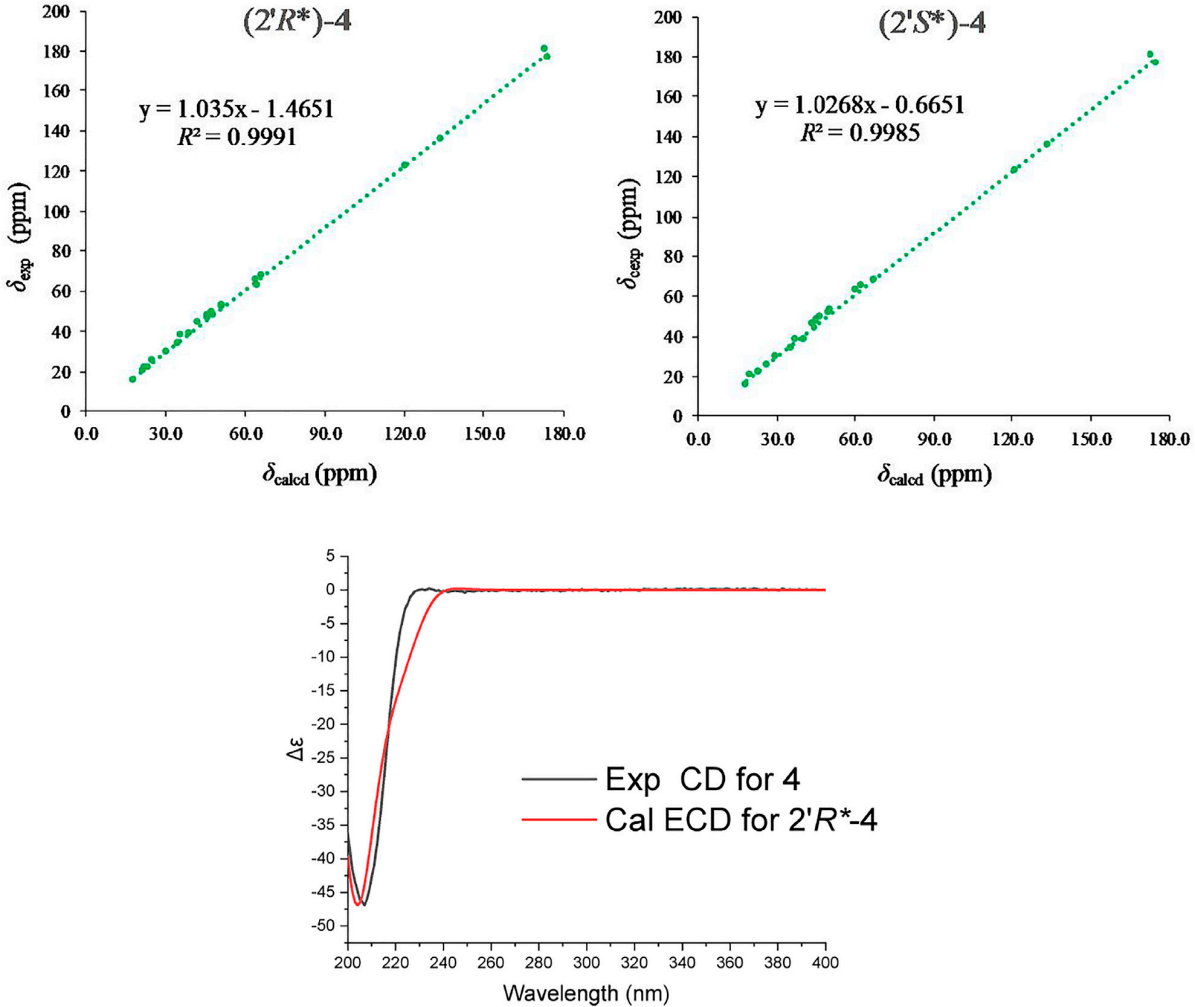
Regression analysis of the NMR calculations of the two possible configurations, (2′R*) and (2′S*)-**4**, and ECD calculation result of **4**.

Robustaditerpene D (**4**), white powder, gave a molecular formula C_23_H_36_O_6_, as established by HRESIMS data (*m/z* 431.24045 [M + Na]^+^). The 1D NMR data (Table 2) were closely related to those of **2**, except for three additional carbons at δ_c_ 176.4 (C-1′), δ_c_ 67.9 (C-2′), and δ_c_ 20.5 (C-3′), and the downfield-shifted carbon C-16 (δ_c_ 62.8 in **4**, δ_c_ 59.1 in **2**). In combination with comparison of molecular mass of **2** and **4**, the HMBC correlations from H-16 to C-1′ and H-3′ to C-1′ and the ^1^H–^1^H COSY correlations of H-2′/H-3′ (Figure 6) indicated that the three additional carbons (C-1′ C-2′, and C-3′) were a lactic acid esterified with the hydroxyl at C-16. Further analysis of 2D NMR data suggested other parts of **4** were the same as those of **2**. In accordance with the biogenetic pathway rules, the absolute configuration of **2** would be the same as the other parts of **4**, which indicated other of **4** to be the isopimarane skeleton. Therefore, **4** consisted of esterification of the hydroxyl at C-16 of **2** and a lactic acid. However, the *R/S* configuration of C-2′ can’t be determined by the ROESY signals. To further confirm the relative configuration of **4**, especially at C-2′, ^13^C NMR calculations of two possible conformers (2′R*)-**4** and (2′S*)-**4** were performed. As shown in Figure 7, the correlation coefficient (*R* ^2^), mean absolute deviation (MAE), and the largest deviations (Δ_max_) of the (2′R*)-**4** isomer [*R* ^2^ = 0.9991, MAE = 1.16 ppm, Δ_max_ = 3.2 ppm (C-19)] were better than (2′S*)-**4** isomer [*R* ^2^ = 0.9985, MAE = 1.58 ppm, Δ_max_ = 3.4 ppm (C-19)] (Supplementary Material), which suggested that the relative configuration of C-2′ is *R.* The absolute configuration of **4,** the *R/S* configuration of the lactic acid part, was determined by the comparison of calculated ECD and experimental CD spectra (Figure 7). Compound **4** was characterized as shown.

**FIGURE 8:**
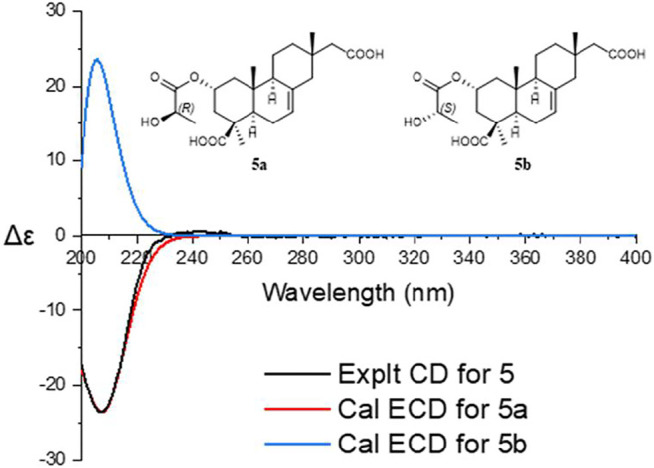
ECD calculation of compound **5**.

Robustaditerpene E (**5**) was isolated as a yellow amorphous solid, and its molecular formula C_23_H_34_O_7_ (*m/z* 445.21960 [M + Na]^+^), was established by HRESIMS. The 1D NMR data (Table 2) were similar to those of **3**, but three additional carbons at δ_c_ 176.0 (C-1′), δ_c_ 68.0 (C-2′), and δ_c_ 20.6 (C-3′) and downfield-shifted C-2 (δ_c_ 70.8 in **5**, δ_c_ 65.3 in **3**). According to the HMBC correlations from H-2 to C-1′ (δ_c_ 176.0) and H-3′ to C-1′, the ^1^H–^1^H COSY signals (Figure 6) of H-2′/H-3′, and the comparison of molecular mass of **3** and **5**, these additional carbons also belonged to a lactic acid esterified with the hydroxyl at C-2. Other parts of **5** were identical to those of **3** after the analysis of 2D NMR spectra. Thus, **5** consisted of esterification of the hydroxy at C-2 of **3** and a lactic acid. Biogenetically, other parts of **5** and **3** shared the same isopiamarane skeleton, and absolute configuration. The absolute configuration of **5**, in particular the *R/S* configuration of lactic acid part was based on the comparison of calculated ECD and experimental CD spectra (Figure 8). Thus, compound **5** was determined as shown.

### T and B Cell Proliferation Inhibitory Activity

The relatively abundant compounds **1**-**5** were evaluated for their inhibitory activity against cell proliferation of concanavalin A (Con A)-induced T lymphocytes and lipopolysaccharide (LPS)-induced B lymphocytes. As shown in Table 3, robustaditerpene C **3**) and E (**5**) showed the suppressive activity against the cell proliferation of LPS-induced B lymphocytes with IC_50_ value at 17.42 ± 1.57 *μ*M and Con A-induced T lymphocytes with IC_50_ value at 75.22 ± 6.10 *μ*M, respectively. These data indicate that compounds **3** and **5** have certain research value in immunosuppression.

**TABLE 3:** T and B cell proliferation inhibitory activity for compounds 3 and 5

Compound	ConA-induced T-cell proliferation	LPS-induced B-cell proliferation
	IC_50_ (*μ*M)	IC_50_ (*μ*M)
**3**		17.42 ± 1.57
**5**	75.22 ± 6.10	
CsA^a^	0.05 ± 0.002	0.37 ± 0.01

a Positive control.

Isopimarane diterpenes are biosynthetically formed from the conversion of geranylgeranyl pyrophosphate into (+)-copalyl cation and then in turn into sandaracopimaraneyl cation (Dewick, 2009). Interestingly, these diterpenes are generally obtained from plants and fungi, which showed diverse bioactivity (Reveglia et al., 2018). Until now, isopimarane diterpenes with immunosuppressive activity were first reported in 2020 (Chen et al., 2020). This research provides several isopimarane scaffolds with potential immunosuppressive activity and enriched structure diversity. These compounds give more choices to develop new agents with inhibition on the steps of immune response. And these isopimarane scaffold compounds will be modified by organic synthesis to improve the immunosuppressive activity.

## Conclusion

Herein, five new isopimarane diterpenes, which possess 19-nor-isopimarane skeleton or isopimarane skeleton, were obtained from the endophytic fungus *Ilyonectria robusta*. According to 1D and 2D NMR analysis, X-ray single crystal diffraction analysis, ^13^C NMR calculation, and ECD calculation, the absolute configuration of these compounds was confirmed. Biological evaluation for immunosuppressive activity indicated that compound **3** and **5** displayed some activity against cell proliferation of B lymphocytes and T lymphocytes, respectively. Among these compounds, single crystal of compound **1** was obtained.

## Data Availability

The original contributions presented in the study are included in the article/Supplementary Material, further inquiries can be directed to the corresponding authors.
