# Supplementary material for: Immunosuppressive Isopimarane Diterpenes From Cultures of the Endophytic Fungus Ilyonectria robusta
Source: Front Pharmacol. 2022 Jan 10;12:766441. doi: 10.3389/fphar.2021.766441 (PMC8802225; doi:10.3389/fphar.2021.766441)
Supplement: Supplementary file 1 [file DataSheet2.docx]

Supplementary Material

Immunosuppressive isopimarane diterpenes from Cultures of the endophytic fungus *Ilyoncetria robusta*

Ke Ye^1^, Xian Zhang^1^, Xiao Lv^1^, Pan-Pan Wei^1^, Zheng-Hui Li^1^, Hong-Lian AI^1*^, Da-Ke Zhao^2*^, and Ji-Kai Liu^1*^

^1^South Central University for Nationalities, Wuhan, Peoples' republic of China

^2^Yunnan University, Yunnan, Peoples' republic of China

*** Correspondence:**Hong-Lian Ai
[aihonglian@mail.scuec.edu.cn](mailto:aihonglian@mail.scuec.edu.cn)

Da-Ke Zhao

[Zhaodk2012@ynu.edu.cn](mailto:Zhaodk2012@ynu.edu.cn)
Ji-Kai Liu
[liujikai@mail.scuec.edu.cn](mailto:liujikai@mail.scuec.edu.cn)

**Keywords:** Immunosuppressive activity; endophytic fungus; natural products; isopimarne diterpenes; ECD calculation.

Contents

[1. NMR spectra and MS for compound 1-5 2](#_Toc80886733)

[1.1 NMR spectra and MS for compound 1 2](#_Toc80886734)

[1.2 NMR spectra and MS for compound 2 7](#_Toc80886735)

[1.3 NMR spectra and MS for compound 3 11](#_Toc80886736)

[1.4 NMR spectra and MS for compound 4 15](#_Toc80886737)

[1.5 NMR spectra and MS for compound 5 19](#_Toc80886738)

[2. Standard orientation of the optimized conformers 23](#_Toc80886739)

# NMR spectra and MS for compound 1-5

## NMR spectra and MS for compound 1


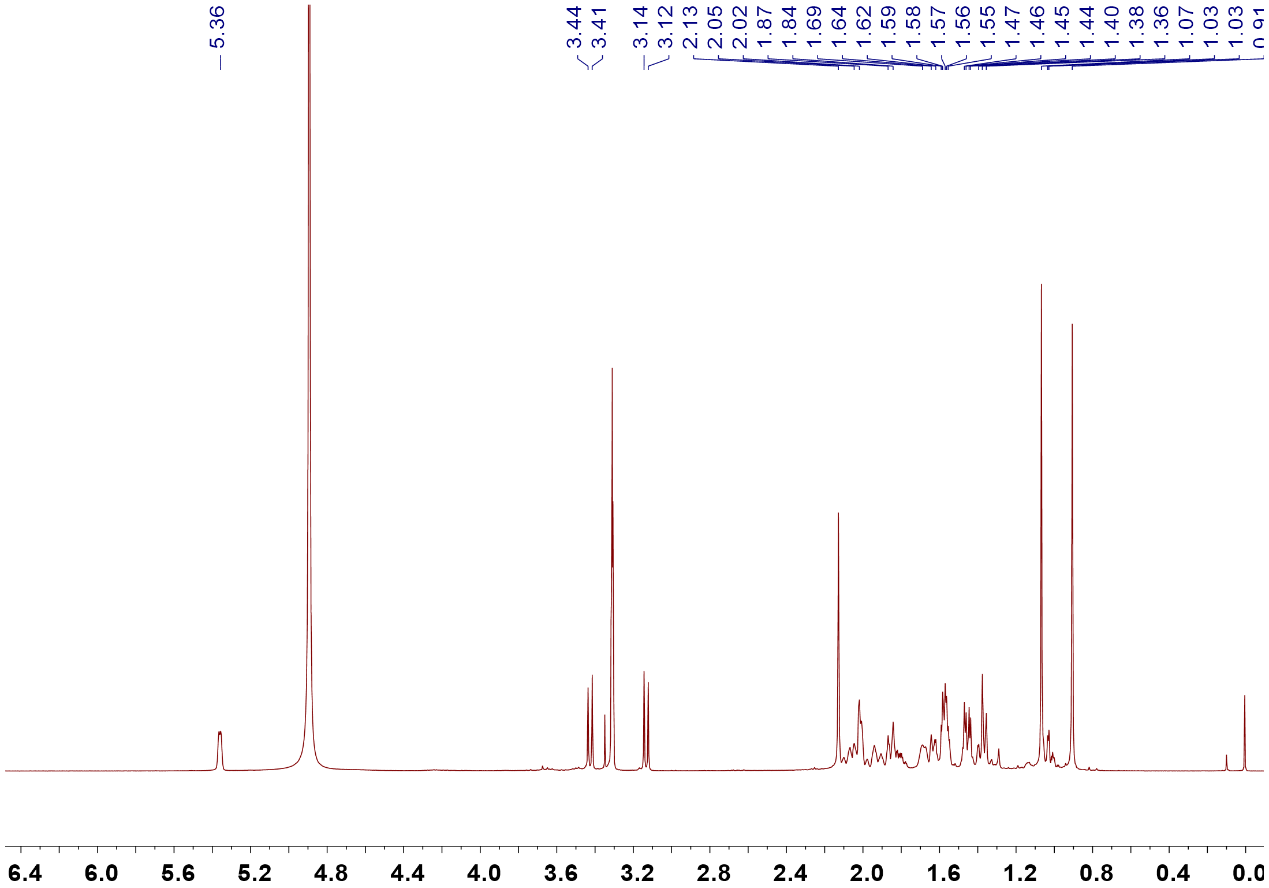


**Supplementary Figure 1.**^1^H NMR spectrum of compound **1** (Methanol-*d*_4_, 500 MHz)


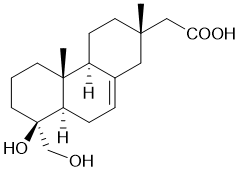

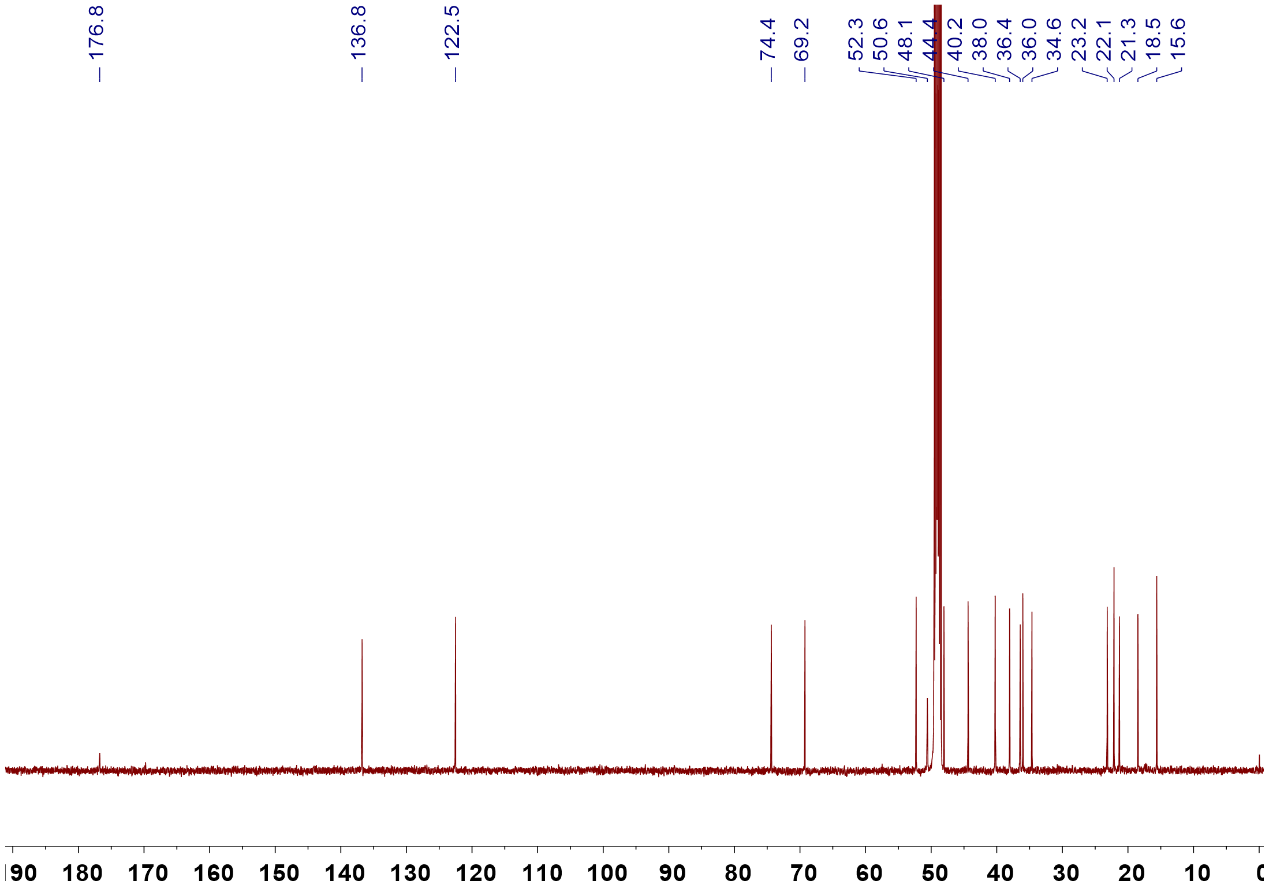


**Supplementary Figure 2.** ^13^C NMR spectrum of compound **1** (Methanol-*d*_4_, 125 MHz)


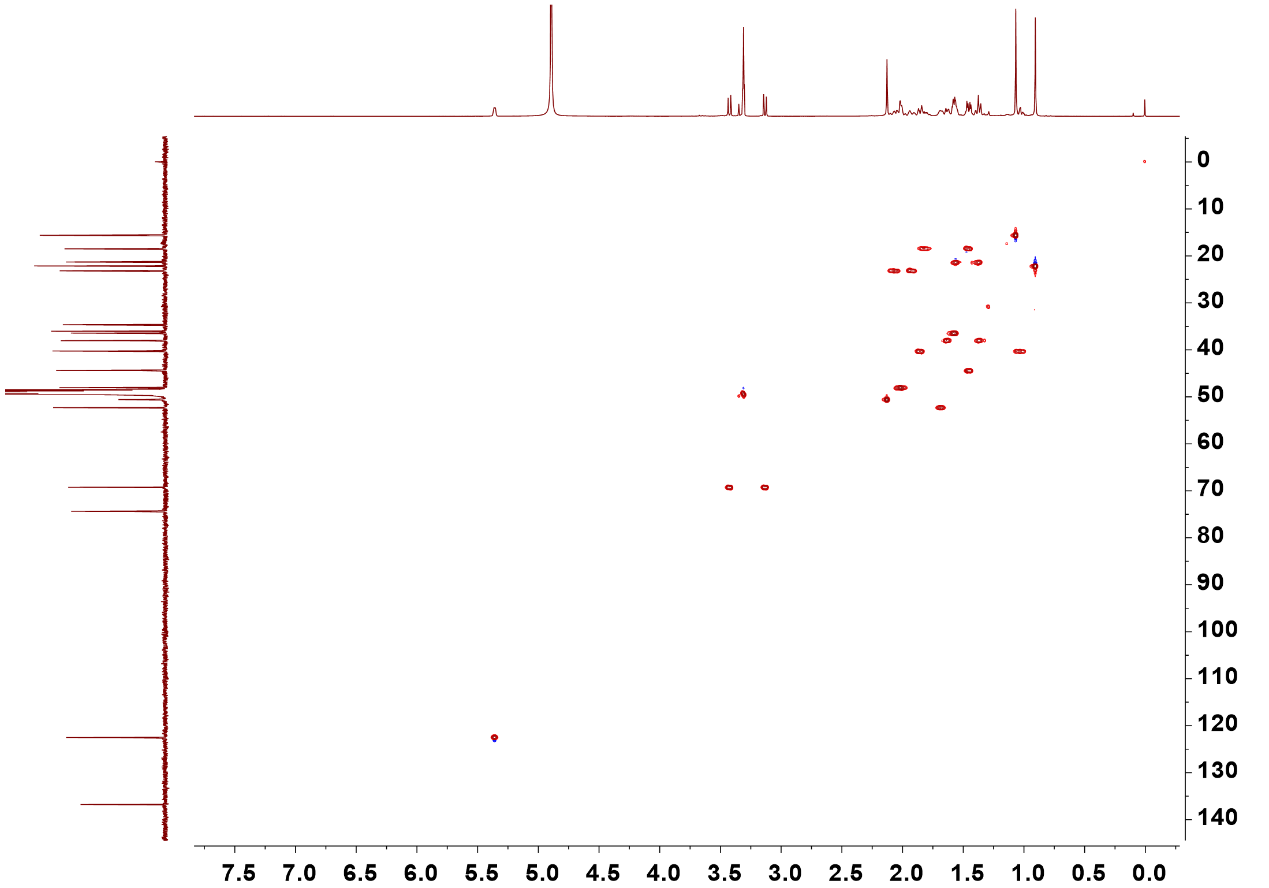


**Supplementary Figure 3.** HSQC spectrum of compound **1** (Methanol-*d*_4_)

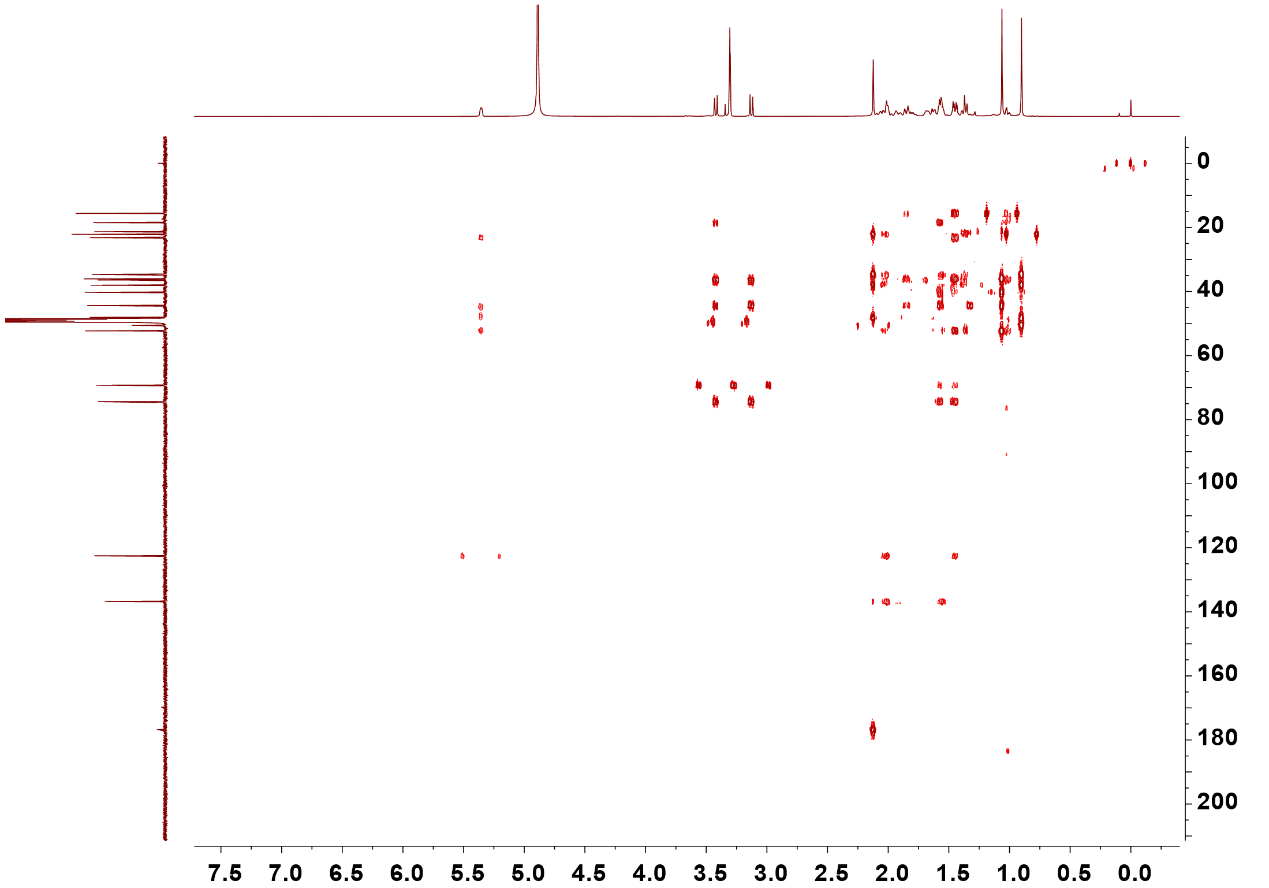


**Supplementary Figure 4.** HMBC spectrum of compound **1** (Methanol-*d*_4_)


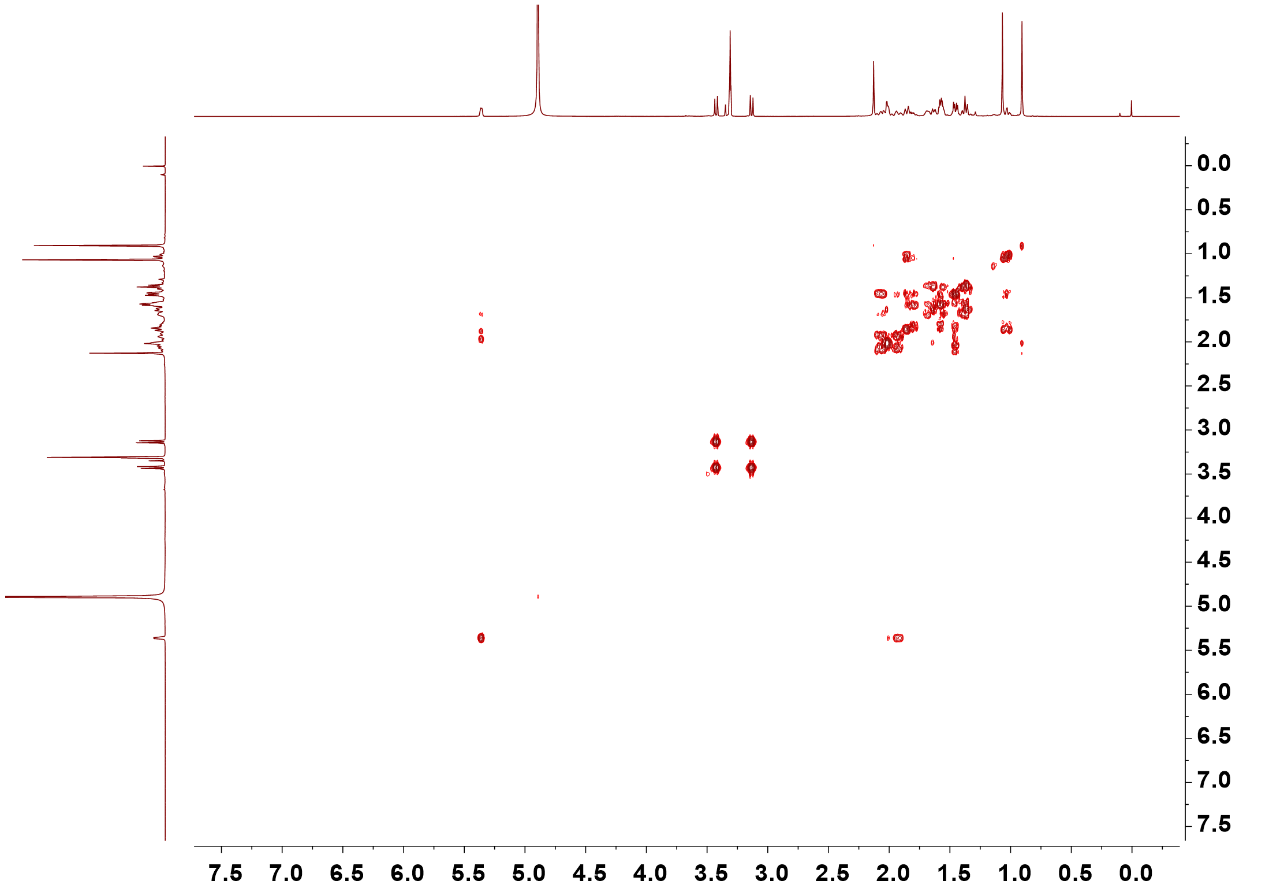


**Supplementary Figure 5.** ^1^H-^1^H COSY spectrum of compound **1** (Methanol-*d*_4_)

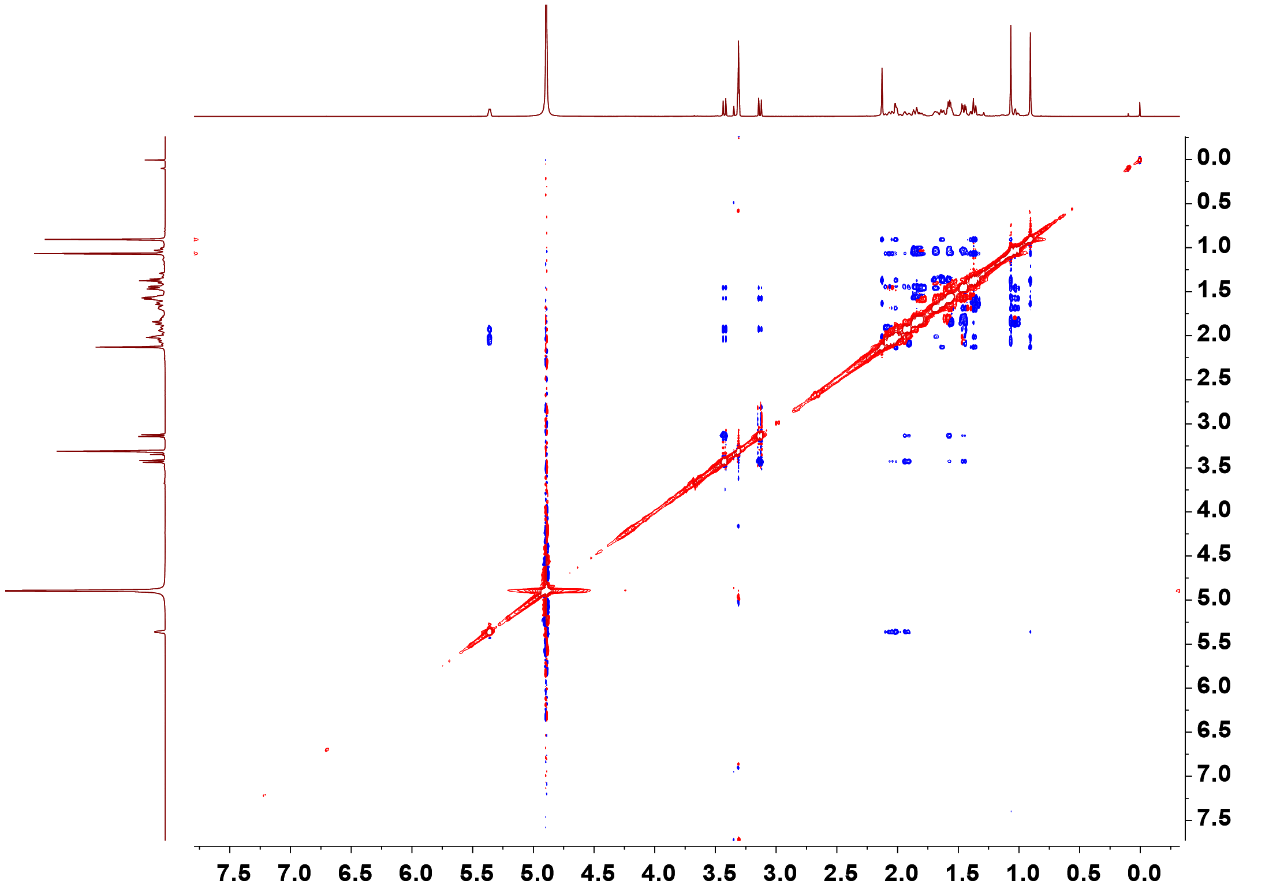


**Supplementary Figure 6.** ROESY spectrum of compound **1** (Methanol-*d*_4_)


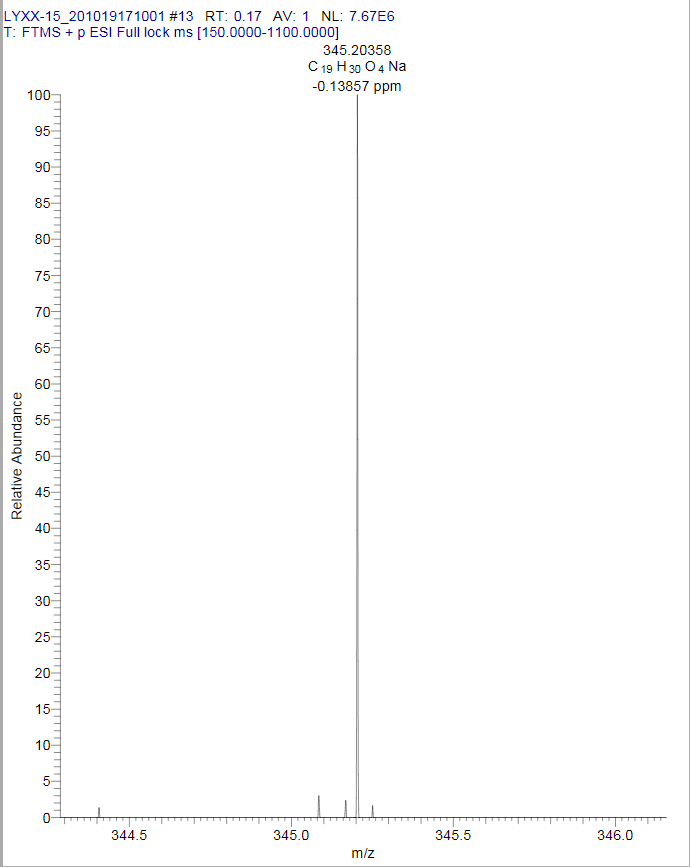


**Supplementary Figure 7.** HRESIMS spectrum of compound **1**

## NMR spectra and MS for compound 2


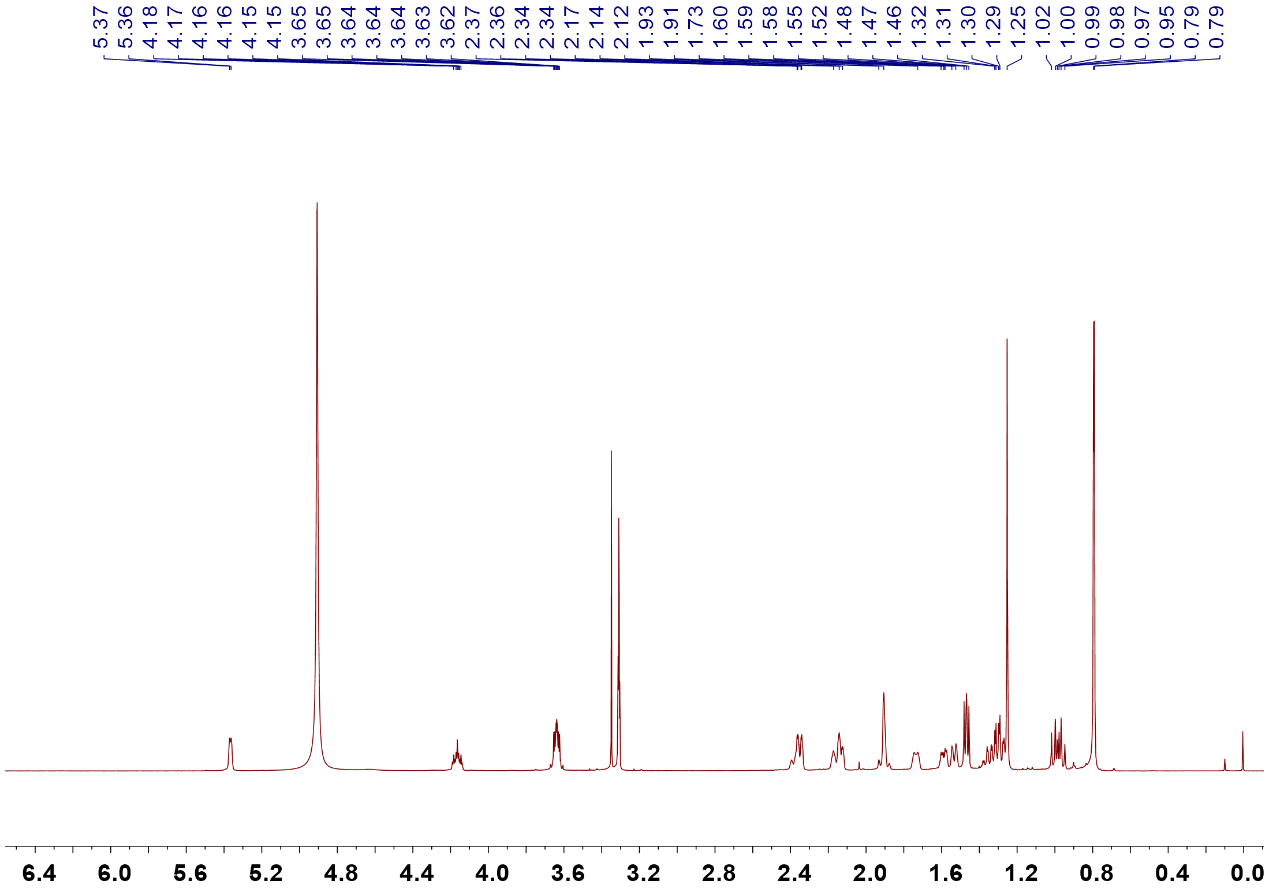


**Supplementary Figure 8.**^1^H NMR spectrum of compound **2** (Methanol-*d*_4_, 500 MHz)


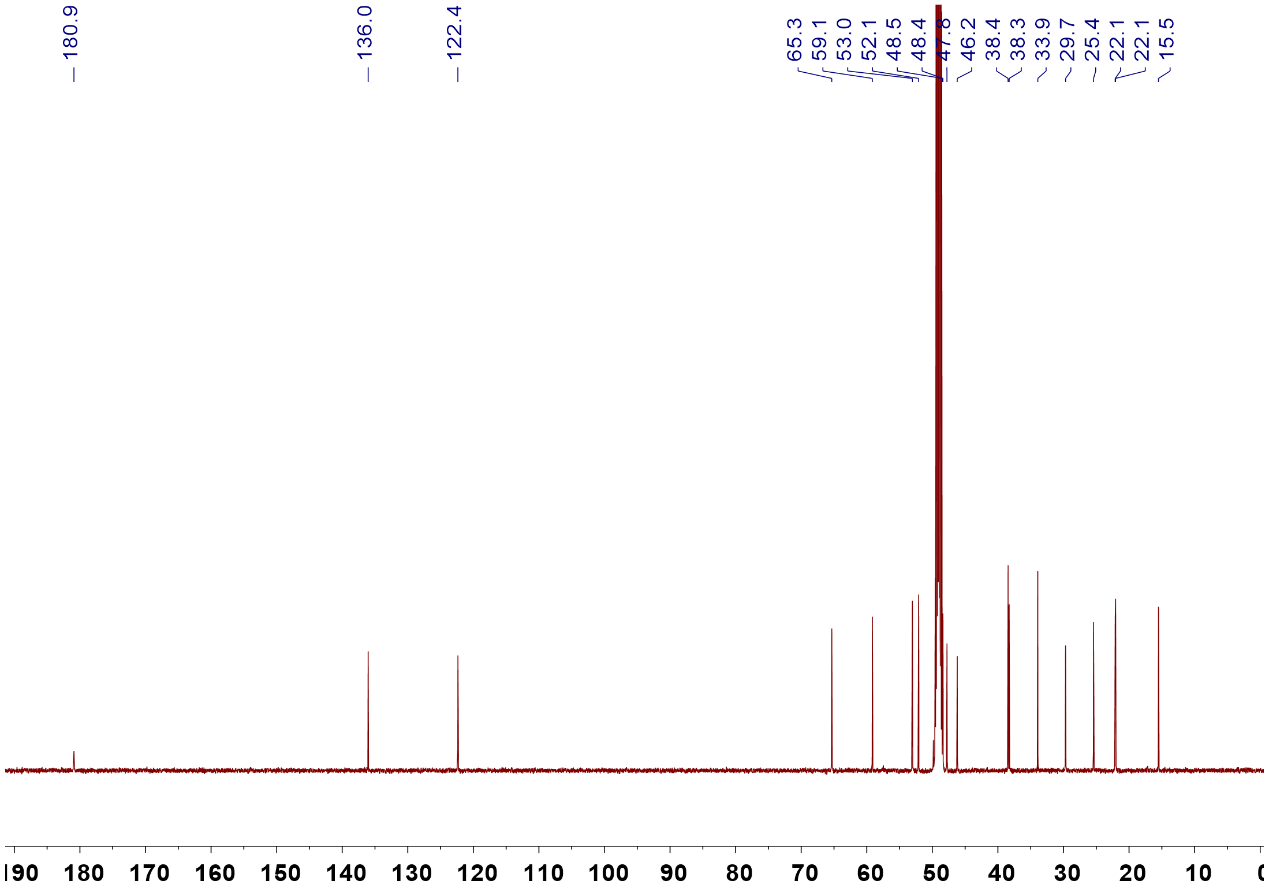


**Supplementary Figure 9.** ^13^C NMR spectrum of compound **2** (Methanol-*d*_4_, 125 MHz)

**
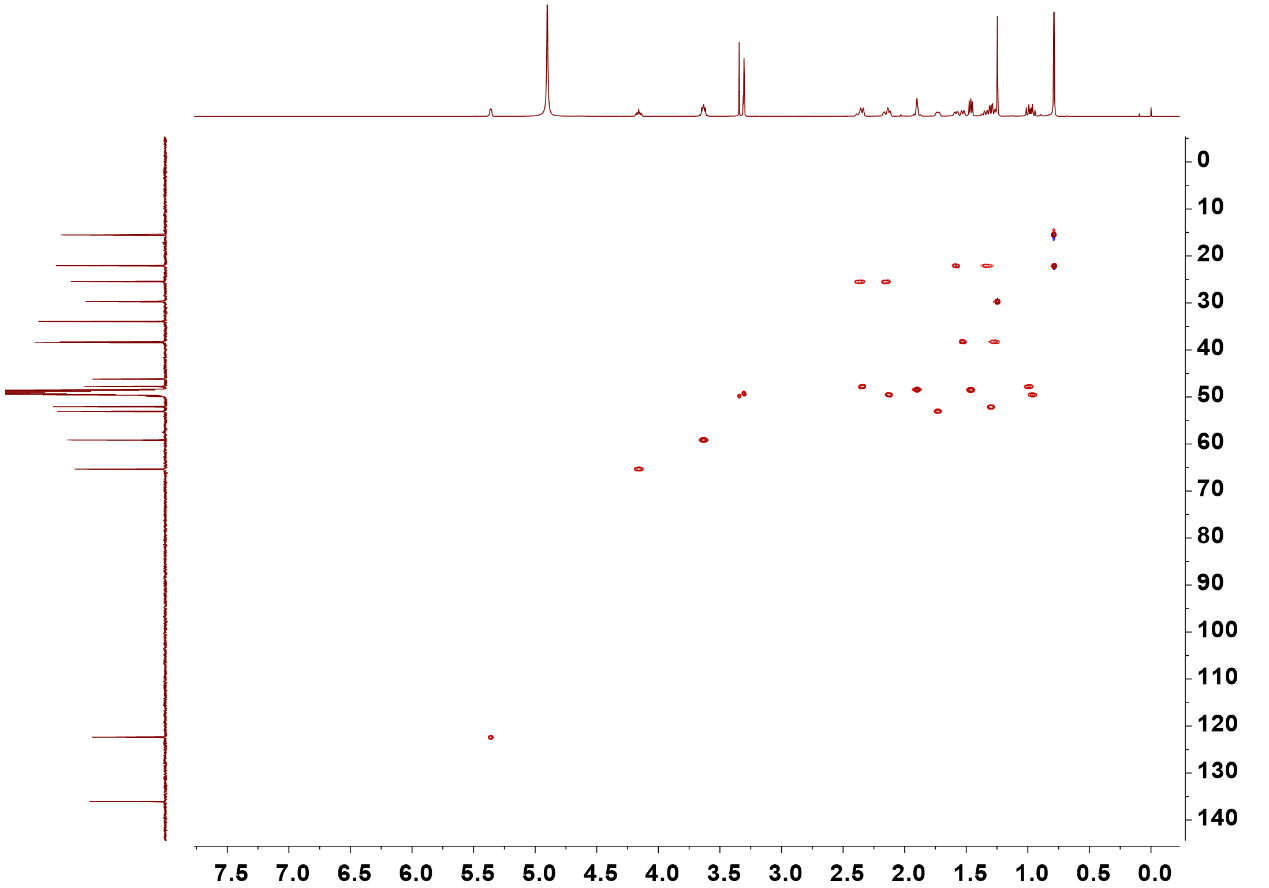
 Supplementary Figure 10.** HSQC spectrum of compound **2** (Methanol-*d*_4_)

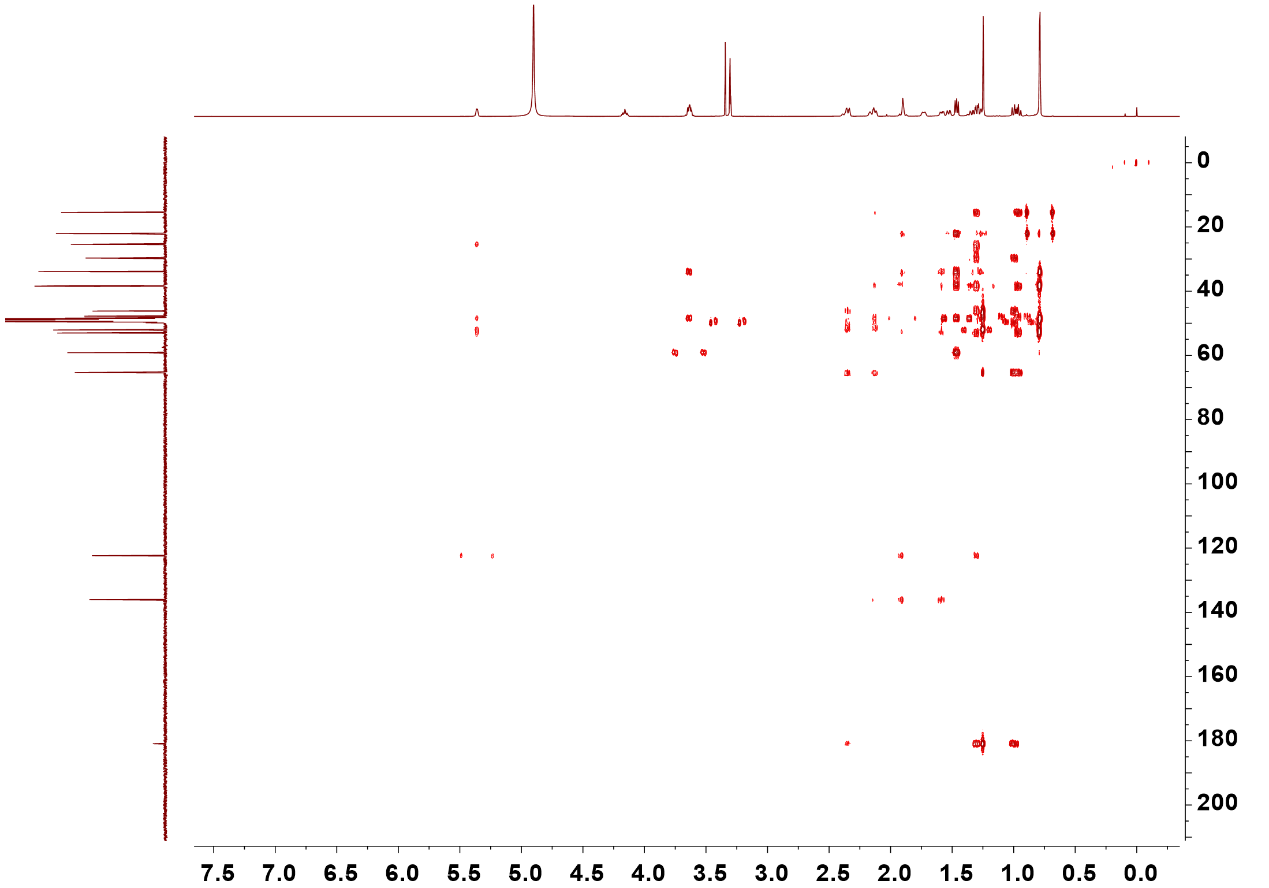


**Supplementary Figure 11.** HMBC spectrum of compound **2** (Methanol-*d*_4_)

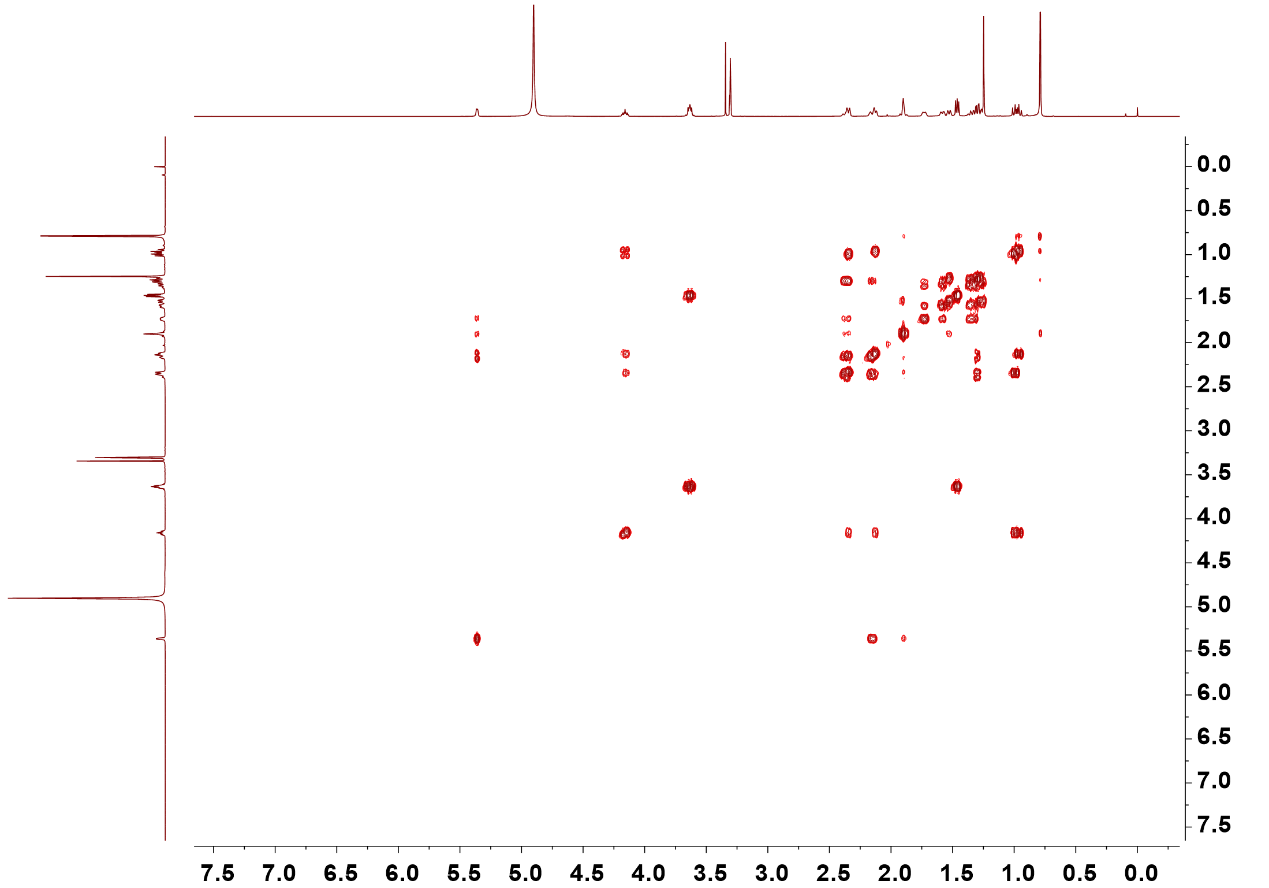


**Supplementary Figure 12.** ^1^H-^1^H COSY spectrum of compound **2** (Methanol-*d*_4_)

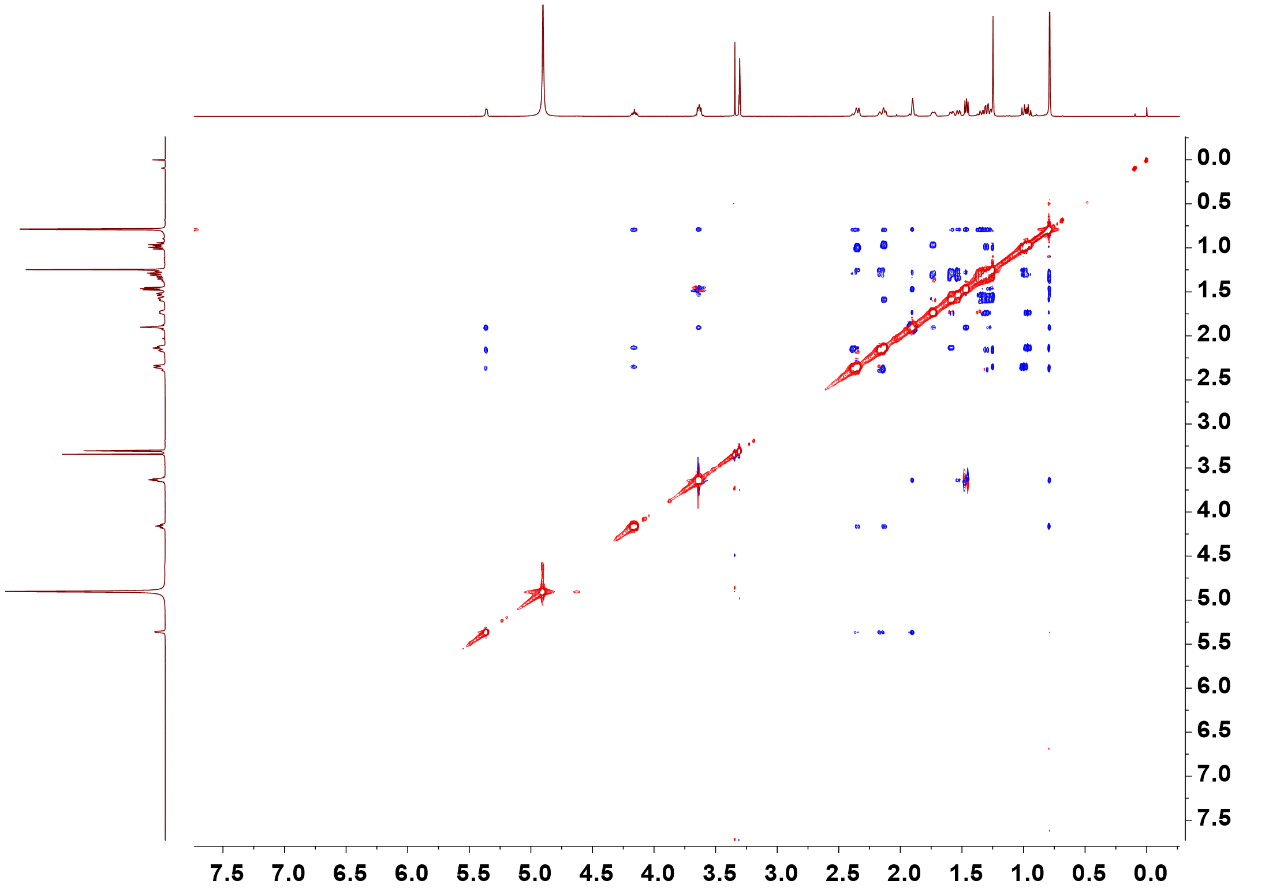


**Supplementary Figure 13.** ROESY spectrum of compound **2** (Methanol-*d*_4_)


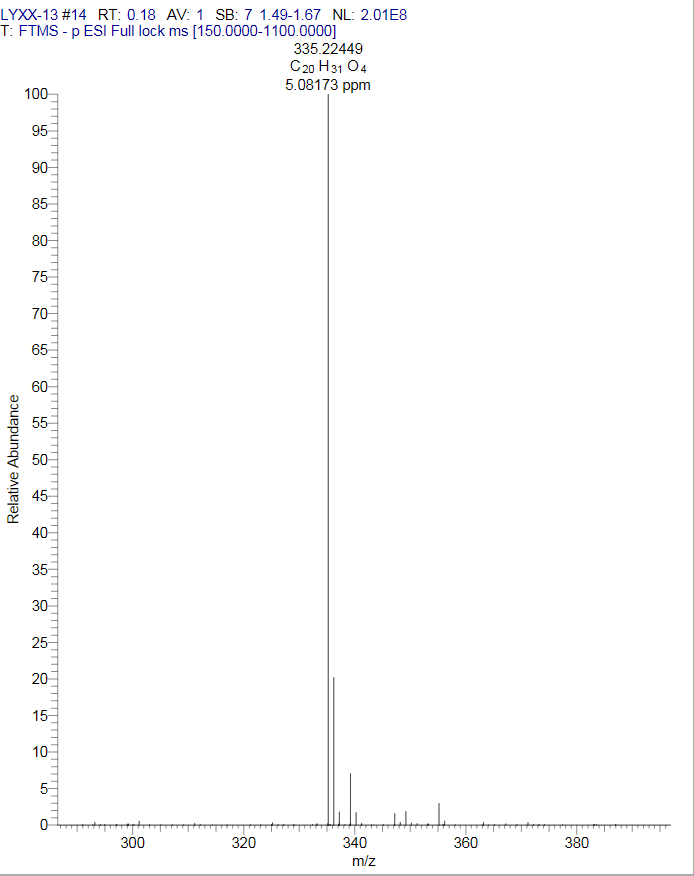


**Supplementary Figure 14.** HRESIMS spectrum of compound **2**

## NMR spectra and MS for compound 3


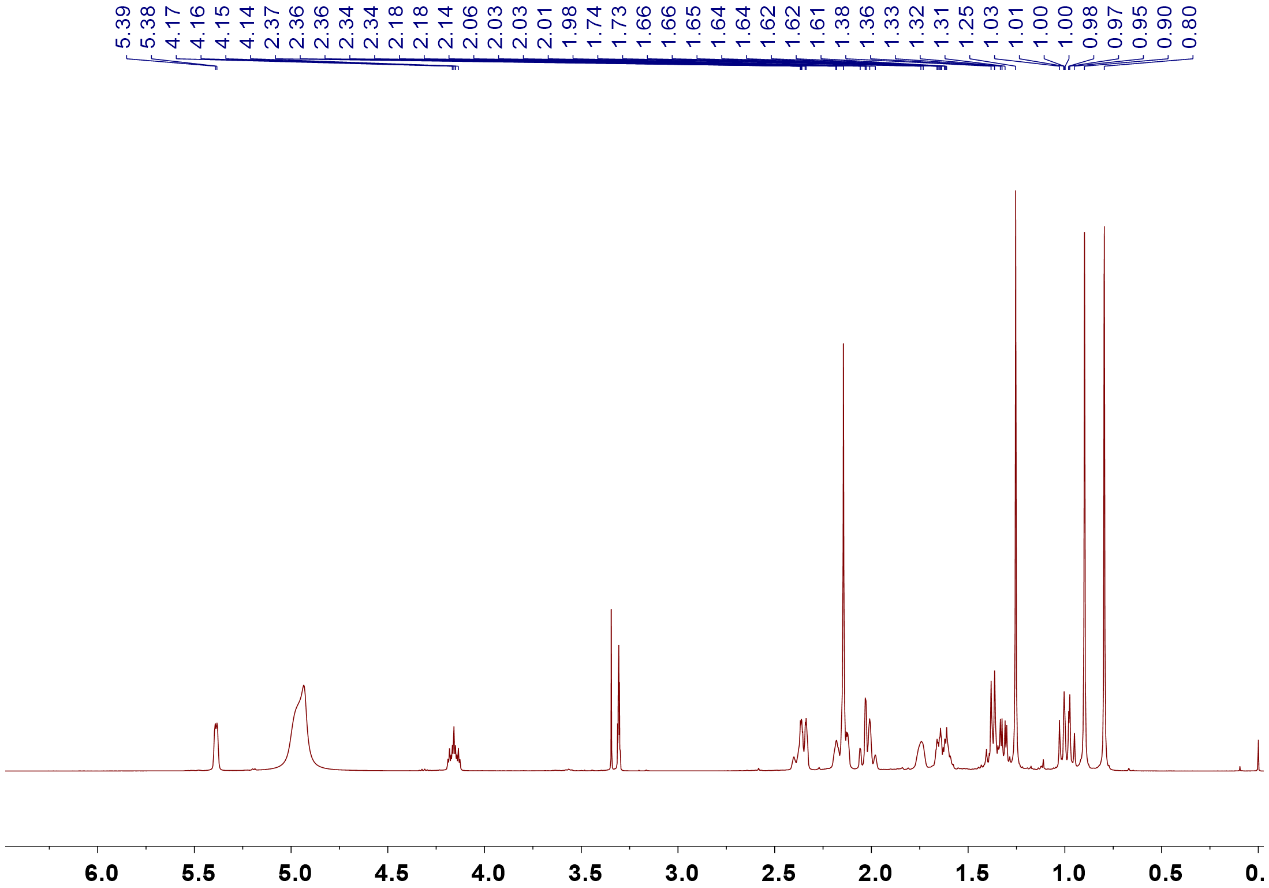


**Supplementary Figure 15.** ^1^H NMR spectrum of compound **3** (Methanol-*d*_4_, 600 MHz)


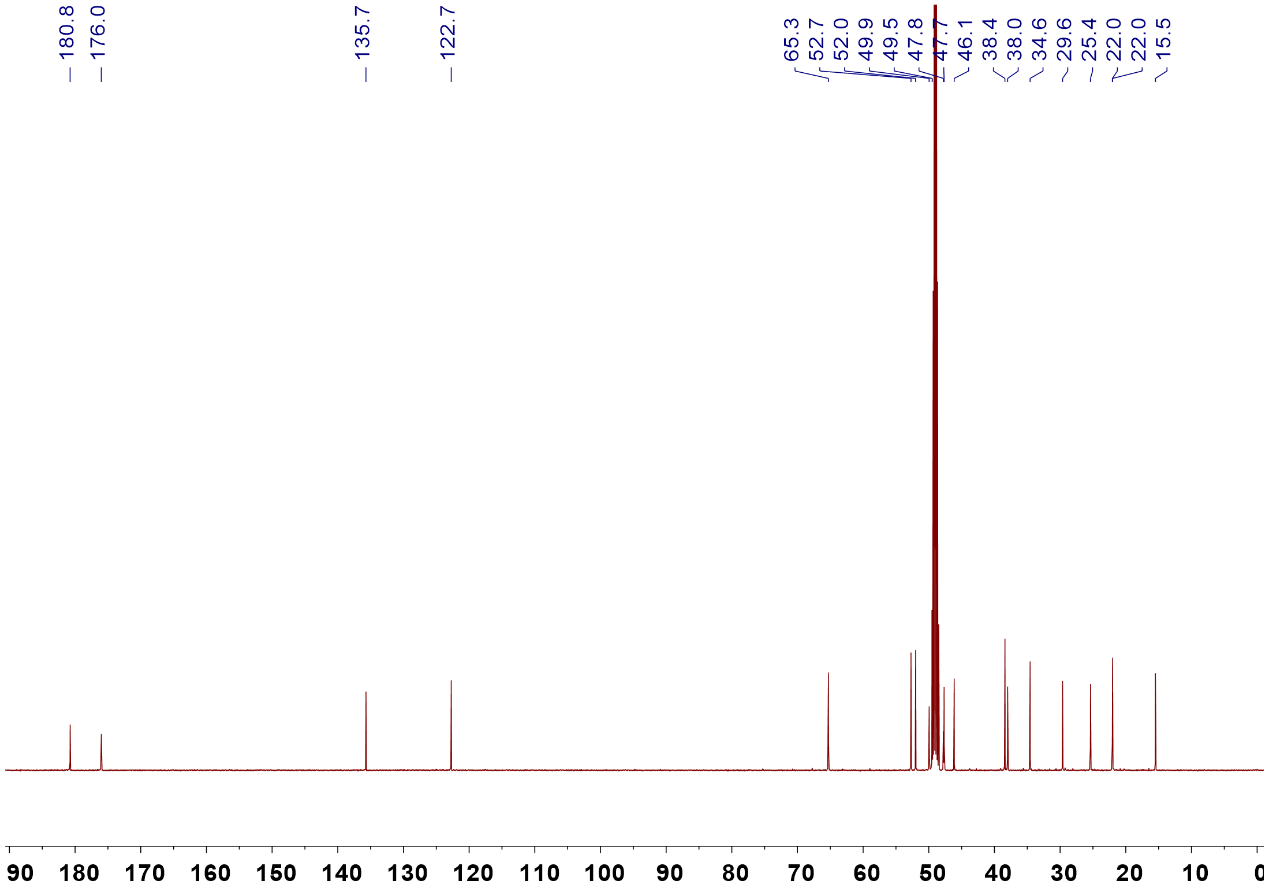


**Supplementary Figure 16.** ^13^C NMR spectrum of compound **3** (Methanol-*d*_4_, 150 MHz)


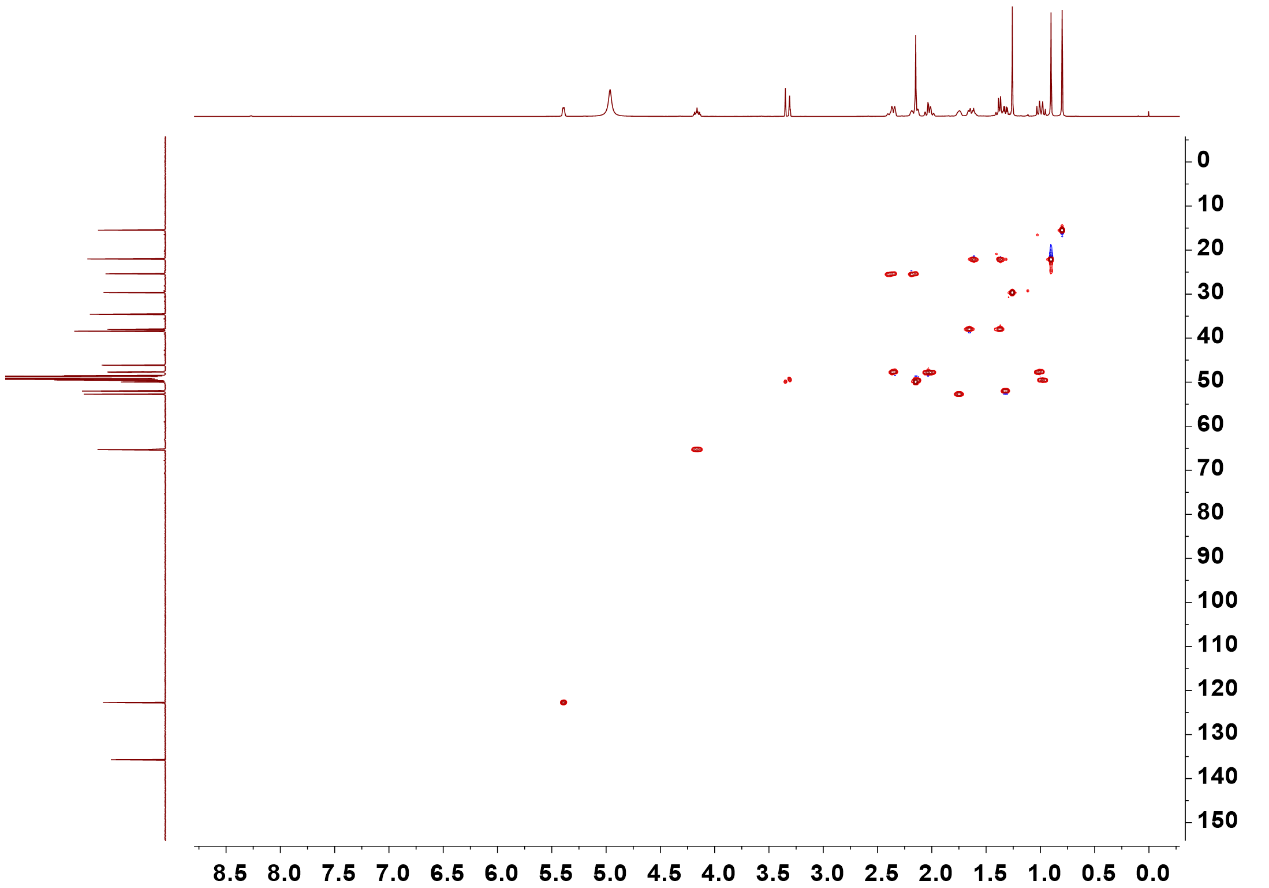


**Supplementary Figure 17.** HSQC spectrum of compound **3** (Methanol-*d*_4_)

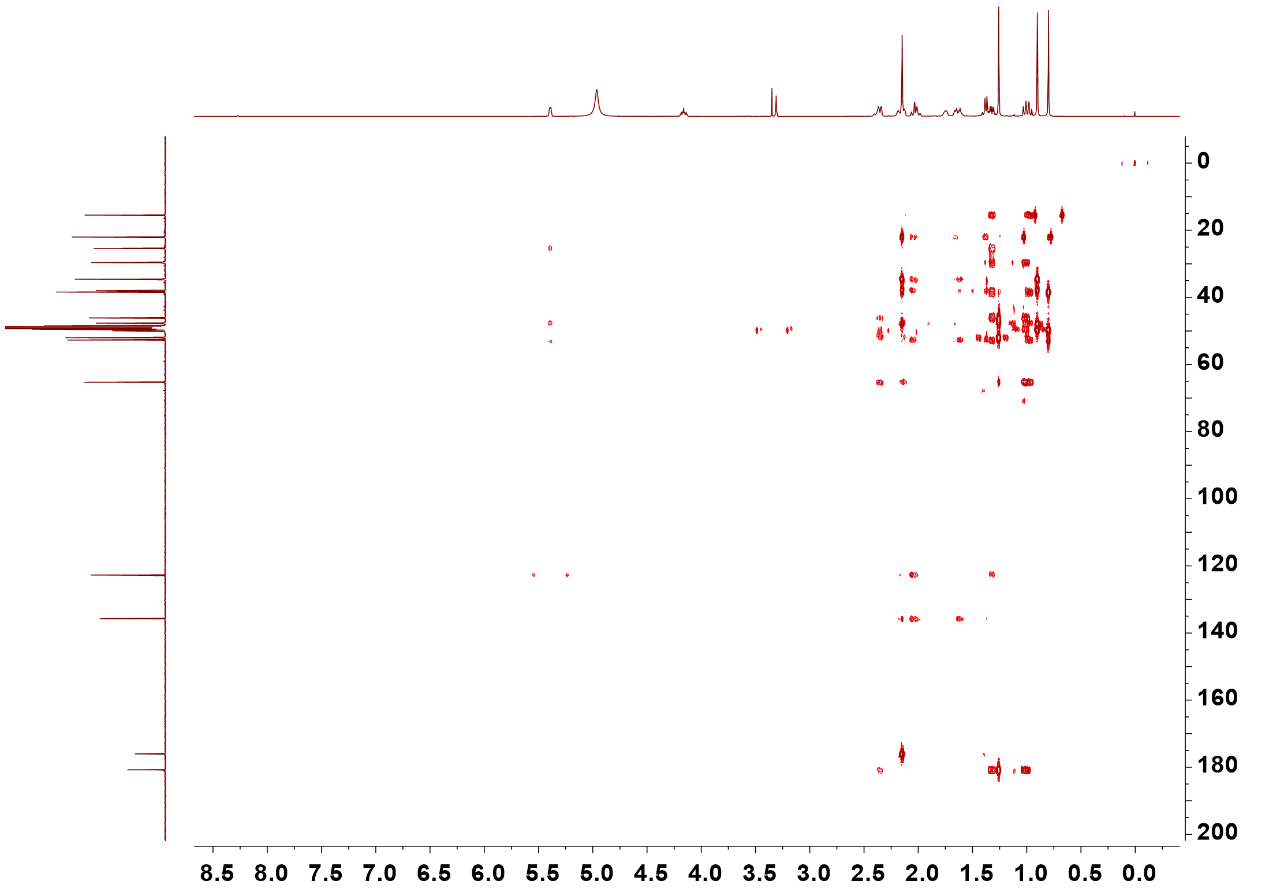


**Supplementary Figure 18.** HMBC spectrum of compound **3** (Methanol-*d*_4_)


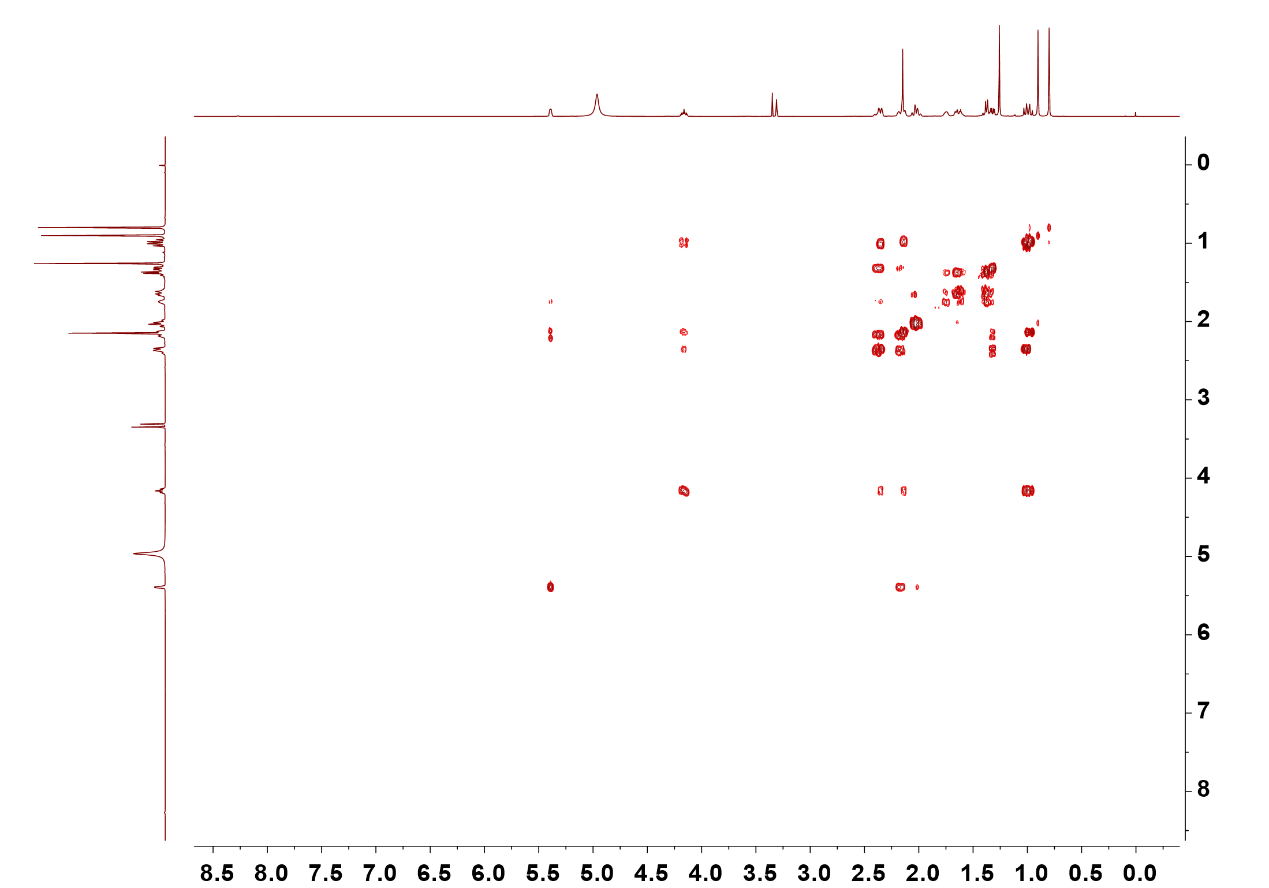


**Supplementary Figure 19.** ^1^H-^1^H COSY spectrum of compound **3** (Methanol-*d*_4_)

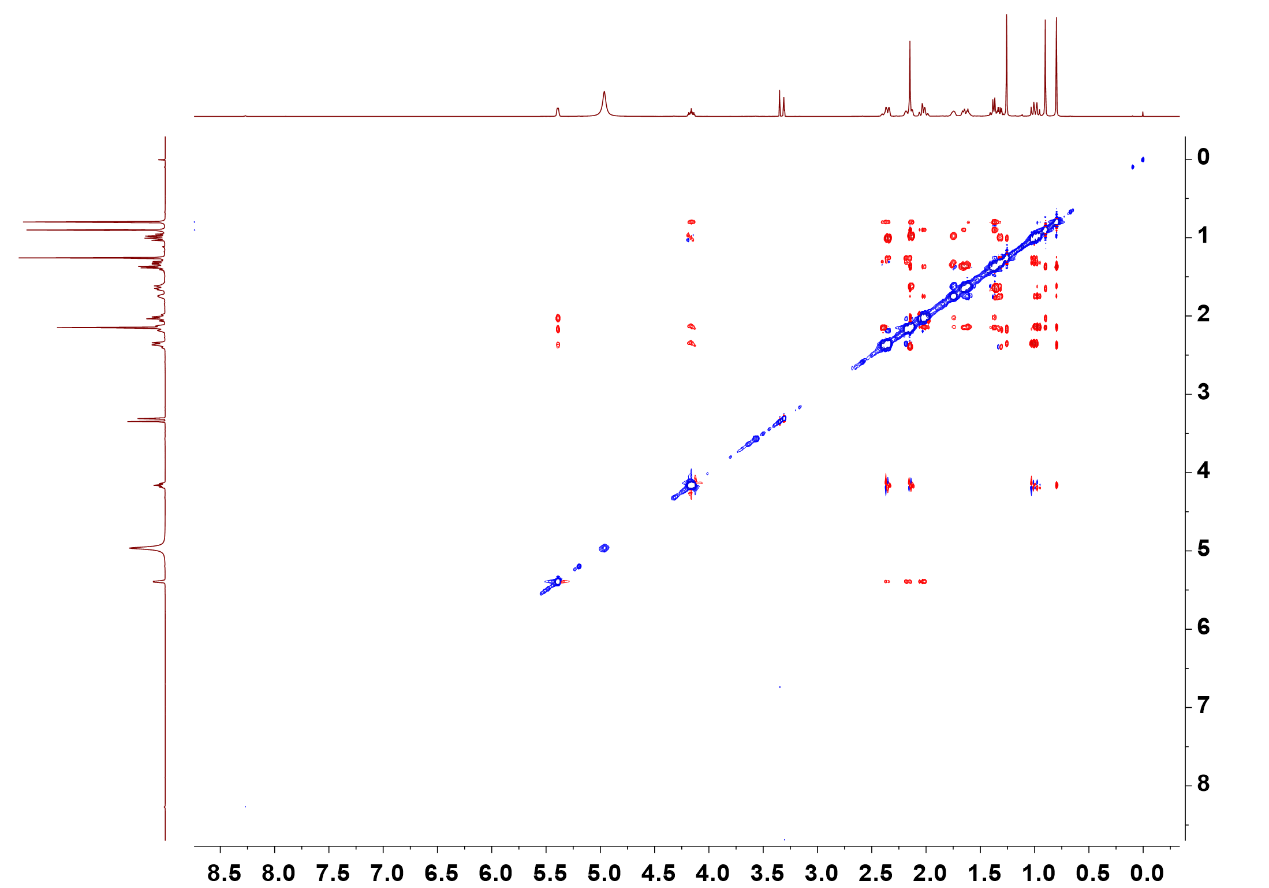


**Supplementary Figure 20.** ROESY spectrum of compound **3** (Methanol-*d*_4_)


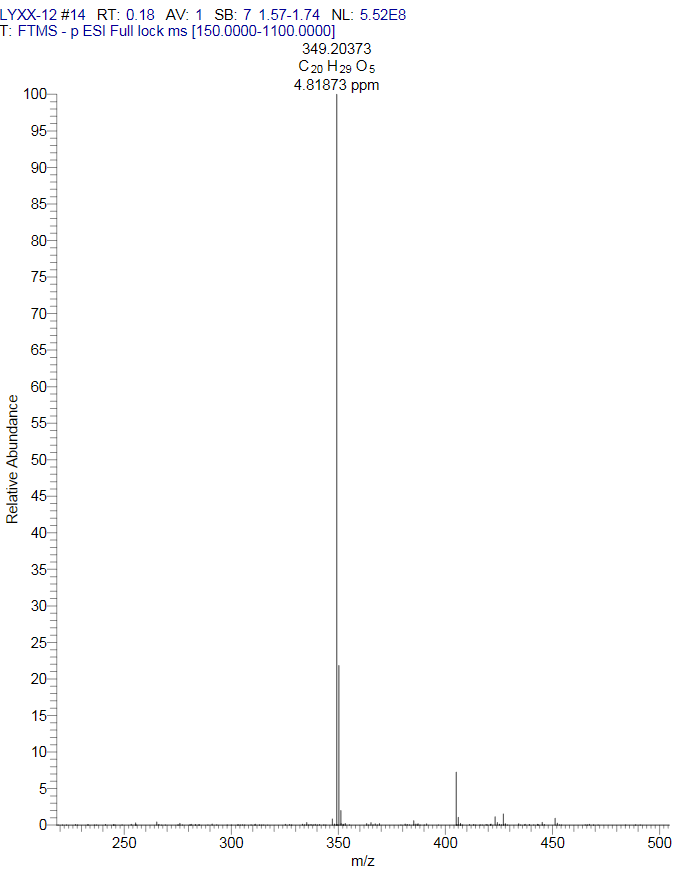


**Supplementary Figure 21.** HRESIMS spectrum of compound **3**

## NMR spectra and MS for compound 4


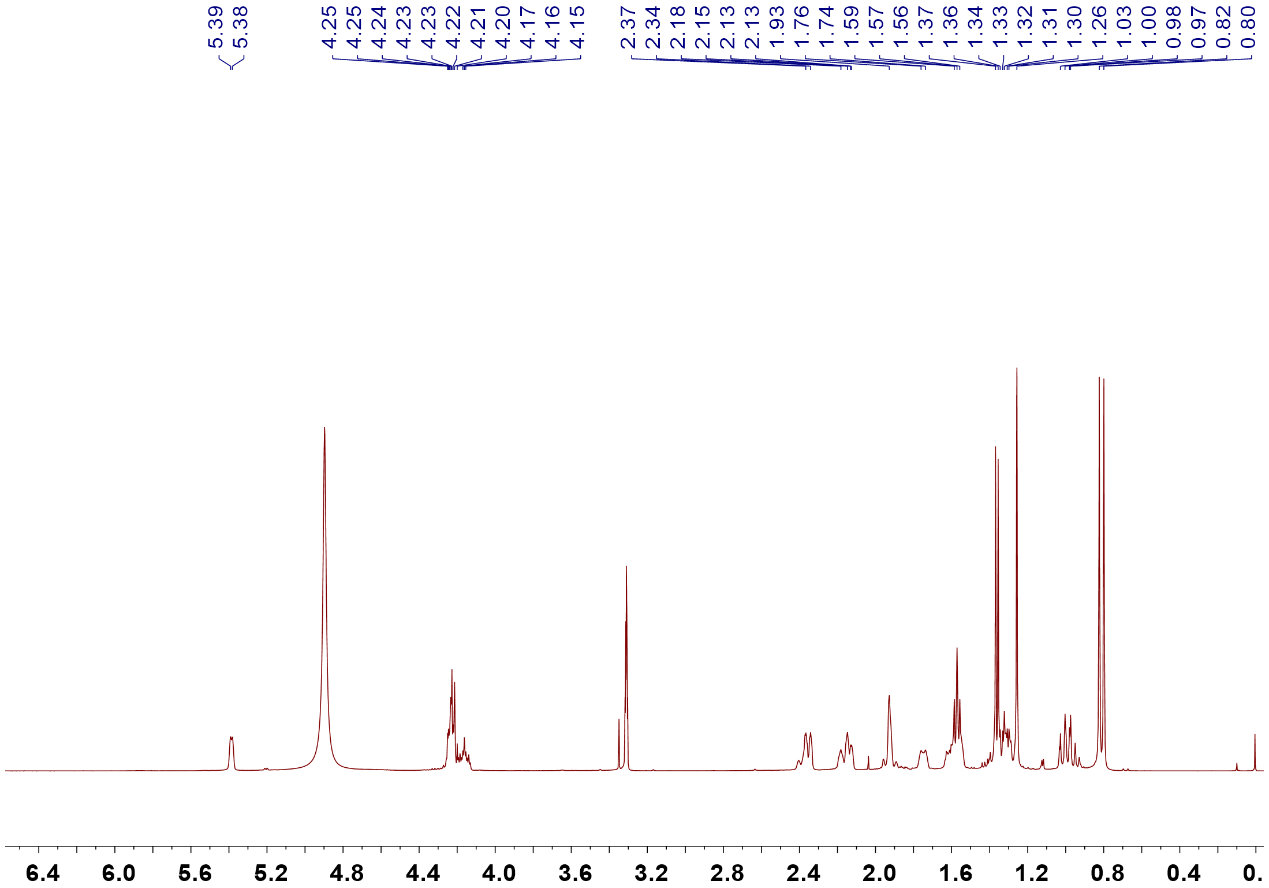


**Supplementary Figure 22.** ^1^H NMR spectrum of compound **4** (Methanol-*d*_4_, 500 MHz)


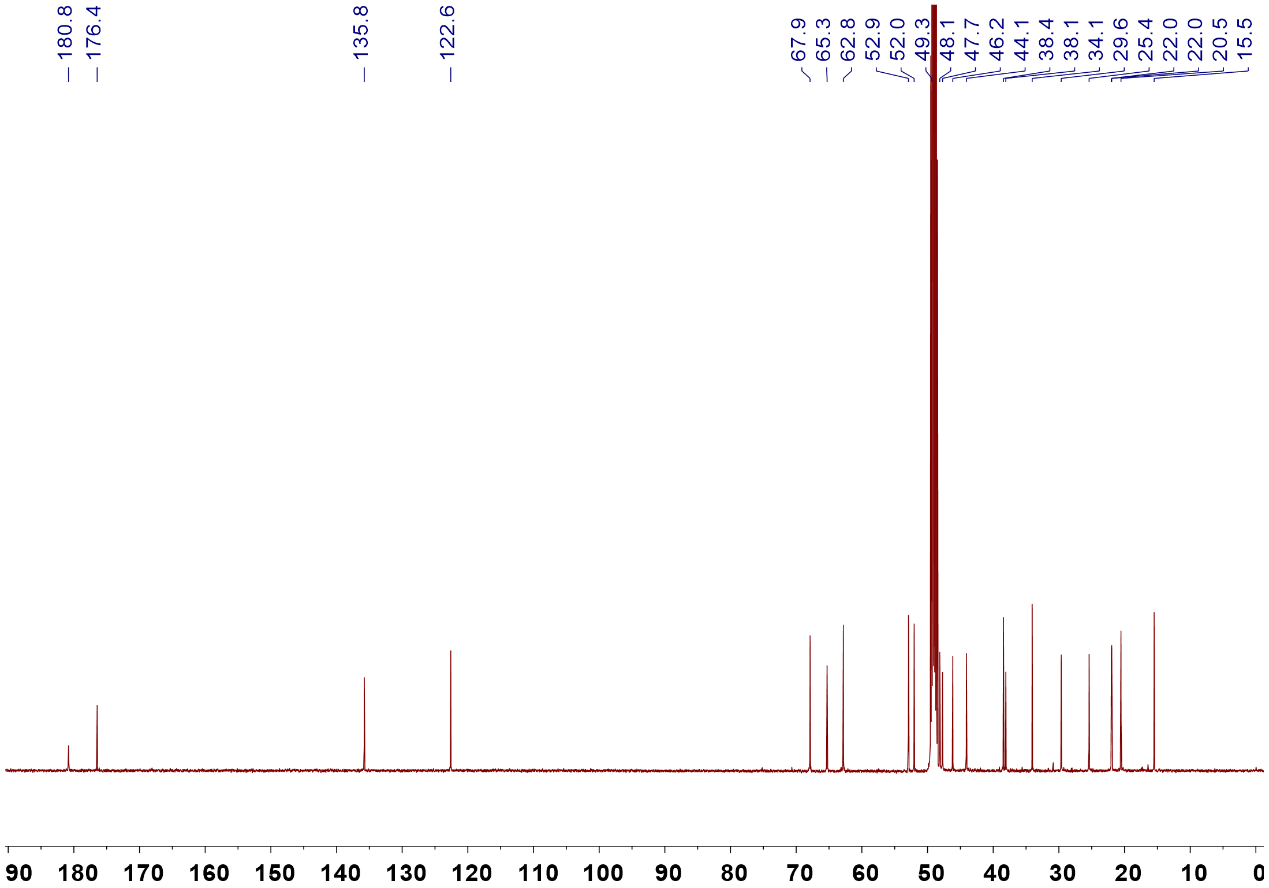


**Supplementary Figure 23.** ^13^C NMR spectrum of compound **4** (Methanol-*d*_4_, 125 MHz)


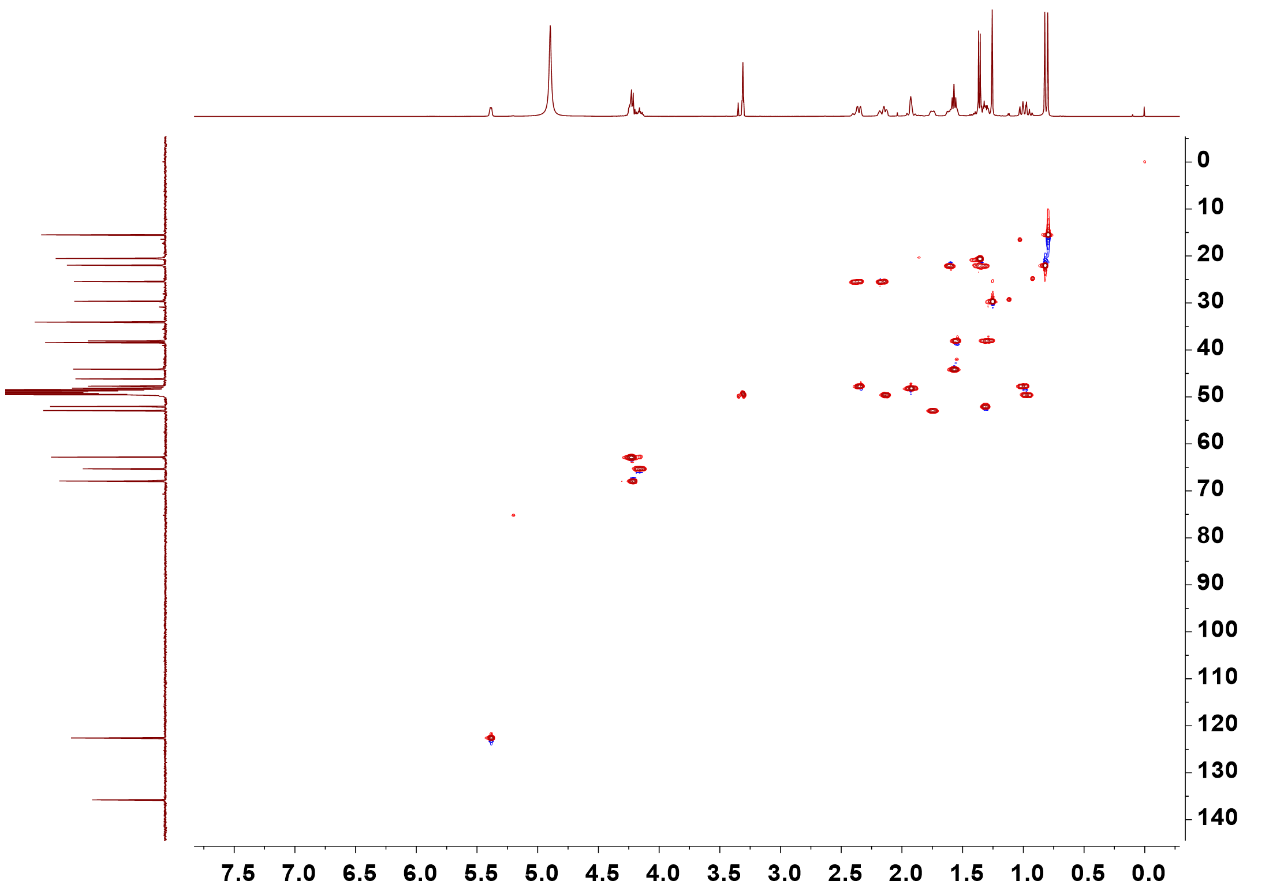


**Supplementary Figure 24.** HSQC spectrum of compound **4** (Methanol-*d*_4_)

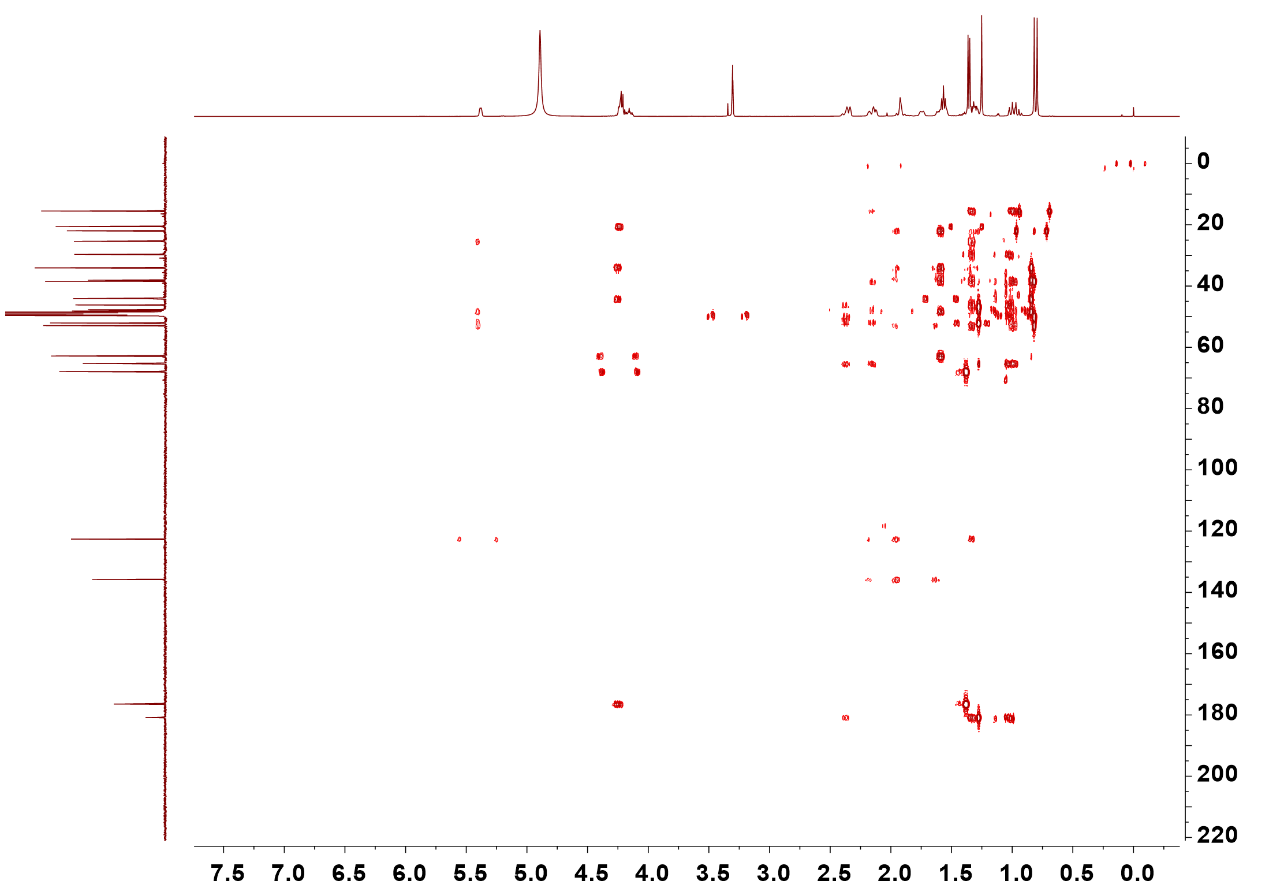


**Supplementary Figure 25.** HMBC spectrum of compound **4** (Methanol-*d*_4_)

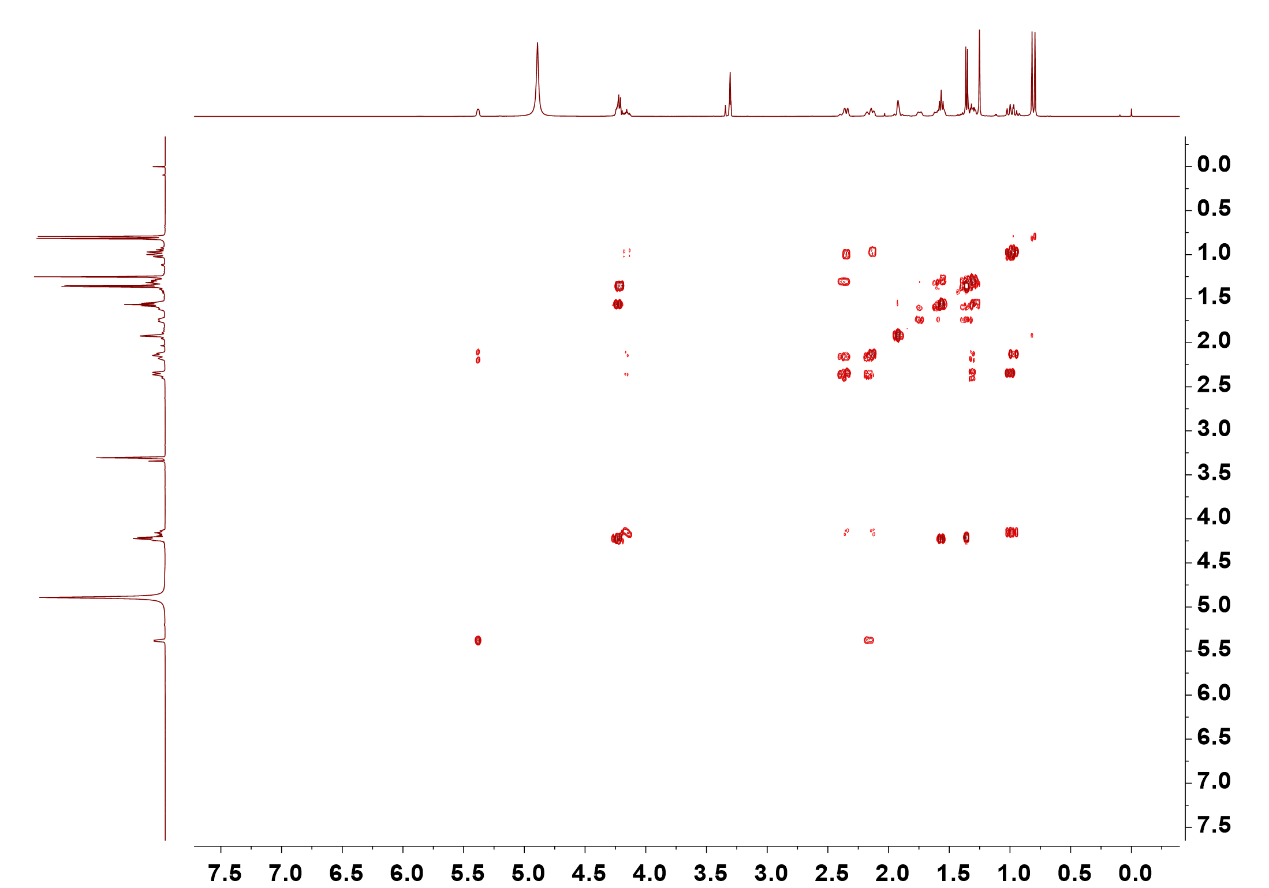


**Supplementary Figure 26.** ^1^H-^1^H COSY spectrum of compound **4** (Methanol-*d*_4_)

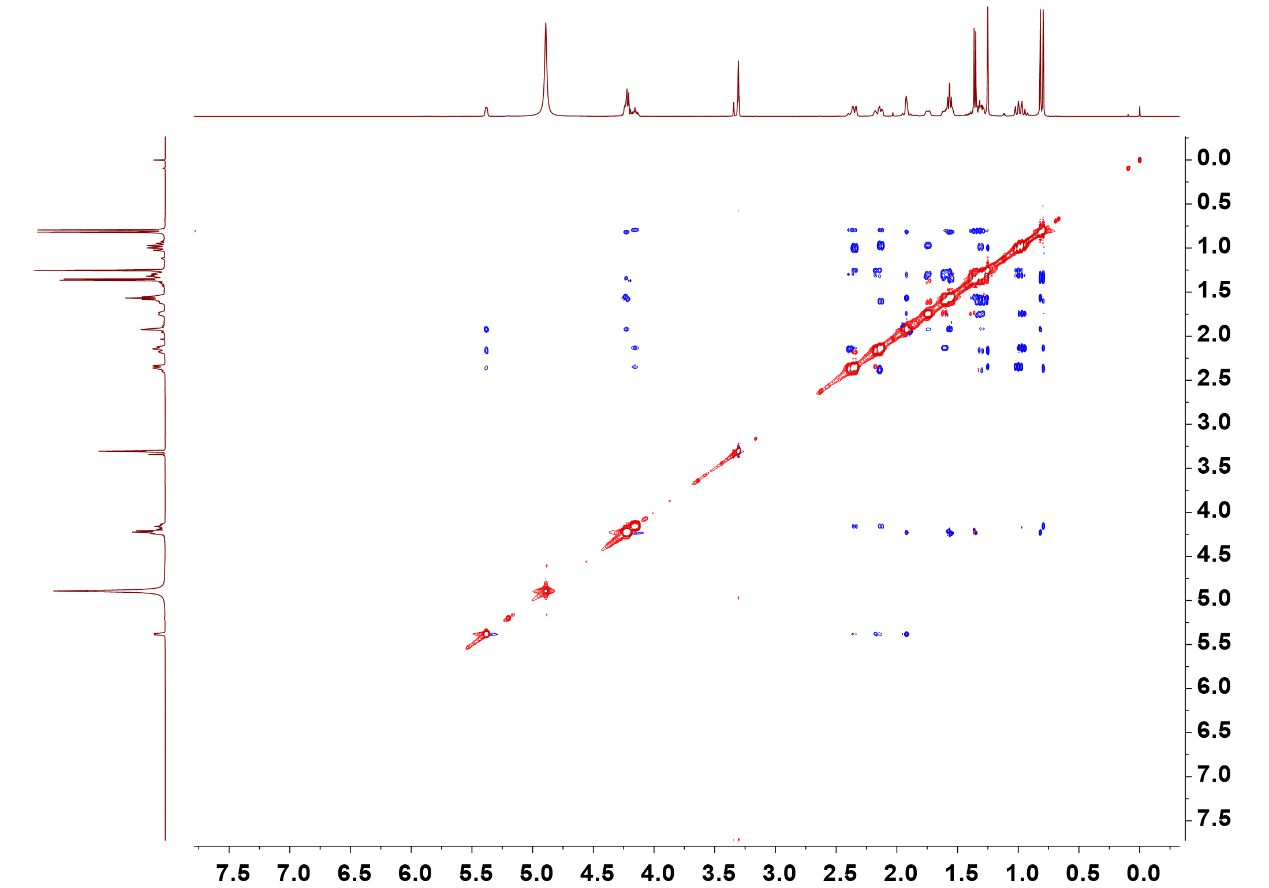


**Supplementary Figure 27.** ROESY spectrum of compound **4** (Methanol-*d*_4_)


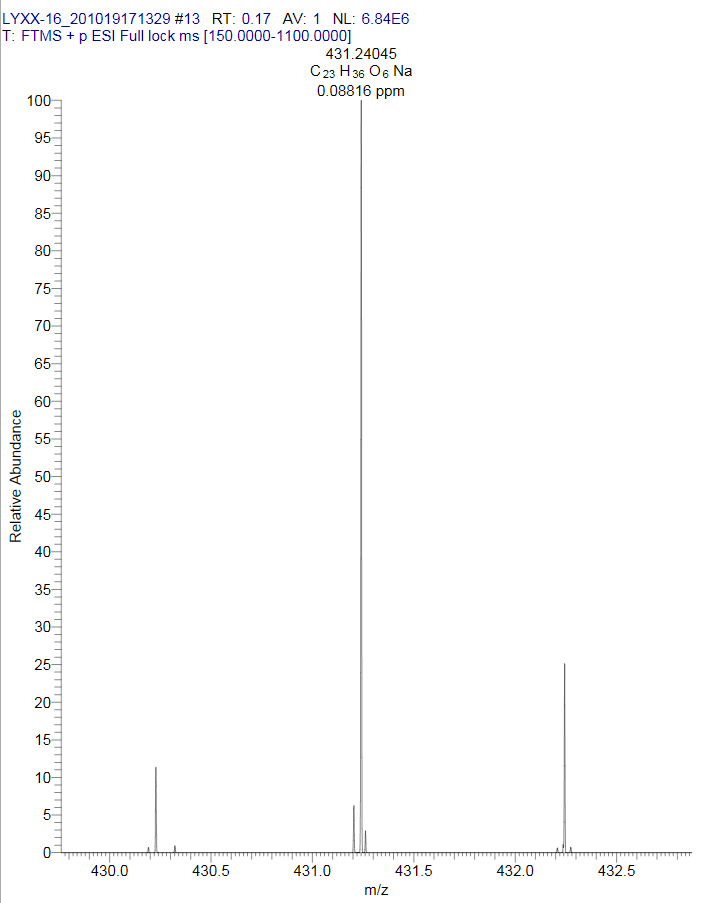


**Supplementary Figure28.** HRESIMS spectrum of compound **4**

## NMR spectra and MS for compound 5


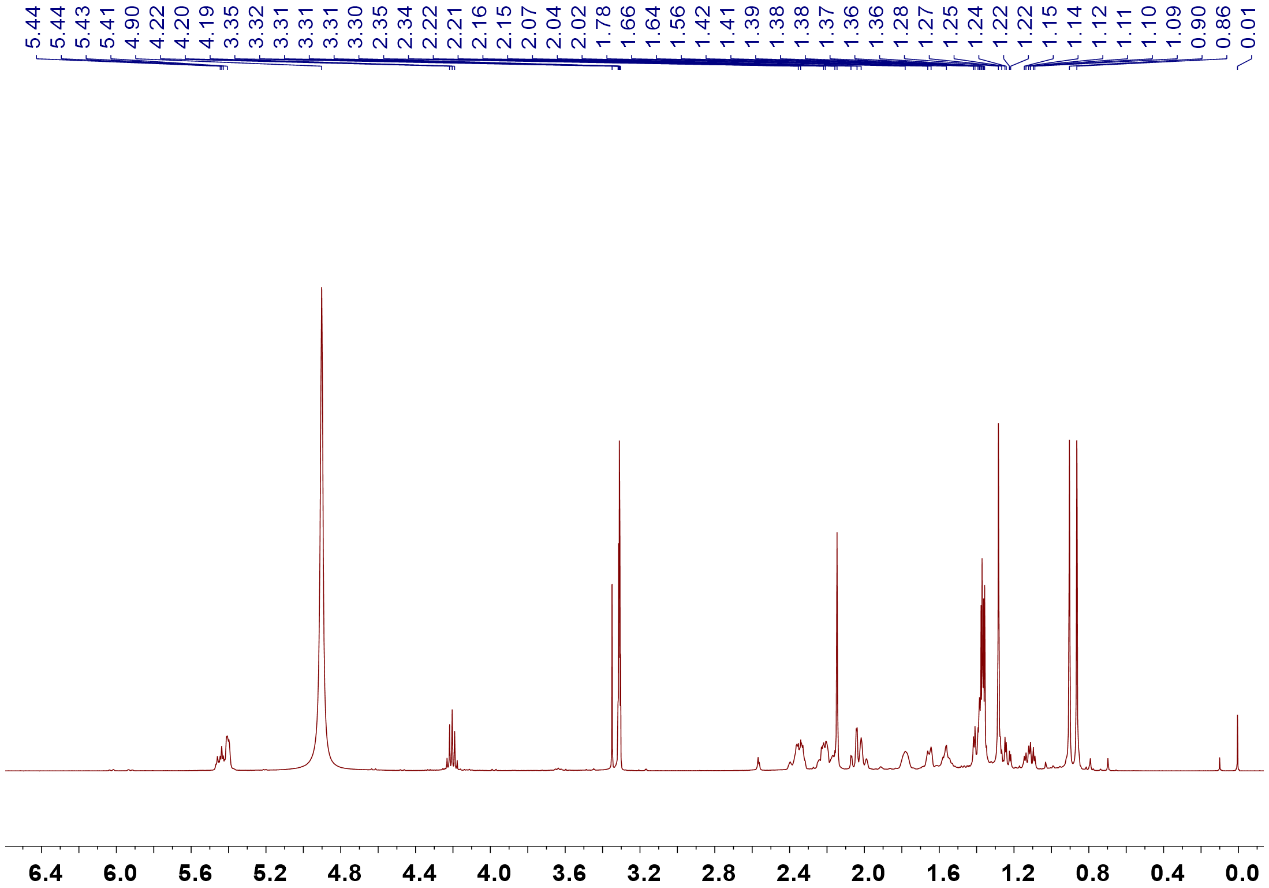


**Supplementary Figure 29.**^1^H NMR spectrum of compound **5** (Methanol-*d*_4_, 500 MHz)


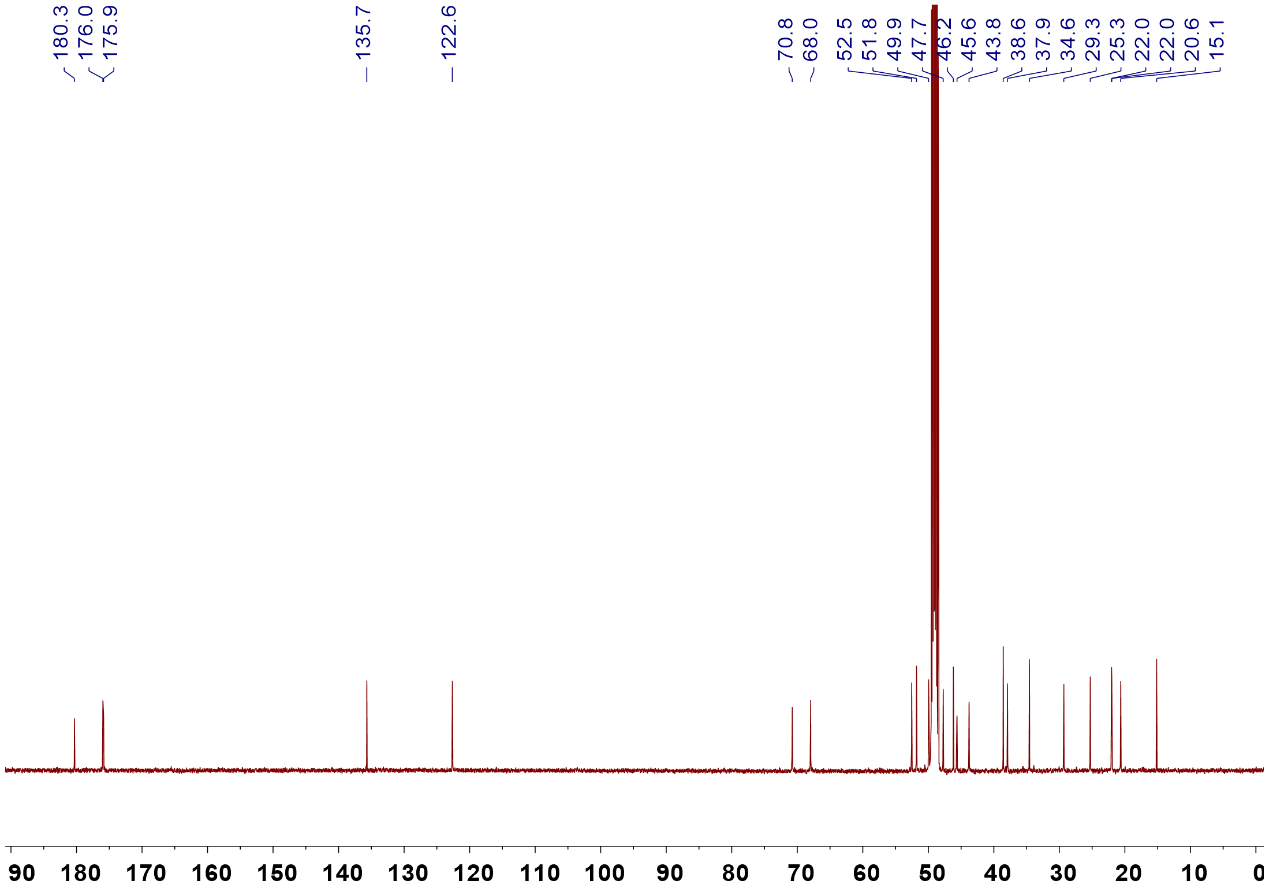


**Supplementary Figure 30.** ^13^C NMR spectrum of compound **5** (Methanol-*d*_4_, 125 MHz)

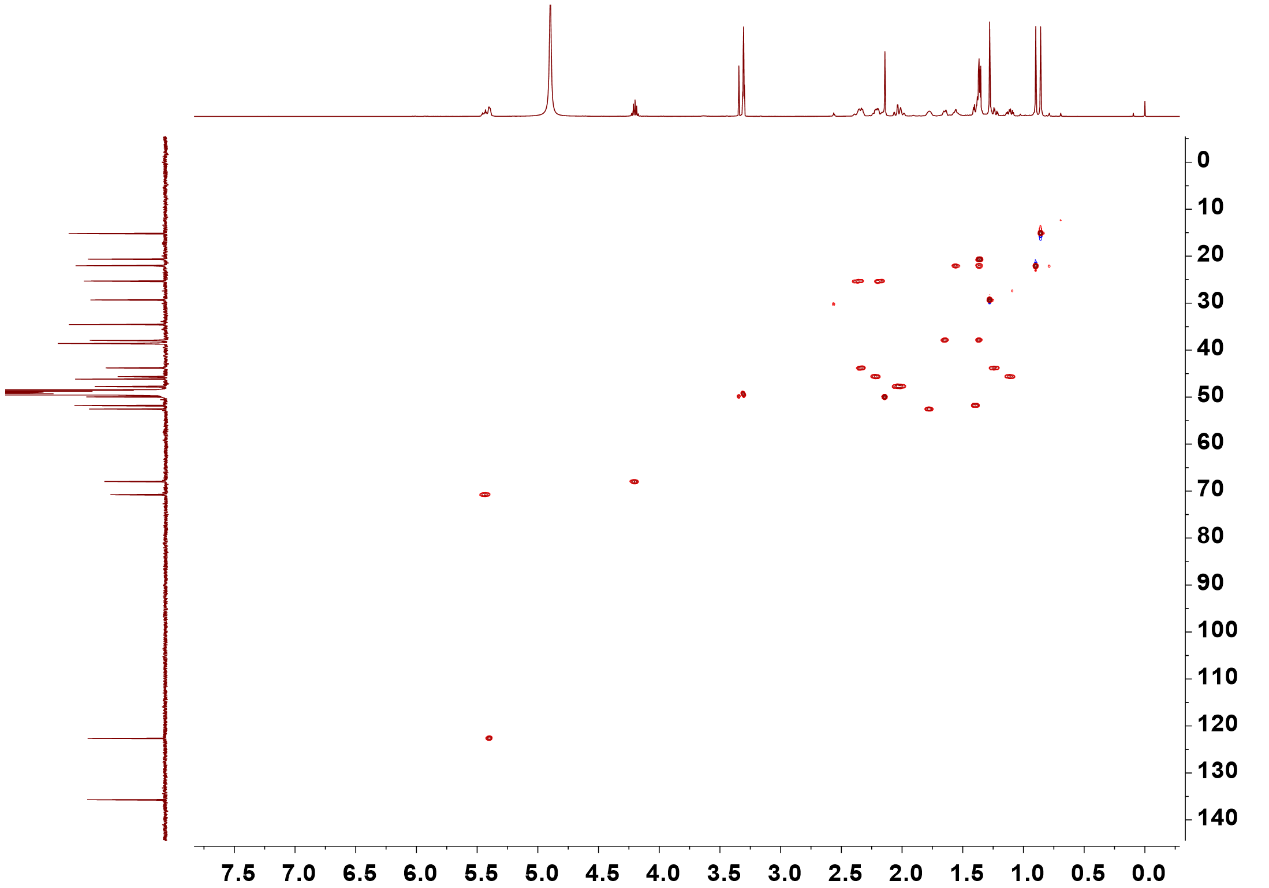


**Supplementary Figure 31.** HSQC spectrum of compound **5** (Methanol-*d*_4_)

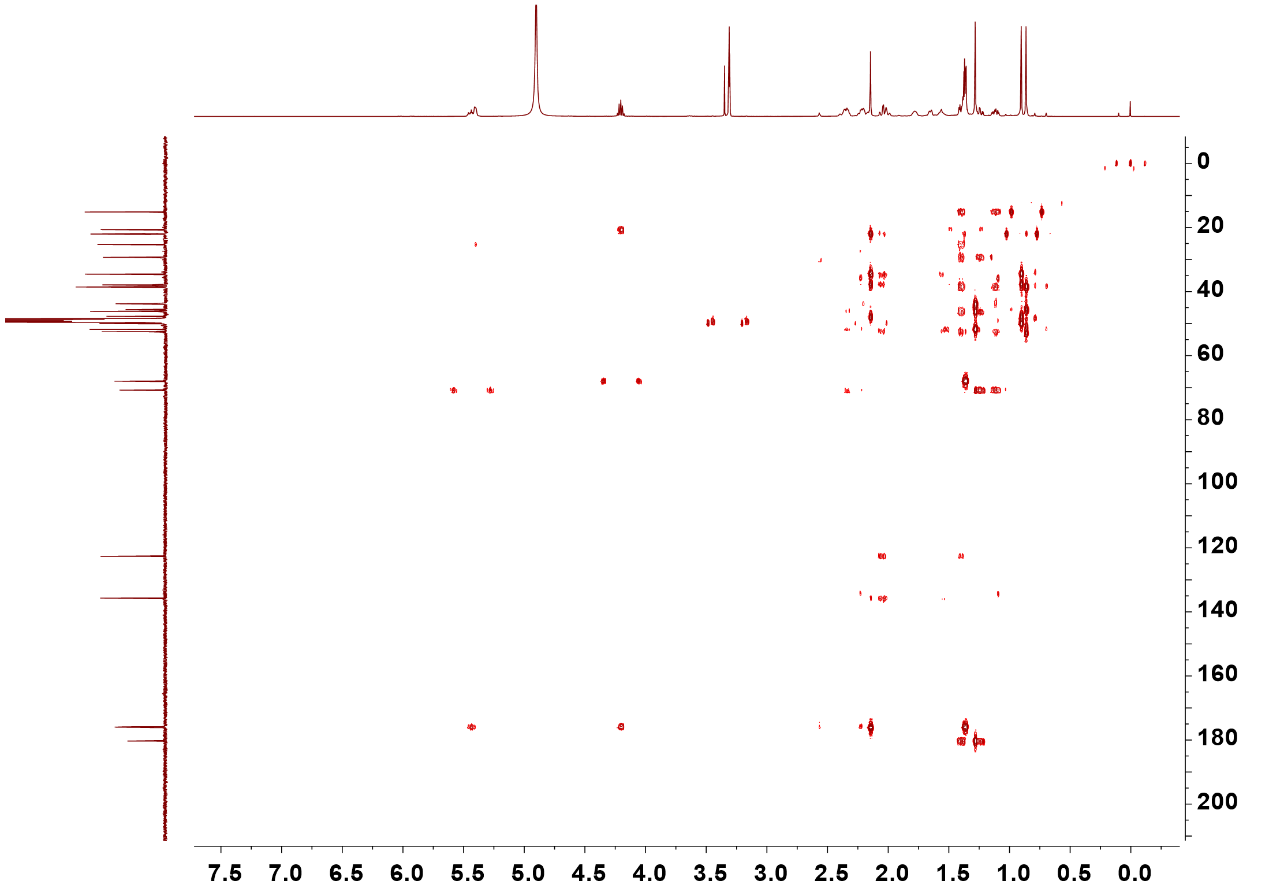


**Supplementary Figure 32.** HMBC spectrum of compound **5** (Methanol-*d*_4_)

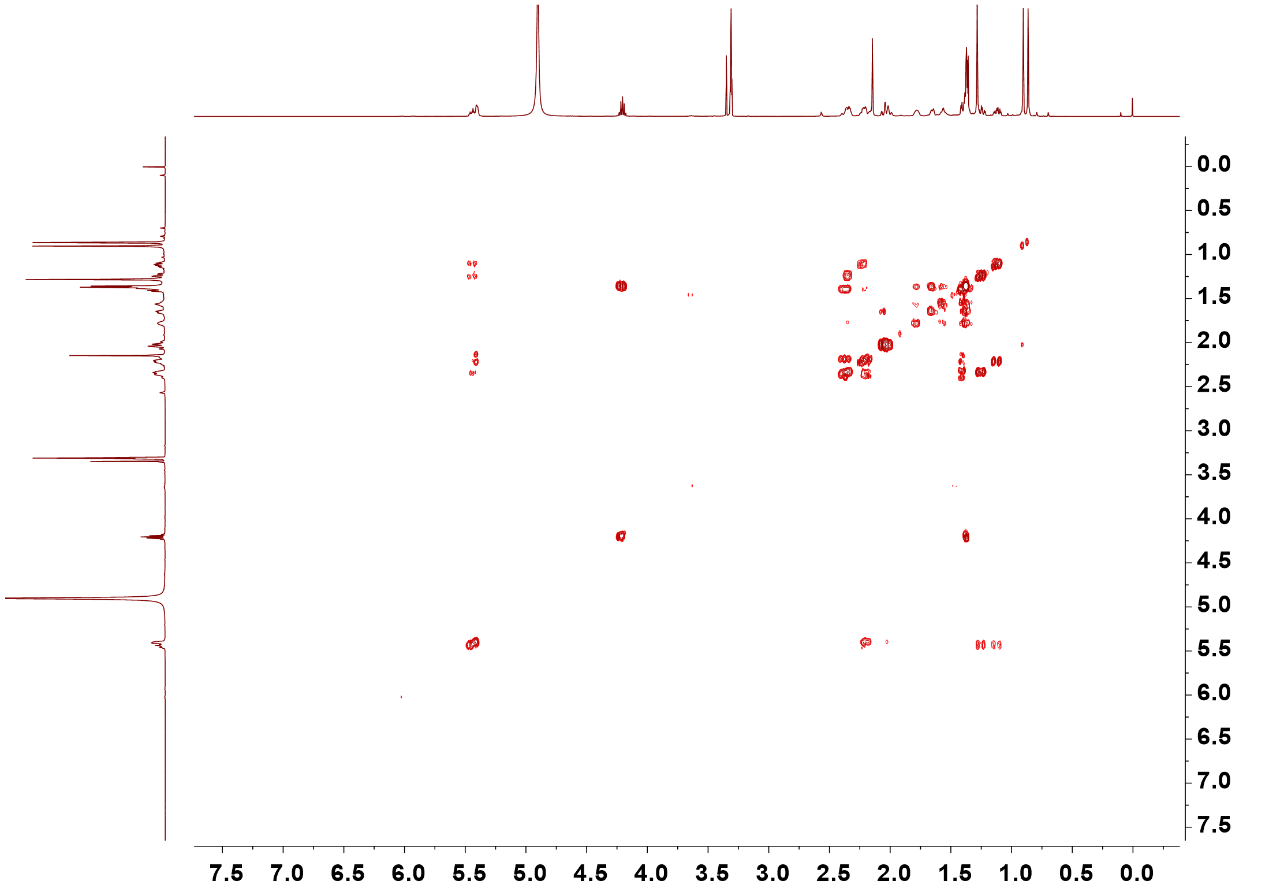


**Supplementary Figure 33.**^1^H-^1^H COSY spectrum of compound **5** (Methanol-*d*_4_)

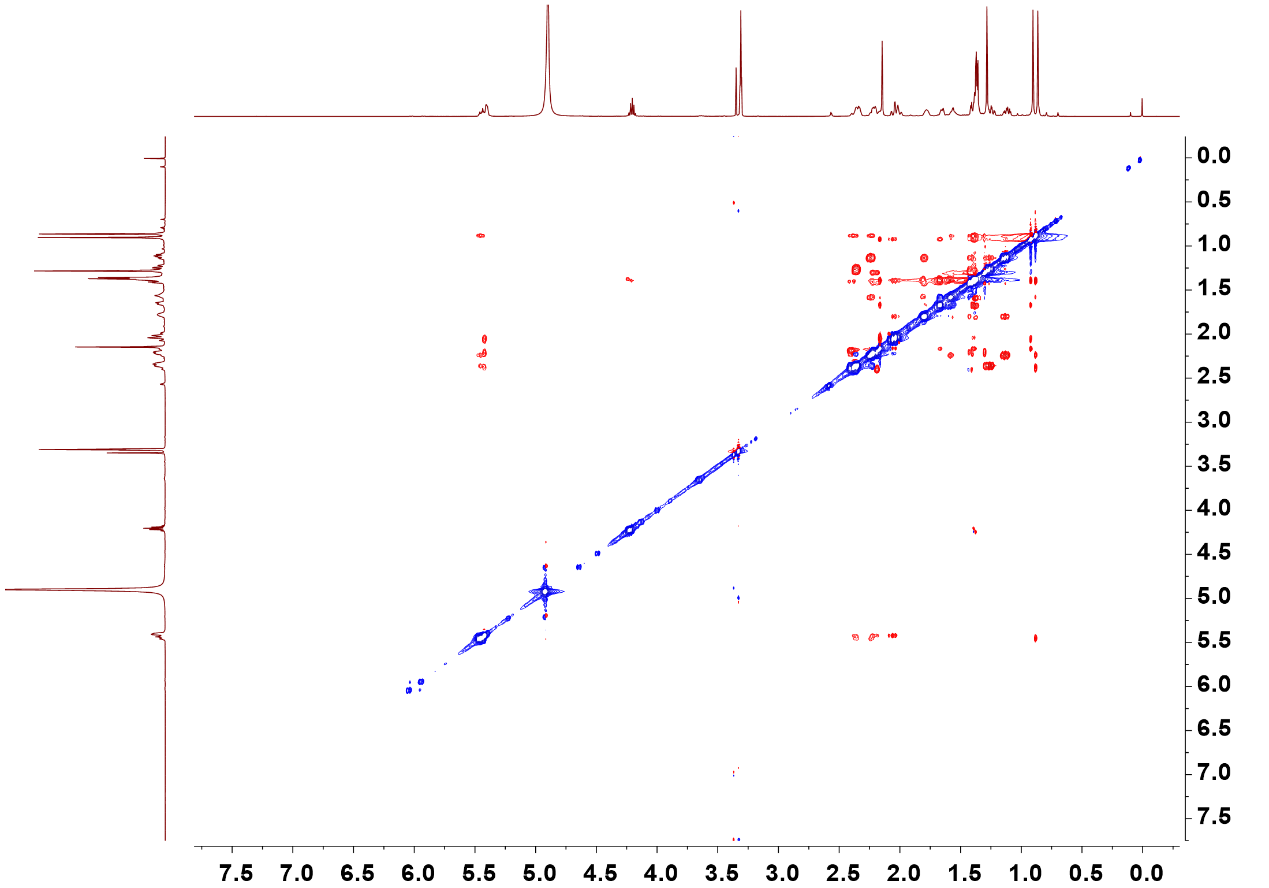


**Supplementary Figure 34.** ROESY spectrum of compound **5** (Methanol-*d*_4_)


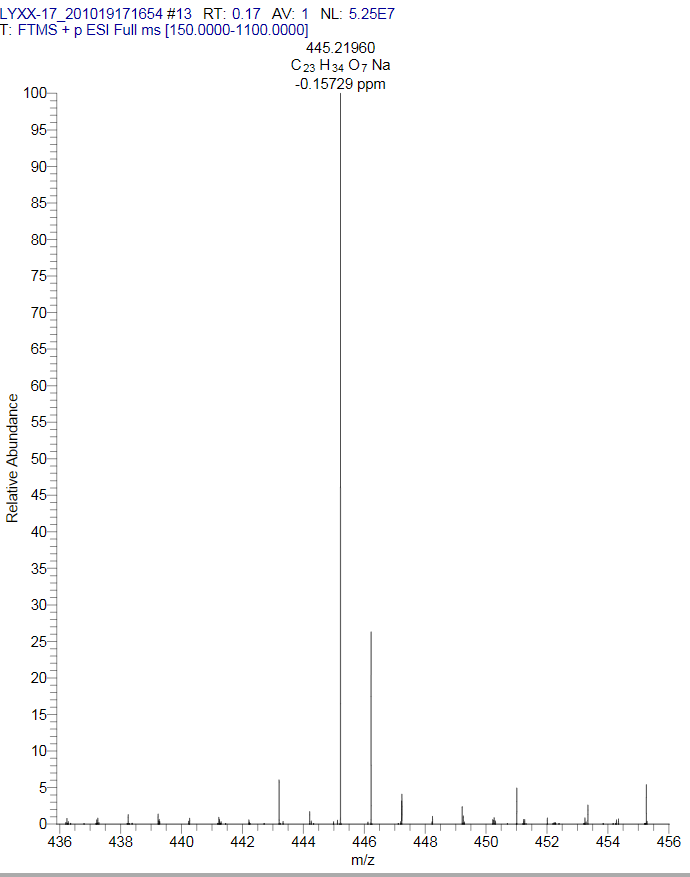


**Supplementary Figure 35.** HRESIMS spectrum of compound **5**

# Standard orientation of the optimized conformers

Standard orientation of (2'*R**)-**4a**

C -9.50809 0.02433 0.61699

C -6.75451 -2.09915 -2.11738

C -9.17789 -1.22753 -1.61085

C -9.93511 -0.1166 -0.84149

O -10.21 1.11939 1.20557

C -9.55155 -1.05972 -3.10556

C -5.63748 -0.28811 0.00416

C -4.77647 -0.9954 -1.03166

C -3.30757 -0.63623 -1.04489

C -5.28847 -1.83094 -1.95136

C -4.94426 -0.28558 1.38618

C -2.6306 -0.68099 0.34706

C -3.48011 0.15857 1.32913

C -2.52046 -2.14429 0.82586

C -1.20835 -0.06884 0.20129

C -0.35957 -0.03421 1.47233

O 0.89928 0.57371 1.13979

C 1.76232 0.68171 2.18582

O 1.54847 0.27947 3.32484

C 3.08972 1.31266 1.75737

C 2.88574 2.64579 1.05811

O 3.94139 1.53775 2.88325

H -7.40888 -0.08556 -1.93221

H -5.67133 0.76022 -0.33068

C -9.67072 -2.63346 -1.25215

O -9.28876 -3.68014 -1.75186

O -10.64295 -2.67336 -0.32603

H -6.50305 -2.8614 0.57966

H -7.06554 -1.91745 1.96045

H -8.22107 -2.57737 0.83809

H -7.78838 1.25999 0.2879

H -7.75261 0.38897 1.79465

H -9.78435 -0.86192 1.19425

H -6.95042 -3.10825 -1.74504

H -6.97045 -2.10161 -3.1911

H -11.02178 -0.26994 -0.88619

H -9.77373 0.8492 -1.34237

H -9.99074 1.92561 0.70845

H -10.6391 -1.10059 -3.24341

H -9.20179 -0.09917 -3.49994

H -9.12746 -1.85297 -3.73024

H -2.75596 -1.28252 -1.74064

H -3.23046 0.38185 -1.45188

H -4.63389 -2.31787 -2.67154

H -4.97476 -1.27917 1.84432

H -5.47879 0.38621 2.0674

H -3.06573 0.10029 2.34244

H -3.44495 1.21651 1.03708

H -2.16757 -2.20146 1.86087

H -3.47937 -2.67054 0.78504

H -1.81883 -2.70755 0.20006

H -0.64962 -0.618 -0.56955

H -1.28591 0.9564 -0.18679

H -0.84677 0.5645 2.2486

H -0.17505 -1.04773 1.84296

H 3.60174 0.60352 1.09873

H 2.35337 3.34855 1.70908

H 3.84974 3.10489 0.81422

H 2.30959 2.53582 0.13428

H 3.68168 0.88449 3.56308

H -10.85509 -3.6269 -0.23923

Standard orientation of (2'*R**)-**4b**

C -7.22073 -2.11938 0.89295

C -7.15776 -0.81177 0.06407

C -8.07442 0.24601 0.72846

C -7.62229 -1.04627 -1.41899

C -9.56145 -0.0632 0.59473

C -6.70139 -2.07675 -2.11282

C -9.15949 -1.2914 -1.63573

C -9.9676 -0.21511 -0.86867

O -10.30869 1.00422 1.17837

C -9.5194 -1.12428 -3.13415

C -5.67498 -0.23676 0.0292

C -4.77695 -0.90826 -0.99907

C -3.32132 -0.49818 -0.99171

C -5.24783 -1.75741 -1.92817

C -5.00017 -0.21974 1.42022

C -2.66121 -0.52868 0.40883

C -3.5514 0.27403 1.38546

C -2.50655 -1.991 0.87871

C -1.2588 0.13237 0.28617

C -0.43477 0.20055 1.5722

O 0.8184 0.82871 1.25478

C 1.67205 0.93988 2.31921

O 1.41317 0.59198 3.46304

C 2.97277 1.6181 1.86431

C 4.14952 1.14333 2.70078

O 3.28581 1.3706 0.49478

H -7.4272 -0.08722 -1.92972

H -5.74052 0.81185 -0.29983

C -9.60508 -2.71727 -1.29452

O -9.19082 -3.74389 -1.80982

O -10.56915 -2.80173 -0.36287

H -7.0738 -1.92175 1.96064

H -8.18646 -2.61962 0.81808

H -6.45566 -2.83925 0.58576

H -7.88391 1.23425 0.28688

H -7.83352 0.36197 1.7927

H -9.8117 -0.96027 1.16694

H -6.86642 -3.09285 -1.74441

H -6.90413 -2.08476 -3.18902

H -11.0468 -0.41196 -0.92471

H -9.84039 0.75877 -1.36383

H -10.11557 1.81855 0.68346

H -10.60273 -1.20418 -3.28716

H -9.20049 -0.14789 -3.51557

H -9.05766 -1.89525 -3.75998

H -2.73897 -1.12047 -1.68428

H -3.27431 0.52447 -1.39174

H -4.56759 -2.21843 -2.64165

H -5.00265 -1.21685 1.87181

H -5.56618 0.42887 2.09852

H -3.14838 0.22171 2.40373

H -3.54855 1.33479 1.10171

H -2.16304 -2.044 1.91706

H -3.44642 -2.54941 0.82372

H -1.77953 -2.52547 0.25659

H -0.66814 -0.39859 -0.47343

H -1.36506 1.15351 -0.10578

H -0.94842 0.80068 2.33006

H -0.23779 -0.80367 1.96092

H 2.83913 2.69844 1.9856

H 4.29399 0.06233 2.59178

H 5.07667 1.62404 2.37097

H 4.00132 1.36114 3.7632

H 2.45096 1.47789 0.00115

H -10.74754 -3.76303 -0.28638

Standard orientation of (2'*R**)-**4c**

C -6.71638 -1.96021 1.01169

C -6.62878 -0.74854 0.04958

C -7.23966 0.49054 0.75089

C -7.39837 -1.02141 -1.29303

C -8.75218 0.41286 0.91865

C -6.78848 -2.24119 -2.02206

C -8.9669 -1.02544 -1.20607

C -9.44295 0.22635 -0.42894

O -9.19422 1.63957 1.49799

C -9.57967 -0.9222 -2.62548

C -5.11521 -0.42345 -0.31314

C -4.53712 -1.3169 -1.40041

C -3.06499 -1.14482 -1.69928

C -5.29524 -2.15196 -2.1315

C -4.18929 -0.39827 0.92322

C -2.15 -1.14866 -0.44779

C -2.7202 -0.14276 0.5766

C -2.10418 -2.57081 0.15326

C -0.71876 -0.7351 -0.91803

C 0.35912 -0.61118 0.16193

O 0.16139 0.60176 0.89925

C 1.01653 0.77406 1.94229

O 1.92685 0.00976 2.24587

C 0.75767 2.09605 2.67037

C -0.69594 2.24559 3.08571

O 1.55988 2.19896 3.84932

H -7.16624 -0.15156 -1.93191

H -5.09365 0.59028 -0.74209

C -9.54529 -2.32298 -0.63315

O -9.3861 -3.44619 -1.08428

O -10.33208 -2.16474 0.44625

H -7.74005 -2.30352 1.16399

H -6.13549 -2.81521 0.65195

H -6.33936 -1.70553 2.00848

H -7.00002 1.39958 0.18126

H -6.78367 0.64824 1.73655

H -9.01278 -0.38346 1.62132

H -7.0283 -3.18384 -1.52281

H -7.19175 -2.30077 -3.03857

H -10.53112 0.21137 -0.28002

H -9.26227 1.12655 -1.03456

H -10.14927 1.55323 1.6634

H -10.67175 -0.83277 -2.57466

H -9.19886 -0.04639 -3.16252

H -9.36652 -1.80727 -3.23407

H -2.72063 -1.91205 -2.4057

H -2.95756 -0.18342 -2.22115

H -4.83859 -2.77339 -2.89915

H -4.24379 -1.34178 1.47545

H -4.51709 0.38487 1.61629

H -2.14716 -0.18326 1.50977

H -2.62378 0.8786 0.18591

H -1.57248 -2.58568 1.11012

H -3.10362 -2.9782 0.33626

H -1.59505 -3.26581 -0.52413

H -0.37216 -1.47233 -1.65446

H -0.78602 0.22696 -1.44354

H 0.35477 -1.47456 0.83434

H 1.33858 -0.55423 -0.3282

H 1.05542 2.91089 2.00245

H -1.00936 1.40464 3.71478

H -0.83776 3.15691 3.67645

H -1.36198 2.28743 2.21866

H 2.35428 1.65056 3.69321

H -10.62764 -3.07751 0.6533

Standard orientation of (2'*R**)-**4d**

C -6.70265 -2.15192 0.97647

C -6.5326 -0.825 0.19412

C -7.12172 0.33092 1.03964

C -7.25319 -0.87366 -1.20215

C -8.64303 0.30396 1.13702

C -6.67368 -2.02235 -2.05866

C -8.81958 -0.79411 -1.18994

C -9.28297 0.34348 -0.24655

O -9.07621 1.4616 1.85112

C -9.35044 -0.42646 -2.60016

C -4.9927 -0.52586 -0.06922

C -4.41026 -1.3017 -1.24132

C -2.92174 -1.16058 -1.46502

C -5.17384 -1.99983 -2.09983

C -4.11704 -0.7049 1.19019

C -2.05467 -1.36664 -0.19546

C -2.62505 -0.47657 0.93201

C -2.0853 -2.8539 0.21585

C -0.59444 -0.95202 -0.56514

C 0.48602 -1.1212 0.50892

O 0.29454 -0.23844 1.62244

C 0.73688 1.04493 1.45087

O 1.3121 1.46374 0.45598

C 0.44116 1.86413 2.7168

C 0.10454 3.30145 2.35583

O -0.64329 1.34942 3.48754

H -6.94366 0.05728 -1.70857

H -4.91111 0.53246 -0.36153

C -9.51776 -2.09313 -0.78005

O -10.10745 -2.3096 0.26623

O -9.45617 -3.06705 -1.70778

H -6.13464 -2.97261 0.52738

H -6.36223 -2.04968 2.01304

H -7.74106 -2.47407 1.03944

H -6.70295 0.32698 2.05391

H -6.8191 1.29819 0.61427

H -8.983 -0.56824 1.70241

H -6.98523 -3.00478 -1.69158

H -7.03325 -1.93145 -3.0891

H -10.37602 0.33507 -0.13311

H -9.05643 1.3185 -0.70225

H -8.74378 1.38195 2.76188

H -8.93106 0.52445 -2.94784

H -9.10942 -1.1852 -3.3512

H -10.4427 -0.3259 -2.59265

H -2.58316 -1.84633 -2.25329

H -2.75382 -0.14637 -1.85433

H -4.71372 -2.53858 -2.9257

H -4.23309 -1.70867 1.61114

H -4.43944 -0.00406 1.96865

H -2.09798 -0.66654 1.87266

H -2.46791 0.58157 0.68565

H -1.57442 -3.47578 -0.52801

H -1.59312 -3.01218 1.18108

H -3.10553 -3.23952 0.30925

H -0.27932 -1.53944 -1.43813

H -0.60541 0.09689 -0.88813

H 0.49321 -2.14258 0.90042

H 1.47977 -0.96036 0.07268

H 1.33797 1.8326 3.34453

H -0.79071 3.34395 1.72477

H -0.11231 3.88545 3.25664

H 0.92522 3.78362 1.81555

H -0.52117 0.38183 3.51458

H -9.95672 -3.80783 -1.30509

Standard orientation of (2'*R**)-**4e**

C -6.83785 -1.02948 1.27317

C -6.87191 -0.39335 -0.13963

C -7.75093 0.88108 -0.0934

C -7.44947 -1.38872 -1.21046

C -9.2366 0.59079 0.08096

C -6.57661 -2.66214 -1.2789

C -8.99523 -1.65391 -1.16081

C -9.75304 -0.30777 -1.03908

O -9.93692 1.83304 0.03103

C -9.47421 -2.30461 -2.48361

C -5.40762 0.02137 -0.60174

C -4.58415 -1.13853 -1.14398

C -3.13856 -0.84022 -1.47317

C -5.11592 -2.34495 -1.40621

C -4.62427 0.78965 0.48713

C -2.36576 -0.10306 -0.34922

C -3.19134 1.1354 0.07318

C -2.16175 -1.05754 0.84546

C -0.99033 0.33951 -0.94273

C -0.03703 1.09053 -0.00928

O 0.53474 0.17799 0.93529

C 1.31865 0.77154 1.88699

O 1.57693 1.96623 1.93618

C 1.86734 -0.30015 2.84087

C 2.05518 0.26791 4.23787

O 1.02366 -1.44517 2.94952

H -7.2987 -0.8772 -2.17717

H -5.51763 0.71014 -1.45356

C -9.45504 -2.57663 -0.02982

O -10.13584 -2.26302 0.93385

O -9.06666 -3.85708 -0.16975

H -6.07962 -1.81461 1.3544

H -6.61545 -0.28083 2.04174

H -7.7918 -1.47046 1.56044

H -7.42642 1.55265 0.71152

H -7.61667 1.4626 -1.01644

H -9.42435 0.15692 1.06714

H -6.70438 -3.29562 -0.39648

H -6.86228 -3.25851 -2.15184

H -10.82917 -0.47677 -0.8946

H -9.68183 0.24604 -1.9865

H -10.86529 1.64736 0.25726

H -9.22216 -1.68033 -3.34828

H -9.03161 -3.29187 -2.64948

H -10.56221 -2.4443 -2.47943

H -2.60354 -1.76143 -1.73993

H -3.13974 -0.21794 -2.37923

H -4.4881 -3.14576 -1.79149

H -4.57419 0.21461 1.41711

H -5.14475 1.72343 0.72843

H -2.71015 1.64995 0.91294

H -3.22925 1.85554 -0.75477

H -1.45735 -1.8575 0.59121

H -1.77443 -0.52648 1.72084

H -3.09314 -1.54121 1.15706

H -0.47133 -0.55145 -1.32132

H -1.17944 0.98589 -1.81001

H 0.7786 1.51117 -0.60975

H -0.54771 1.90989 0.5061

H 2.8301 -0.63364 2.43915

H 1.0981 0.60264 4.65385

H 2.44726 -0.49747 4.91618

H 2.74258 1.11956 4.237

H 0.73301 -1.65614 2.04227

H -9.44556 -4.30935 0.61368

Standard orientation of (2'*R**)-**4f**

C -7.50944 0.16021 1.10479

C -6.86151 -0.0961 -0.27952

C -7.71062 0.60741 -1.36699

C -6.76569 -1.63186 -0.60045

C -9.06223 -0.05335 -1.60779

C -5.91481 -2.34654 0.47337

C -8.10692 -2.3585 -0.96629

C -8.8922 -1.51714 -2.00377

O -9.70599 0.63894 -2.67689

C -7.81425 -3.72152 -1.64396

C -5.38818 0.50032 -0.3266

C -4.34482 -0.38937 0.33384

C -2.94329 0.17131 0.44212

C -4.60953 -1.64964 0.72055

C -5.30151 1.94426 0.21597

C -2.87018 1.6068 1.00559

C -3.87452 2.49454 0.2237

C -3.22103 1.6027 2.50945

C -1.45289 2.23267 0.8005

C -0.26358 1.50251 1.43003

O 0.01053 0.30363 0.69508

C 1.00014 -0.47247 1.23435

O 1.65782 -0.18011 2.22348

C 1.19103 -1.73109 0.3749

C 1.66828 -2.89618 1.22617

O 0.00091 -2.14519 -0.2931

H -6.17234 -1.68639 -1.52989

H -5.08909 0.54471 -1.38536

C -9.026 -2.65003 0.22209

O -10.11305 -2.14015 0.44357

O -8.55701 -3.58736 1.06554

H -6.85435 -0.13811 1.92933

H -7.74498 1.22121 1.24395

H -8.45269 -0.36989 1.23311

H -7.87488 1.66265 -1.11458

H -7.15963 0.62789 -2.31784

H -9.6997 0.05606 -0.72586

H -6.43968 -2.42219 1.43002

H -5.68769 -3.36639 0.14547

H -9.88267 -1.95426 -2.19184

H -8.38075 -1.5544 -2.97659

H -10.60766 0.27956 -2.74845

H -7.19307 -3.59615 -2.53797

H -7.29603 -4.41839 -0.97768

H -8.74521 -4.21336 -1.95145

H -2.3274 -0.49761 1.05518

H -2.51115 0.15818 -0.5678

H -3.83181 -2.25244 1.18614

H -5.69742 2.01072 1.2337

H -5.9198 2.60851 -0.39858

H -3.88743 3.50772 0.64541

H -3.54134 2.59801 -0.81788

H -2.54163 0.9611 3.07945

H -3.15851 2.61311 2.92853

H -4.23118 1.22864 2.70195

H -1.2628 2.31902 -0.27788

H -1.46669 3.25529 1.20027

H 0.61792 2.15155 1.36303

H -0.44727 1.27356 2.48422

H 1.93849 -1.49568 -0.39039

H 0.9286 -3.14404 1.99596

H 1.80203 -3.79333 0.61234

H 2.61577 -2.66877 1.72466

H -0.38901 -1.34034 -0.68252

H -9.25525 -3.66672 1.74952

Standard orientation of (2'*R**)-**4g**

C -6.7986 0.45651 -0.65534

C -6.67494 -1.08815 -0.63336

C -7.02831 -1.63819 -2.03739

C -7.62952 -1.73209 0.43683

C -8.50676 -1.51599 -2.38541

C -7.28444 -1.2001 1.8462

C -9.16118 -1.74315 0.09633

C -9.37158 -2.23872 -1.35675

O -8.71008 -2.12127 -3.66148

C -9.91002 -2.7467 1.01024

C -5.18707 -1.525 -0.28023

C -4.87006 -1.47234 1.20776

C -3.43504 -1.74378 1.59997

C -5.81669 -1.28793 2.14432

C -4.12052 -0.75946 -1.09604

C -2.38526 -0.94075 0.78906

C -2.68481 -1.13282 -0.71651

C -2.4675 0.55043 1.1766

C -0.98064 -1.5263 1.14146

C 0.23399 -0.8925 0.45845

O 0.48409 0.40085 1.01937

C 1.54775 1.05703 0.4668

O 2.24488 0.62893 -0.44312

C 1.70442 2.43936 1.11088

C 1.12096 3.49866 0.19144

O 1.08702 2.55428 2.39087

H -7.35311 -2.80088 0.45038

H -5.08638 -2.58723 -0.55153

C -9.86636 -0.39469 0.25876

O -10.32944 0.29674 -0.63452

O -9.98482 0.01585 1.53452

H -6.37313 0.91802 0.24127

H -6.27945 0.88524 -1.5198

H -7.83096 0.79568 -0.73443

H -6.44056 -1.13473 -2.81537

H -6.74097 -2.69677 -2.10828

H -8.7873 -0.4634 -2.48225

H -7.58979 -0.15807 1.97779

H -7.81112 -1.79165 2.60257

H -10.42666 -2.15005 -1.65089

H -9.15382 -3.31493 -1.41798

H -9.63059 -1.94028 -3.92082

H -9.49393 -3.75624 0.91762

H -9.86315 -2.46669 2.06731

H -10.97321 -2.799 0.74534

H -3.28489 -1.56074 2.67241

H -3.26232 -2.81879 1.44958

H -5.54392 -1.25737 3.1973

H -4.22995 0.32245 -0.97149

H -4.25494 -0.96321 -2.16435

H -2.00353 -0.52588 -1.32406

H -2.50798 -2.17903 -0.99878

H -2.1114 0.70899 2.20076

H -1.86635 1.17294 0.50611

H -3.49087 0.93681 1.13217

H -0.83815 -1.45746 2.22844

H -0.9778 -2.59549 0.891

H 1.10913 -1.52544 0.64973

H 0.08905 -0.81662 -0.62364

H 2.77505 2.61128 1.26196

H 0.04354 3.34808 0.05784

H 1.25202 4.49701 0.62201

H 1.59254 3.47573 -0.79607

H 0.22749 2.09724 2.32363

H -10.46219 0.86997 1.46831

Standard orientation of (2'*R**)-**4h**

C -6.95016 -0.20908 -1.81909

C -6.53516 -1.32465 -0.82643

C -7.12812 -2.67058 -1.3112

C -7.0416 -1.02396 0.63065

C -8.64207 -2.75747 -1.16234

C -6.45393 0.31495 1.13026

C -8.57784 -1.20473 0.89308

C -9.06034 -2.54808 0.29003

O -9.05048 -4.06139 -1.57367

C -8.85825 -1.28714 2.41559

C -4.95167 -1.46136 -0.76922

C -4.27715 -0.41616 0.10895

C -2.76367 -0.39951 0.10453

C -4.96979 0.39711 0.92581

C -4.29792 -1.5098 -2.16838

C -2.12636 -0.4228 -1.30164

C -2.77095 -1.57746 -2.11342

C -2.36448 0.92719 -2.0121

C -0.59227 -0.71471 -1.23269

C 0.27538 0.26572 -0.43877

O 0.02185 0.10608 0.96167

C 0.66782 1.00764 1.75969

O 1.40341 1.90102 1.36228

C 0.28253 0.78039 3.22601

C -0.79349 1.77449 3.62903

O -0.18498 -0.53739 3.50357

H -6.57202 -1.80327 1.25591

H -4.72549 -2.42359 -0.28421

C -9.4596 -0.07961 0.34669

O -10.25651 -0.16139 -0.57465

O -9.32475 1.09138 0.99549

H -6.76697 -0.51088 -2.85642

H -8.01186 0.02967 -1.76504

H -6.39933 0.72132 -1.64921

H -6.86599 -2.86019 -2.35978

H -6.6756 -3.50229 -0.75295

H -9.13064 -2.0433 -1.83122

H -6.90789 1.17471 0.62904

H -6.64628 0.42473 2.20267

H -10.15287 -2.6395 0.36438

H -8.66857 -3.38533 0.88567

H -10.02369 -4.06907 -1.577

H -8.29809 -2.10687 2.87949

H -8.58996 -0.36579 2.94201

H -9.92403 -1.46106 2.60825

H -2.40744 0.47822 0.65673

H -2.42822 -1.28131 0.66741

H -4.4489 1.1312 1.53702

H -4.57853 -0.63927 -2.76857

H -4.65861 -2.38868 -2.71486

H -2.37918 -1.58011 -3.13864

H -2.4863 -2.54204 -1.67177

H -1.94651 1.76175 -1.44013

H -1.89995 0.93451 -3.00442

H -3.42786 1.14792 -2.14522

H -0.44767 -1.71585 -0.80435

H -0.20097 -0.75181 -2.25806

H 1.3299 0.02765 -0.62283

H 0.09386 1.29938 -0.7487

H 1.18458 0.92724 3.82878

H -1.70904 1.61548 3.04788

H -1.06002 1.64727 4.68351

H -0.46544 2.80652 3.47

H -0.7887 -0.77649 2.77555

H -9.95664 1.6902 0.54385

Standard orientation of (2'*R**)-**4i**

C -6.90332 0.18845 -1.32474

C -6.72831 -1.27331 -0.84069

C -7.32723 -2.22556 -1.90461

C -7.43616 -1.51931 0.54116

C -8.84927 -2.18131 -1.97904

C -6.84552 -0.57795 1.61488

C -9.00307 -1.59268 0.52661

C -9.47589 -2.51048 -0.62812

O -9.28806 -3.16472 -2.91617

C -9.52365 -2.24231 1.83513

C -5.18614 -1.62141 -0.66189

C -4.59016 -1.11194 0.64302

C -3.09847 -1.29218 0.82245

C -5.34563 -0.61085 1.63606

C -4.32436 -1.18911 -1.87035

C -2.25429 -0.80857 -0.37766

C -2.82996 -1.44835 -1.66942

C -2.30812 0.73017 -0.47461

C -0.77693 -1.30097 -0.24458

C 0.00467 -0.86245 0.99677

O 0.32715 0.52883 0.90397

C 0.96695 1.03046 2.00271

O 1.30586 0.37707 2.98015

C 1.25199 2.52403 1.80909

C 2.69258 2.7158 1.36742

O 0.39388 3.15726 0.86299

H -7.12519 -2.53641 0.83729

H -5.10572 -2.71757 -0.59701

C -9.7016 -0.23662 0.39909

O -10.30626 0.18708 -0.57288

O -9.6212 0.52965 1.50298

H -6.57582 0.30596 -2.36376

H -7.94134 0.51742 -1.30672

H -6.32763 0.89621 -0.72017

H -6.91869 -2.00729 -2.89942

H -7.02098 -3.26023 -1.69544

H -9.1946 -1.21076 -2.34611

H -7.15665 0.46026 1.46637

H -7.19615 -0.88338 2.60641

H -10.56999 -2.48082 -0.72696

H -9.24497 -3.55848 -0.38753

H -8.98735 -2.88015 -3.79639

H -9.27638 -1.65353 2.72403

H -10.61603 -2.33989 1.81561

H -9.1023 -3.24395 1.97705

H -2.768 -0.78584 1.73774

H -2.91704 -2.3639 0.98284

H -4.87795 -0.2567 2.55268

H -4.45656 -0.12651 -2.09337

H -4.64706 -1.73173 -2.76635

H -2.28431 -1.07764 -2.5466

H -2.67238 -2.5352 -1.64476

H -2.00458 1.20198 0.46492

H -1.64932 1.09671 -1.26945

H -3.31493 1.10246 -0.68596

H -0.77543 -2.39911 -0.25821

H -0.22152 -0.97502 -1.13441

H -0.55856 -1.06563 1.91307

H 0.94313 -1.42909 1.03336

H 1.07788 3.01799 2.77058

H 2.86649 2.24843 0.39147

H 2.92286 3.7806 1.25604

H 3.39308 2.27624 2.0844

H 0.30594 2.53893 0.11334

H -10.12066 1.33959 1.26571

Standard orientation of (2'*S**)-**4a**

C -7.67063 0.48216 0.22807

C -6.71928 0.09172 -0.93096

C -7.19915 0.79127 -2.22722

C -6.6916 -1.46366 -1.15482

C -8.50357 0.22895 -2.78066

C -6.19242 -2.18139 0.12097

C -7.97625 -2.09565 -1.80127

C -8.38904 -1.26904 -3.0434

O -8.78955 0.87165 -4.02267

C -7.66711 -3.52697 -2.30988

C -5.23203 0.56608 -0.62609

C -4.4699 -0.36311 0.31022

C -3.09868 0.10292 0.75012

C -4.93137 -1.57517 0.66187

C -5.14992 2.0361 -0.14793

C -3.08265 1.54602 1.29516

C -3.7318 2.47077 0.23683

C -3.88138 1.61106 2.62204

C -1.63764 2.03503 1.6315

C -0.58051 1.94261 0.52677

O -0.13853 0.58926 0.33606

C 0.87588 0.16954 1.15345

O 1.39047 0.84085 2.03692

C 1.21923 -1.29315 0.83324

C 2.69434 -1.56576 1.07734

O 0.92843 -1.65982 -0.51399

H -4.67609 0.51861 -1.57541

H -5.90265 -1.62477 -1.91

C -9.12926 -2.28462 -0.8105

O -9.1042 -2.96597 0.20268

O -10.26983 -1.65335 -1.13691

H -7.82843 1.56562 0.26739

H -8.66409 0.04578 0.12159

H -7.28128 0.17356 1.2032

H -6.42827 0.7065 -3.0061

H -7.32 1.87023 -2.06765

H -9.33661 0.44926 -2.10747

H -6.93704 -2.16594 0.92148

H -5.98294 -3.23193 -0.10703

H -9.33462 -1.63431 -3.46683

H -7.65256 -1.42065 -3.84638

H -8.93872 1.81454 -3.83655

H -6.81514 -3.53122 -2.99885

H -7.43894 -4.21771 -1.49126

H -8.52899 -3.94698 -2.84295

H -2.46215 0.03283 -0.13987

H -2.66981 -0.57894 1.49556

H -4.35378 -2.20622 1.33434

H -5.80132 2.20992 0.71356

H -5.50772 2.70253 -0.94118

H -3.76435 3.50321 0.60686

H -3.12667 2.48745 -0.67703

H -4.90976 1.25232 2.51968

H -3.40644 0.99378 3.39362

H -3.92718 2.63866 3.00034

H -1.6999 3.08636 1.94295

H -1.27553 1.47251 2.50172

H -0.96598 2.29862 -0.43207

H 0.27485 2.58587 0.76763

H 0.60788 -1.92328 1.48812

H 2.97427 -1.36421 2.1161

H 3.31828 -0.9401 0.42894

H 2.93813 -2.60794 0.84483

H 0.04701 -1.29152 -0.71253

H -10.89623 -1.91667 -0.4295

Standard orientation of (2'*S**)-**4b**

C -7.16683 0.05632 0.55939

C -6.84581 0.03128 -0.95657

C -7.66659 1.14092 -1.65825

C -7.18589 -1.36082 -1.60255

C -9.161 0.84659 -1.7245

C -6.3741 -2.47869 -0.9101

C -8.70547 -1.69001 -1.80974

C -9.42899 -0.47366 -2.4389

O -9.79755 1.88383 -2.47047

C -8.87007 -2.85663 -2.81862

C -5.29937 0.31127 -1.2039

C -4.41364 -0.9076 -0.98196

C -2.91928 -0.68402 -1.06729

C -4.91463 -2.14399 -0.81257

C -4.77156 1.5311 -0.41491

C -2.4088 0.5127 -0.2333

C -3.26829 1.75532 -0.5879

C -2.52219 0.1962 1.27114

C -0.93283 0.85549 -0.61385

C 0.1279 -0.22255 -0.36694

O 0.42959 -0.37387 1.02796

C 1.34708 0.49238 1.53642

O 1.92568 1.36929 0.90283

C 1.64235 0.19988 3.01028

C 0.37953 0.16896 3.8533

O 2.5117 1.18982 3.56627

H -5.17953 0.55423 -2.2711

H -6.78526 -1.29764 -2.62932

C -9.44492 -2.10069 -0.53389

O -10.27219 -1.44356 0.07694

O -9.12176 -3.32696 -0.08145

H -8.1746 -0.29106 0.7826

H -6.47444 -0.56188 1.13935

H -7.10978 1.07377 0.96209

H -7.29689 1.28883 -2.68274

H -7.51905 2.10935 -1.16378

H -9.60499 0.84986 -0.72504

H -6.73993 -2.68513 0.10013

H -6.46202 -3.40758 -1.48347

H -10.5141 -0.643 -2.47821

H -9.12463 -0.36372 -3.49004

H -9.72659 2.70153 -1.94836

H -8.4074 -2.61714 -3.7828

H -8.42009 -3.78826 -2.4617

H -9.93067 -3.06839 -3.00176

H -2.38706 -1.59701 -0.77368

H -2.67652 -0.51608 -2.12567

H -4.24559 -2.98788 -0.65699

H -4.97883 1.4331 0.65458

H -5.29167 2.43757 -0.74529

H -2.96399 2.61025 0.02947

H -3.0832 2.04642 -1.63078

H -3.55739 0.04599 1.59182

H -1.99031 -0.72531 1.52713

H -2.10676 1.01058 1.8745

H -0.90316 1.10087 -1.68397

H -0.64093 1.7678 -0.0778

H -0.19963 -1.19728 -0.7386

H 1.04322 0.01778 -0.92224

H 2.16731 -0.7595 3.06327

H -0.29534 -0.63412 3.54264

H -0.16227 1.11844 3.77655

H 0.62466 0.02637 4.91131

H 3.01911 1.5648 2.81893

H -9.68387 -3.44055 0.71414

Standard orientation of (2'*S**)-**4c**

C -7.19104 0.18983 0.48818

C -6.83324 0.04396 -1.01242

C -7.64996 1.08061 -1.82343

C -7.1498 -1.40048 -1.54343

C -9.14087 0.76926 -1.88945

C -6.32698 -2.45023 -0.76138

C -8.66871 -1.75958 -1.71989

C -9.38795 -0.61651 -2.4766

O -9.77198 1.72495 -2.74157

C -8.81645 -3.02733 -2.60022

C -5.28596 0.32414 -1.25349

C -4.38758 -0.86143 -0.93139

C -2.89547 -0.6233 -1.01367

C -4.87368 -2.08861 -0.67705

C -4.78692 1.60495 -0.54647

C -2.41285 0.63625 -0.26

C -3.28415 1.83853 -0.71204

C -2.54289 0.4251 1.2623

C -0.93686 0.97294 -0.64694

C 0.13495 -0.07334 -0.32298

O 0.38328 -0.1709 1.08632

C 1.24955 0.75088 1.60463

O 1.84109 1.60721 0.96115

C 1.416 0.52505 3.1119

C 2.70534 -0.23328 3.37659

O 0.33492 -0.18342 3.71306

H -5.15066 0.49083 -2.33341

H -6.74726 -1.41666 -2.57124

C -9.36575 -2.12535 -0.40574

O -9.07598 -3.05811 0.32756

O -10.408 -1.3435 -0.07665

H -8.20459 -0.14233 0.71334

H -6.51133 -0.37779 1.13138

H -7.14624 1.23658 0.80889

H -7.26348 1.14123 -2.8506

H -7.51809 2.08986 -1.41351

H -9.6012 0.86809 -0.90251

H -6.69494 -2.58625 0.25932

H -6.39768 -3.42083 -1.26381

H -9.05821 -0.60689 -3.52603

H -10.47018 -0.79705 -2.53157

H -9.67215 2.59835 -2.32533

H -9.8731 -3.24192 -2.80233

H -8.31107 -2.90564 -3.56479

H -8.40329 -3.91874 -2.11659

H -2.35447 -1.50525 -0.64933

H -2.64052 -0.52698 -2.07813

H -4.196 -2.90907 -0.44979

H -5.0103 1.58061 0.52399

H -5.31361 2.47783 -0.94901

H -3.0871 2.05961 -1.76982

H -3.00118 2.73843 -0.15101

H -3.58184 0.29981 1.58142

H -2.0177 -0.47804 1.58806

H -2.1309 1.27771 1.81321

H -0.89695 1.15362 -1.72954

H -0.66251 1.92 -0.16457

H -0.16125 -1.06632 -0.67263

H 1.06654 0.1655 -0.85101

H 1.44795 1.51015 3.58856

H 3.57191 0.29471 2.96631

H 2.67024 -1.23154 2.92565

H 2.85512 -0.37488 4.45218

H 0.09643 -0.89926 3.09449

H -10.74769 -1.7394 0.75393

Standard orientation of (2'*S**)-**4d**

C -7.49888 0.83321 -0.00224

C -6.69685 0.13564 -1.1296

C -7.30797 0.53438 -2.49618

C -6.72947 -1.42822 -0.97912

C -8.68109 -0.0758 -2.75427

C -6.1032 -1.8493 0.37037

C -8.09291 -2.13164 -1.31543

C -8.63247 -1.59753 -2.66457

O -9.09742 0.27297 -4.07441

C -7.87319 -3.65463 -1.50407

C -5.17412 0.59518 -1.10702

C -4.32835 -0.12464 -0.06776

C -2.90182 0.3524 0.1012

C -4.77461 -1.19631 0.61031

C -5.00557 2.12613 -0.98704

C -2.75126 1.88267 0.24208

C -3.54158 2.56187 -0.90798

C -3.29786 2.34376 1.61025

C -1.26063 2.32829 0.09395

C -0.25375 1.78399 1.11095

O -0.03868 0.38704 0.88276

C 0.82614 -0.19815 1.76476

O 1.43552 0.38938 2.64829

C 0.96848 -1.69187 1.4517

C 2.22949 -1.92591 0.63773

O -0.14214 -2.24298 0.74851

H -4.73379 0.30654 -2.07401

H -6.03604 -1.79939 -1.75379

C -9.1254 -2.03239 -0.18808

O -8.99999 -2.4707 0.94492

O -10.27834 -1.42856 -0.52313

H -8.50683 0.43227 0.10655

H -7.00553 0.7446 0.97064

H -7.627 1.90194 -0.2069

H -6.63568 0.22981 -3.31065

H -7.38395 1.62525 -2.58765

H -9.42366 0.33668 -2.0657

H -6.74959 -1.60806 1.21869

H -5.94588 -2.93309 0.38012

H -8.00104 -1.96882 -3.48524

H -9.6298 -2.00385 -2.88151

H -9.1983 1.23989 -4.10291

H -8.7998 -4.14634 -1.82483

H -7.10897 -3.85757 -2.26259

H -7.56275 -4.14708 -0.57653

H -2.45096 -0.1443 0.96852

H -2.34042 0.01415 -0.78053

H -4.13489 -1.68412 1.34378

H -5.52462 2.51495 -0.10598

H -5.46109 2.61771 -1.85422

H -3.06277 2.33235 -1.86951

H -3.5026 3.65307 -0.79684

H -4.36013 2.113 1.73546

H -2.77682 1.85157 2.43769

H -3.17882 3.42578 1.73462

H -0.91218 2.04646 -0.90898

H -1.2216 3.42473 0.14332

H 0.69952 2.30731 0.9675

H -0.59146 1.95468 2.13782

H 1.02773 -2.22213 2.40779

H 3.11722 -1.55509 1.15963

H 2.16774 -1.41833 -0.33164

H 2.3618 -2.99302 0.43033

H -0.3699 -1.60604 0.04583

H -10.82292 -1.49316 0.29011

Standard orientation of (2'*S**)-**4e**

C -7.06086 1.09011 -1.12403

C -6.35909 -0.28806 -1.03143

C -6.09762 -0.8122 -2.46553

C -7.23336 -1.33016 -0.24471

C -7.36363 -1.22926 -3.20534

C -7.48999 -0.83346 1.19717

C -8.50755 -1.87139 -0.98638

C -8.1298 -2.29371 -2.427

O -6.9966 -1.7882 -4.46648

C -9.03104 -3.14834 -0.27964

C -4.963 -0.16054 -0.28116

C -5.08077 -0.12365 1.23739

C -3.80355 0.13213 2.00907

C -6.23017 -0.39117 1.88062

C -4.09901 1.01315 -0.80245

C -2.99582 1.34406 1.49058

C -2.7855 1.17295 -0.0309

C -3.78198 2.64728 1.78392

C -1.64027 1.47165 2.25736

C -0.60483 0.36083 2.06289

O 0.02865 0.51909 0.7873

C 0.90382 -0.47926 0.46363

O 1.20306 -1.41741 1.18958

C 1.5007 -0.22215 -0.9248

C 2.88385 0.38904 -0.7817

O 0.70337 0.63162 -1.74316

H -4.39662 -1.08085 -0.49283

H -6.58546 -2.21774 -0.13934

C -9.69284 -0.90184 -0.94926

O -10.26077 -0.49657 0.05327

O -10.14336 -0.50626 -2.15192

H -8.08394 1.01669 -1.49362

H -7.09917 1.59953 -0.15612

H -6.54148 1.7558 -1.82234

H -5.41699 -1.67451 -2.43129

H -5.56948 -0.06091 -3.06616

H -7.99446 -0.36054 -3.41268

H -8.19445 0.00244 1.22629

H -7.92298 -1.64353 1.79354

H -7.51198 -3.20322 -2.39274

H -9.01957 -2.58888 -2.99977

H -6.55502 -1.08946 -4.97897

H -9.86905 -3.58706 -0.83521

H -8.24837 -3.91118 -0.20084

H -9.39943 -2.94197 0.73074

H -4.01417 0.26206 3.07871

H -3.20269 -0.78149 1.92609

H -6.27035 -0.35136 2.96731

H -4.64239 1.96137 -0.75376

H -3.85633 0.85177 -1.85904

H -2.18499 0.27861 -0.23215

H -2.23047 2.02816 -0.43547

H -4.79353 2.63848 1.36739

H -3.88497 2.80566 2.86391

H -3.26226 3.51848 1.36916

H -1.17057 2.42355 1.97436

H -1.86058 1.53487 3.33142

H 0.1651 0.4624 2.83722

H -1.05626 -0.63196 2.14782

H 1.55941 -1.18592 -1.44083

H 3.54646 -0.25385 -0.19377

H 2.82972 1.36526 -0.28654

H 3.3353 0.55578 -1.76537

H 0.39066 1.35255 -1.16455

H -10.91286 0.06381 -1.94053

Standard orientation of (2'*S**)-**4f**

C -6.84526 0.94953 -1.25142

C -6.40628 -0.5213 -1.03805

C -6.20281 -1.18668 -2.42246

C -7.47939 -1.33027 -0.22316

C -7.50171 -1.42125 -3.1844

C -7.67887 -0.70026 1.1748

C -8.80997 -1.67839 -0.98252

C -8.46759 -2.27611 -2.36922

O -7.18453 -2.10893 -4.39366

C -9.59467 -2.77004 -0.21249

C -5.0312 -0.59156 -0.24164

C -5.18248 -0.43857 1.2638

C -3.90062 -0.37157 2.06258

C -6.37774 -0.45224 1.87824

C -3.96685 0.38864 -0.78359

C -2.86059 0.64502 1.52358

C -2.6592 0.37436 0.01442

C -3.3731 2.08184 1.75818

C -1.53827 0.41677 2.32358

C -0.37616 1.3806 2.06225

O 0.14127 1.25813 0.73157

C 1.07504 0.27611 0.54142

O 1.46275 -0.50278 1.40094

C 1.50845 0.26402 -0.93227

C 2.95699 -0.17591 -1.06406

O 1.38262 1.53481 -1.56805

H -4.62518 -1.60535 -0.38134

H -7.01013 -2.31224 -0.03797

C -9.78704 -0.50263 -1.07907

O -10.25978 0.12332 -0.14356

O -10.17096 -0.18634 -2.32886

H -6.18914 1.46189 -1.96393

H -7.84998 1.03315 -1.66668

H -6.82793 1.52418 -0.32009

H -5.69265 -2.15287 -2.30234

H -5.52906 -0.58927 -3.04966

H -7.94933 -0.4652 -3.46975

H -8.21648 0.25054 1.12761

H -8.27004 -1.37655 1.80139

H -9.37635 -2.45939 -2.95839

H -8.01976 -3.27247 -2.24046

H -8.01135 -2.20274 -4.89832

H -8.97572 -3.6563 -0.03358

H -9.95755 -2.41275 0.75708

H -10.47812 -3.08938 -0.77902

H -3.46097 -1.3789 2.04735

H -4.11402 -0.15121 3.11715

H -6.43764 -0.33754 2.95865

H -4.34384 1.4163 -0.78591

H -3.73328 0.14258 -1.82576

H -1.99697 1.12677 -0.42684

H -2.17164 -0.59915 -0.12612

H -4.38503 2.23333 1.3691

H -3.40287 2.31566 2.82849

H -2.72944 2.82089 1.26976

H -1.76874 0.47197 3.39609

H -1.1925 -0.60787 2.13509

H -0.68956 2.42004 2.19647

H 0.423 1.21346 2.79526

H 0.85292 -0.43722 -1.45972

H 3.11246 -1.17753 -0.65118

H 3.62324 0.5169 -0.53749

H 3.26583 -0.17853 -2.11483

H 0.52274 1.89718 -1.2828

H -10.80487 0.5512 -2.197

Standard orientation of (2'*S**)-**4g**

C -6.94581 1.01422 -1.12802

C -6.38755 -0.42853 -1.03795

C -6.16179 -0.96646 -2.47337

C -7.37467 -1.38184 -0.27205

C -7.4538 -1.24756 -3.23102

C -7.59382 -0.87816 1.17351

C -8.68877 -1.77889 -1.03564

C -8.33001 -2.23832 -2.47008

O -7.1086 -1.81226 -4.49512

C -9.36558 -2.98725 -0.34111

C -4.99473 -0.44947 -0.27103

C -5.12488 -0.42409 1.24429

C -3.83473 -0.31904 2.02574

C -6.30171 -0.57991 1.87428

C -4.02174 0.64988 -0.75229

C -2.88325 0.80739 1.54569

C -2.69858 0.66985 0.0184

C -3.49146 2.18162 1.90092

C -1.52889 0.62154 2.30081

C -0.41115 1.62421 1.99549

O 0.15591 1.42179 0.69265

C 1.12566 0.47178 0.60537

O 1.54529 -0.20492 1.53879

C 1.70504 0.3857 -0.8096

C 0.62733 0.18691 -1.86098

O 2.62707 -0.70154 -0.92178

H -4.51422 -1.41434 -0.49515

H -6.82438 -2.33354 -0.17067

C -9.75913 -0.68329 -1.02436

O -10.26293 -0.17492 -0.03526

O -10.19064 -0.30014 -2.23939

H -6.34699 1.63156 -1.80695

H -7.96244 1.04836 -1.52088

H -6.95477 1.51449 -0.15462

H -5.5727 -1.89375 -2.4375

H -5.55246 -0.26912 -3.06222

H -7.98258 -0.31216 -3.4341

H -8.20656 0.02662 1.20841

H -8.11562 -1.64592 1.75464

H -9.23393 -2.4492 -3.05745

H -7.80106 -3.20173 -2.42679

H -7.93571 -1.93484 -4.99272

H -8.67335 -3.83077 -0.24192

H -9.73433 -2.73662 0.65914

H -10.23285 -3.33383 -0.91654

H -3.32432 -1.28771 1.9289

H -4.04023 -0.19197 3.09707

H -6.34693 -0.55379 2.96117

H -4.47491 1.64246 -0.66436

H -3.79546 0.50585 -1.81494

H -2.10021 1.50123 -0.37018

H -2.14799 -0.25283 -0.20573

H -4.52134 2.28653 1.54494

H -3.51161 2.33135 2.98642

H -2.91594 3.00164 1.45952

H -1.72898 0.67104 3.37962

H -1.15525 -0.39093 2.10084

H -0.77681 2.65395 2.03644

H 0.37401 1.55463 2.75873

H 2.26548 1.30707 -0.99873

H -0.06266 1.03502 -1.89937

H 0.04453 -0.71772 -1.65406

H 1.07425 0.05832 -2.8526

H 2.97112 -0.86757 -0.02148

H -10.87849 0.37088 -2.03983

Standard orientation of (2'*S**)-**4h**

C -6.96682 1.07744 -1.08548

C -6.27618 -0.30883 -1.04098

C -6.02647 -0.7888 -2.49238

C -7.15552 -1.36809 -0.28314

C -7.29916 -1.17243 -3.23862

C -7.40013 -0.91685 1.17578

C -8.43846 -1.87352 -1.03529

C -8.07058 -2.25427 -2.49002

O -6.94093 -1.69517 -4.51758

C -8.97193 -3.16654 -0.36656

C -4.87521 -0.21769 -0.29442

C -4.98693 -0.21979 1.22516

C -3.7058 0.00947 1.99857

C -6.13471 -0.5006 1.86547

C -3.9975 0.95611 -0.79265

C -2.89678 1.23013 1.5104

C -2.68439 1.09319 -0.01526

C -3.68242 2.53188 1.81829

C -1.53691 1.35731 2.26985

C -0.61894 0.13241 2.34267

O -0.15543 -0.28923 1.05552

C 0.95847 0.3481 0.58502

O 1.54425 1.26071 1.15149

C 1.34036 -0.20926 -0.79265

C 0.96626 0.79077 -1.87312

O 0.73708 -1.46258 -1.10474

H -4.32141 -1.13947 -0.53187

H -6.51501 -2.26424 -0.21012

C -9.61399 -0.89406 -0.96268

O -10.1726 -0.51349 0.05466

O -10.0666 -0.45801 -2.15046

H -7.99202 1.0243 -1.45275

H -6.99702 1.55543 -0.10139

H -6.44549 1.76137 -1.76438

H -5.35227 -1.65681 -2.48906

H -5.49551 -0.02288 -3.07169

H -7.92371 -0.29264 -3.41624

H -8.10101 -0.07973 1.23652

H -7.83243 -1.74422 1.74851

H -7.46001 -3.16937 -2.48656

H -8.96521 -2.52428 -3.06757

H -6.51277 -0.97884 -5.01713

H -9.81798 -3.57792 -0.93081

H -8.19731 -3.93998 -0.31702

H -9.33225 -2.9884 0.65211

H -3.9075 0.10769 3.07306

H -3.11042 -0.90412 1.87831

H -6.17039 -0.49059 2.95306

H -4.53204 1.90845 -0.73196

H -3.75267 0.80996 -1.85101

H -2.08533 0.20581 -0.2404

H -2.12759 1.95672 -0.39931

H -3.80699 2.66716 2.89911

H -3.14985 3.4089 1.43305

H -4.68641 2.53847 1.38399

H -0.97519 2.18804 1.82298

H -1.74589 1.65044 3.30765

H 0.23378 0.34393 3.00034

H -1.13577 -0.71673 2.7996

H 2.42239 -0.3764 -0.78635

H 1.43656 1.76365 -1.69944

H -0.11861 0.93841 -1.9103

H 1.26996 0.42339 -2.85907

H -0.16898 -1.43055 -0.74416

H -10.82938 0.11296 -1.91783

Standard orientation of (2'*S**)-**4i**

C -7.03004 0.80708 -1.59817

C -6.41152 -0.46015 -0.95609

C -6.24702 -1.54463 -2.04973

C -7.31322 -1.01945 0.20294

C -7.568 -2.13076 -2.53464

C -7.47356 0.04077 1.31718

C -8.65146 -1.7177 -0.23201

C -8.36652 -2.72019 -1.37677

O -7.29074 -3.1842 -3.45722

C -9.22381 -2.54959 0.94458

C -4.97823 -0.14008 -0.34607

C -5.0279 0.53555 1.01866

C -3.7084 1.00884 1.59161

C -6.16183 0.63769 1.73311

C -4.06295 0.63471 -1.32525

C -2.86039 1.83362 0.59665

C -2.71504 1.01666 -0.70686

C -3.56942 3.18262 0.31185

C -1.47235 2.19548 1.21604

C -0.50973 1.04875 1.53547

O 0.06111 0.54804 0.32054

C 0.90507 -0.51559 0.4905

O 1.17277 -1.03253 1.56608

C 1.40527 -0.99336 -0.88073

C 2.8002 -1.58552 -0.77062

O 1.44976 0.04572 -1.85712

H -4.48301 -1.10563 -0.15862

H -6.71849 -1.83264 0.6542

C -9.76976 -0.73013 -0.57961

O -10.27007 0.08708 0.17778

O -10.24018 -0.82042 -1.83522

H -8.07098 0.66543 -1.8893

H -6.99389 1.67002 -0.92602

H -6.50273 1.08677 -2.51692

H -5.62181 -2.36588 -1.67187

H -5.69901 -1.14878 -2.91406

H -8.15042 -1.37873 -3.07395

H -8.12887 0.86176 1.01337

H -7.92237 -0.422 2.20258

H -7.80856 -3.58126 -0.9801

H -9.29841 -3.15334 -1.76522

H -6.85024 -2.78887 -4.22877

H -10.11319 -3.10991 0.63076

H -8.48965 -3.27234 1.31777

H -9.53134 -1.92052 1.78653

H -3.86781 1.59114 2.50857

H -3.16053 0.1081 1.89354

H -6.15342 1.12977 2.70372

H -4.54484 1.54831 -1.68503

H -3.86883 0.02064 -2.21216

H -2.17578 0.08296 -0.51138

H -2.12587 1.5779 -1.4424

H -4.59516 3.05861 -0.04773

H -3.62427 3.79403 1.22025

H -3.02295 3.75876 -0.44352

H -0.95894 2.88547 0.53254

H -1.64814 2.74751 2.14906

H 0.30403 1.44136 2.15722

H -1.00933 0.24433 2.08314

H 0.70232 -1.75382 -1.23749

H 3.51454 -0.83502 -0.41354

H 3.15487 -1.92275 -1.75041

H 2.82332 -2.43382 -0.07935

H 0.62483 0.55571 -1.74914

H -10.96132 -0.15645 -1.86551
